# The role of the SGK3/TOPK signaling pathway in the transition from acute kidney injury to chronic kidney disease

**DOI:** 10.3389/fphar.2023.1169054

**Published:** 2023-06-08

**Authors:** Huapan Shu, Yumei Wang, Hui Zhang, Qingqing Dong, Lulu Sun, Yuchi Tu, Qianqian Liao, Li Feng, Lijun Yao

**Affiliations:** ^1^ Department of Nephrology, Union Hospital, Tongji Medical College, Huazhong University of Science and Technology, Wuhan, Hubei, China; ^2^ Department of Nephrology, the Second Affiliated Hospital, Chongqing Medical University, Chongqing, China

**Keywords:** acute kidney injury, chronic kidney disease, macrophages, renal tubular epithelial cells, epithelial-to-mesenchymal transition, macrophage-to-myofibroblast transition

## Abstract

**Introduction:** Profibrotic phenotype of renal tubular epithelial cells (TECs) featured with epithelial to mesenchymal transition (EMT) and profibrotic factors secretion, and aberrant accumulation of CD206^**+**^ M2 macrophages are the key points in the transition from acute kidney injury (AKI) to chronic kidney disease (CKD). Nevertheless, the underlying mechanisms involved remain incompletely understood. Serum and glucocorticoid-inducible kinase (SGK) is a serine/threonine protein kinase, required for intestinal nutrient transport and ion channels modulation. T-LAK-cell-originated protein kinase (TOPK) is a member of the mitogen activated protein kinase family, linked to cell cycle regulation. However, little is known about their roles in AKI-CKD transition.

**Methods:** In this study, three models were constructed in C57BL/6 mice: low dose and multiple intraperitoneal injection of cisplatin, 5/6 nephrectomy and unilateral ureteral obstruction model. Rat renal tubular epithelial cells (NRK-52E) were dealt with cisplatin to induce profibrotic phenotype, while a mouse monocytic cell line (RAW264.7) were cultured with cisplatin or TGF-β1 to induce M1 or M2 macrophage polarization respectively. And co-cultured NRK-52E and RAW264.7 through transwell plate to explore the interaction between them. The expression of SGK3 and TOPK phosphorylation were detected by immunohistochemistry, immunofluorescence and western blot analysis.

**Results:** *In vivo*, the expression of SGK3 and p-TOPK were gradually inhibited in TECs, but enhanced in CD206^**+**^ M2 macrophages. *In vitro*, SGK3 inhibition aggravated epithelial to mesenchymal transition through reducing the phosphorylation state of TOPK, and controlling TGF-β1 synthesis and secretion in TECs. However, SGK3/TOPK axis activation promoted CD206^**+**^ M2 macrophage polarization, which caused kidney fibrosis by mediating macrophage to myofibroblast transition (MMT). When co-cultured, the TGF-β1 from profibrotic TECs evoked CD206^**+**^ M2 macrophage polarization and MMT, which could be attenuated by SGK3/TOPK axis inhibition in macrophages. Conversely, SGK3/TOPK signaling pathway activation in TECs could reverse CD206^**+**^ M2 macrophages aggravated EMT.

**Discussion:** We revealed for the first time that SGK3 regulated TOPK phosphorylation to mediate TECs profibrotic phenotype, macrophage plasticity and the crosstalk between TECs and macrophages during AKI-CKD transition. Our results demonstrated the inverse effect of SGK3/TOPK signaling pathway in profibrotic TECs and CD206^**+**^ M2 macrophages polarization during the AKI-CKD transition.

## 1 Background

Acute kidney injury (AKI) is a common kidney disease that, if not treated properly, can develop into chronic kidney disease (CKD) and result in the failed recovery of renal function (Forni et al., 2017; He et al., 2017; Humphreys, 2018; Ruiz-Ortega et al., 2020; Kellum et al., 2021). However, the specific mechanisms underlying the transition from AKI to CKD have not been fully examined. Recently, vast studies have confirmed that the profibrotic phenotype transition of TECs, mainly characterized by epithelial-to-mesenchymal transition (EMT) and the secretion of profibrotic factors, is the direct cause of the development of AKI to CKD (Ryoo et al., 2015; Venkatachalam et al., 2015; He et al., 2017; Dan et al., 2019; Li et al., 2020; Yamashita et al., 2020). Many reports have elucidated that a bunch of strong fibrogenic signaling pathways are persistently activated in these profibrotic TECs, which disrupts the normal repair of adjacent TECs and disturbs the differentiation of peripheral infiltrating macrophages, ultimately leading to the occurrence of EMT and macrophage-to-myofibroblast transition (MMT) (Gentle et al., 2013; Lovisa et al., 2015; Zuk and Bonventre, 2016; Canaud et al., 2019). It has been reported that in the early stage of AKI, M1 macrophages (characterized by iNOS and TNF-α expression) are dominant in the kidney exerting proinflammatory effects. Later, M2 macrophages (characterized by CD206 and Arg1 expression) replace M1 macrophages and mainly exert anti-inflammatory effects to repair the kidney (Spiller et al., 2015; Tan et al., 2019; Locati et al., 2020). However, the persistence of CD206^+^ M2 macrophages is closely related to MMT and promotes the development of kidney fibrosis (Cao et al., 2015; Meng et al., 2016a; Wang et al., 2017; Feng et al., 2018; Tang et al., 2019; Yuan et al., 2022). Nevertheless, to date, the specific molecular mechanisms underlying the EMT of TECs, macrophage alternative activation, and the crosstalk between TECs and macrophages have not been fully clarified.

Serum and glucocorticoid-inducible kinase (SGK) is a serine/threonine protein kinase, including three isoforms, namely, SGK1, SGK2, and SGK3. SGK1 and SGK3 are widely distributed in mammalian kidney tissue and are associated with the regulation of various cellular functions (Lang et al., 2006; Peng et al., 2018; Yuan et al., 2018). It has been reported that the inhibition of SGK1 can exacerbate the proinflammatory ability of microglia and negatively regulate TLR-induced inflammation (Zhou et al., 2015; Inoue et al., 2016). Additionally, our previous studies confirmed that SGK3 deletion could cause podocyte damage and increase proteinuria in mice (Dong et al., 2021). However, whether SGK3 is closely relevant to TECs and immune cells has not yet been explored. PDZ-binding kinase/T-LAK cell-originated protein kinase (PBK/TOPK) is a newly discovered serine–threonine kinase that mediates the inflammatory response by regulating the polarization of microglia cells and macrophages (Han et al., 2018). Since TOPK is mainly expressed in proliferative cells such as tumor cells and testicular tissues, rather than in normal tissues including kidneys, the function of TOPK in kidney biology has not been explored for a long time (Gaudet et al., 2000). To date, mounting compelling evidence has demonstrated that TOPK is not only involved in the pathogenesis of renal ischemia–reperfusion injury but also in the formation of renal calculi (Gao et al., 2016; Nettuwakul et al., 2020). Moreover, our recent study revealed that TOPK participates in cisplatin (CP)-induced apoptosis, oxidative stress, and cell cycle arrest of TECs in AKI (Zhang et al., 2022). However, whether TOPK is involved in the profibrotic phenotype transition of TECs and macrophage polarization during the AKI-to-CKD transition has not been reported.

In the present study, we investigated the effects of the SGK3/TOPK signaling pathway on the failure-repaired TECs, macrophage polarization, and the crosstalk between TECs and macrophages during the transition from AKI to CKD.

## 2 Materials and methods

### 2.1 Reagents and antibodies

Polyclonal rabbit anti-SGK3 (#8573), anti-Arg1 (#93668), and polyclonal rabbit anti-TOPK (#4942) antibodies were provided by Cell Signaling Technology (Beverly, MA, United States). Polyclonal rabbit anti-vimentin (10366-1-AP), polyclonal rabbit anti-E-cadherin (20874-1-AP), polyclonal rabbit anti-α-SMA (14395-1-AP), polyclonal rabbit anti-F4/80 (29414-1-AP), polyclonal rabbit anti-CD206 (18704-1-AP), polyclonal rabbit anti-iNOS (22226-1-AP), polyclonal rabbit anti-TNF-α (17590-1-AP), and polyclonal mouse anti-GAPDH (10494-1-AP) antibodies were purchased from Proteintech (Wuhan, China). Polyclonal rabbit anti-p-TOPK antibody (ab184953) was obtained from Abcam (Cambridge, England). Polyclonal rabbit anti-SGK3 antibody (12699-1-AP) for immunohistochemistry analysis was purchased from Proteintech (Wuhan, China), and polyclonal mouse anti-SGK3 antibody (SC-166847) for immunofluorescence analysis was purchased from Santa Cruz (California, America). Polyclonal chicken anti-F4/80 (ab186037) and polyclonal anti-p-TOPK (ab184953) antibodies for immunofluorescence analysis were provided by Abcam (Cambridge, England).

pcDNA3.1 (+)/mMOCK (MOCK) and pcDNA3.1 (+)/mTOPK-T9E (TOPK-T9E) plasmids were obtained from Qiuhong Duan (Department of Biochemistry and Molecular Biology, School of Basic Medicine, Huazhong University of Science and Technology, Wuhan, Hubei 430,022, China). pcDNA3.1/mSGK3-S486D (SGK3-S486D), and pcDNA3.1/mSGK3 wild-type (SGK3 wild-type) plasmids were gifts from David Pearce (Department of Medicine and Molecular and Cellular Pharmacology, University of California, San Francisco, CA, 94,107-2140, United States). The SGK3 small interfering RNA (siRNA) and TOPK short interfering RNA (shRNA) were constructed by RiboBio (Guangzhou, China). SGK3-PROTAC1 was purchased from Selleck (Shanghai, China). OTS964 was obtained from TargetMol (Shanghai, China). Cocktail and phosphatase inhibitors were purchased from Roche Applied Science (Indianapolis, IN, United States).

### 2.2 Experimental protocols for animals

Eight-week-old male C57BL/6 mice (Charles River, Beijing, China) weighing 22–28 g were housed in an air-filtered, pathogen-free environment under a constant-temperature and -humidity environment with a 12 h light/dark cycle. The animals were allowed free access to water and food. The mice were used in three models, namely, multiple and low-dose cisplatin by intraperitoneal injection, 5/6 nephrectomy (5/6 Nx), and unilateral ureteral obstruction (UUO). For multiple and low-dose cisplatin administration, mice were intraperitoneally injected with cisplatin (CP, 8 mg/kg, P4394, Sigma-Aldrich, United States) or equal volume of normal saline (NS) on days 0, 7, 14, and 21 and then euthanized on day 28. The blood samples and kidneys of mice were collected when euthanized. Furthermore, the body weights of mice were measured when the mice were euthanized. According to the number of CP injections, the mice were randomly divided into four groups, namely, CON (NS, NS, NS, and NS), 2CP (NS, NS, CP, and CP), 3CP (NS, CP, CP, and CP), and 4CP (CP, CP, CP, and CP). For 5/6 Nx, the mice were randomly divided into two groups, namely, the normal control sham and 5/6 Nx groups. Following the previously described methods (Nishiyama et al., 2019), the mice from the CKD group underwent 5/6 nephrectomy in two stages. Under general anesthesia with lateral incision to expose the retroperitoneal kidney, the mice were removed from two-thirds of the left kidney, and then, a week later, the mice were accepted for resection of the right kidney *via* a flank incision. Meanwhile, the sham control group received the same incision and kidney exposure under general anesthesia. All mice were euthanized 8 weeks after the operation, and then, blood and residual kidneys were harvested for subsequent analyses. For the UUO model, the mice were randomly assigned to four groups, namely, the sham control group and the 3, 7, and 14 days after UUO surgery groups. The left ureter was exposed after anesthesia and ligation of the left lower pole of the kidney and the upper 1/3 of the ureter with 4–0 sutures. The ureter was then cut between the two ligation points. The mice were euthanized at 3, 7, and 14 days after the operation, and then, blood and the obstructed kidney were collected for various analyses. All the collected blood samples were used for testing the level of serum creatinine (Scr) and blood urea nitrogen (BUN). Furthermore, the collected mouse kidneys underwent Western blotting (WB), hematoxylin and eosin (HE) staining, Masson’s trichrome staining, immunohistochemical (IHC) staining, immunofluorescence (IF), and other experiments. All animal procedures received approval from the Care and Use Committee of the Animal Laboratory Center of Tongji Medical College, Huazhong University of Science and Technology.

### 2.3 Measurement of serum creatinine, blood urea nitrogen, and estimated glomerular filtration rate

Serum creatinine (Scr) and blood urea nitrogen (BUN) were quantified using the respective kits (Nanjing Jiancheng Bioengineering Institute, Nanjing, China) according to the manufacturer’s instructions. The estimated glomerular filtration rate (eGFR) was calculated by the body weight (BW) of mice according to a previous report in which the dependence of GFR on BW was demonstrated to be GFR = 0.036*BW^0.59 (0.74 ± 0.15) (GFR is measured in ml/min and BW in g) (Hackbarth et al., 1982).

### 2.4 Morphometric analysis, immunohistochemistry, and immunofluorescence of the mouse kidney

Mouse kidneys were fixed with 4% paraformaldehyde and embedded in paraffin before being prepared into 4 µm sections. Staining with hematoxylin and eosin (HE) was performed using the HE Stain Kit (Vector Laboratories), and the damage scores of tubules were graded according to the percentage of tubular dilatation, tubular casts, TEC necrosis, and loss of brush border. Ten high-magnification visual fields in the cortex and the outer stripe of the outer medulla were collected for evaluation. The presence of pathological changes was graded according to the previous report (Molitoris et al., 2009). Masson’s staining was performed with the Trichrome Stain Kit (Abcam). The collagen fibers were stained blue, and muscle fibers were stained red when observed under the microscope. Each section was selected with 10 independent visual fields randomly under 400 light lenses. Quantification of the fibrotic area was carried out by using Image-Pro Plus version 6.0 software (Bethesda, MD, United States) (Feng et al., 2018). Immunohistochemical staining was performed and analyzed essentially as previously described (Peng et al., 2018). The kidney slides were incubated with rabbit polyclonal anti-SGK3 , rabbit polyclonal anti-p-TOPK , rabbit polyclonal anti-F4/80, and rabbit polyclonal anti-α-SMA antibodies at 4°C overnight, followed by incubation with polyperoxidase anti-rabbit secondary antibody for 60 min at room temperature. Then, a DAB kit (PV-6001, ZSGB-BIO, Beijing, China) was used for staining. Histological examination was performed *via* light microscopy. For immunofluorescence analysis, kidneys were snap frozen immediately, and sections were cut into 4 μm, blocked with 10% donkey serum for 30 min, rinsed in PBST, and then incubated with rabbit polyclonal anti-SGK3, mouse polyclonal anti-CD206, and chicken polyclonal anti-F4/80 antibodies at 4°C overnight. Then, the sections were stained with Cy3-conjugated AffiniPure goat anti-mouse IgG (H + L) (SA00009-1; Proteintech, China), AMCA-conjugated AffiniPure goat anti-rabbit IgG (H + L) (SA00010-2, Proteintech, China), and goat anti-chicken IgY H&L (Alexa Fluor^®^ 488, Abcam, England) antibodies.

### 2.5 Cell culture and chemical treatment

Rat renal tubular epithelial cells (NRK-52E) with normal morphological and biological characteristics and a mouse monocytic cell line (RAW264.7) were all obtained from the cell bank of the Chinese Academy of Sciences (Shanghai, China). Both cell lines were cultured in DMEM with 10% fetal bovine serum (FBS) and 1% penicillin–streptomycin at 37°C in a constant-temperature incubator containing 5% CO_2_. NRK-52E cells were treated with three or five times of CP (5 µM) (P4394, Sigma-Aldrich, United States) every 6 h and harvested 3 h after the last treatment or one-time CP (20 µM) stimulation for 24 h. M1 macrophages were activated by different concentrations of CP (5, 10, and 15 µM) for 24 h or multiple and low-dose of CP administration as described previously. Additionally, M1 macrophages were also activated by LPS (100 ng/mL) (297-473-0, Sigma-Aldrich, United States) for 24 h. M2 macrophages were stimulated with TGF-β1 (HZ-1011, Proteintech, China) (5 or 10 ng/mL) for 24 h. In this study, the concentrations of SGK3-PROTAC1 and OTS964 were 3 μM and 5 µM, respectively. All cells were cultured in serum-free media for 12 h before being treated with reagents.

### 2.6 Bone marrow-derived macrophage isolation, culture, differentiation, and chemical treatment

Bone marrow-derived macrophages (BMDMs) from mice were prepared as previously described (Liu et al., 2015). The BMDMs were cultured in DMEM with 10% (FBS) and 1% penicillin–streptomycin at 37°C in a constant-temperature incubator containing 5% CO_2_. The BMDMs were differentiated by 50 ng/mL macrophage colony-stimulating factor (M-CSF) (SRP3221, Sigma-Aldrich, United States) for 5 days. The concentrations of SGK3-PROTAC1 and OTS964 used in BMDMs were 3 μM and 5 µM, respectively.

### 2.7 Cell transfection

When NRK-52E cells, RAW264.7 cells, and BMDMs reached approximately 50% confluence, they were transfected with various plasmids for 48 h by Lipofectamine 2000 Reagent according to the manufacturer’s instructions (Invitrogen, Carlsbad, CA, United States), and the cell lysates were used for immunoblotting analysis. The NRK-52E or RAW264.7 cells were transfected with SGK3 siRNA or TOPK shRNA for 48 h, and then the cell lysates were used for immunoblotting analysis.

### 2.8 Western blot analysis

Cells were resuspended by radioimmunoprecipitation assay (RIPA) buffer (Beyotime, China) containing 1 mM PMSF, 1 mM cocktail, and 1 mM phosphatase inhibitor. The lysates were centrifuged at 4°C and 12,000 r for 15 min, and the protein concentration was determined using a BCA protein assay kit (Thermo Scientific, United States). The samples were isolated by electrophoresis on SDS-polyacrylamide gel (8%–13%), and then the proteins were transferred to a polyvinylidene fluoride (PVDF) membrane (Millipore Corp, United States). Membranes were blocked in 5% BSA for 1 h at room temperature and then incubated with the respective antibodies in a 4°C constant-temperature refrigerator overnight. The next day, membranes were incubated with corresponding horseradish peroxidase-conjugated secondary antibodies for 1 h at temperature following imaging.

### 2.9 Flow cytometry

After different treatments, the RAW264.7 cells were centrifuged and resuspended in PBS for flow cytometry experiments. According to the manufacturer’s instructions (Peng et al., 2019), anti-mouse F4/80 (565,410; Biosciences, Shanghai) and anti-mouse iNOS antibodies (696,805; BioLegend, Beijing) were used for fluorescent staining. Isotype antibody controls were used to exclude non-specific fluorescent staining. Data were obtained using a CytoFLEX flow cytometer (Beckman Coulter, United States).

### 2.10 Enzyme-linked immunosorbent assay

The level of TGF-β1 in the supernatant of the NRK-52E cell culture medium was measured following the protocols of the TGF-β1 ELISA kits (KE10005, Proteintech, China).

### 2.11 Quantitative real-time polymerase chain reaction

The RNA of NRK-52E and RAW264.7 cells was extracted by TRIzol reagent (Novizan, China) according to the manufacturer’s instructions and was reverse transcribed into cDNA by PrimeScript RT Master Mix (TaKaRa, Dalian, China). After that, quantitative real-time polymerase chain reaction (RT-qPCR) was conducted using the StepOnePlus Real-Time PCR System (Applied Biosystems, United States) using SYBR Premix Ex Taq II (Novizan, China). The PCR results of each sample were standardized using β-actin mRNA as an internal control. The experimental data of the RT-qPCR were analyzed using the algorithm of the 2^**−ΔΔCT**^ method.

The relative sequences of the primer pairs used in RT-qPCR are as follows: mouse β-actin forward: TGC​TGT​CCC​TGT​ATG​CCT​CTG, reverse: TGA​TGT​CAC​GCA​CGA​TTT​CC; mouse iNOS forward: CAG​ATC​GAG​CCC​TGG​AAG​AC, reverse: CTG​GTC​CAT​GCA​GAC​AAC​CT; mouse iNOS forward: CAT​CTT​CTC​AAA​ATT​CGA​GTG​ACA​A, reverse: TGG​GAG​TAG​ACA​AGG​TAC​AAC​CC; rat β-actin forward: GTG​ACG​TTG​ACA​TCC​GTA​AAG​A, reverse: GCC​GGA​CTC​ATC​GTA​CTC​C; and rat TGF-β1 forward: GCT​CCC​CTA​TTT​AAG​AAC​ACC​CAC, reverse: CTC​CCA​AGG​AAA​GGT​AGG​TGA​TAG.

### 2.12 Immunofluorescence

The cells were washed with PBS three times, and then fixed with 4% paraformaldehyde for 30 min at room temperature. Then, the cells were permeabilized with 0.1% Triton X-100 in PBS for 10 min and sealed by 5% BSA for 30 min. Finally, cells were incubated overnight with various antibodies at 4°C. The following day, the cells were stained with Cy3-conjugated AffiniPure goat anti-mouse IgG (H + L) (SA00009-1; Proteintech, Wuhan, China) and fluorescein (FITC)-conjugated AffiniPure goat anti-rabbit IgG (H + L) (SA00003-2, Proteintech, Wuhan, China) at room temperature for 1 h. Nuclei were co-stained with 4,6-diamidino-2-phenylindole (DAPI) for 7 min. Images were taken using a fluorescent microscope (Nikon, Tokyo, Japan).

### 2.13 Statistical analysis

All data were expressed as the mean ± SE, and SPSS 22.0 software was used for analyzing. The whole statistical analysis was processed using GraphPad Prism 5.0. The unpaired *t*-test was used as the analysis between two groups, while the one-way analysis of variance (ANOVA) with Dunnett’s post-hoc test was applied for comparisons among multifarious groups. Statistical significance was set at *p* < 0.05.

## 3 Results

### 3.1 SGK3 and p-TOPK are downregulated in TECs treated with multiple and low-dose cisplatin *in vivo*

**FIGURE 1:**
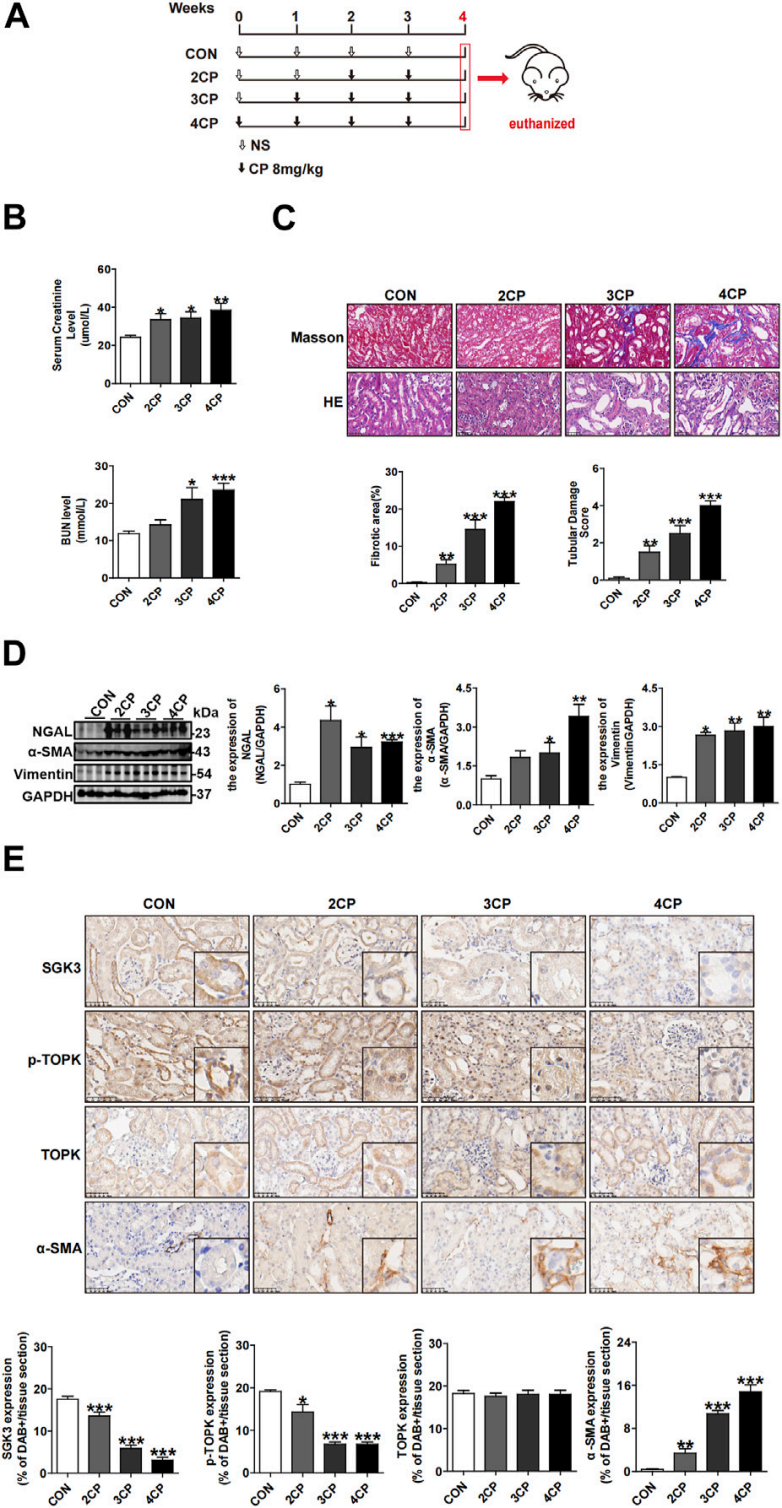
SGK3 and p-TOPK are downregulated in TECs treated with multiple and low-dose cisplatin *in vivo.* **(A)** Animal experimental design: Mice were intraperitoneally injected with 8 mg/kg dose of CP for 0, 2, 3, and 4 times, once a week and euthanized 1 week after the last injection. According to the times of CP exposure, the mice were grouped into CON, 2CP, 3CP, and 4CP. **(B)** Scr and BUN were measured in mice after the administration with CP (n = 6). **(C)** H&E staining and tubular damage score demonstrating tubular damage. Tubular injury was defined as tubular casts, tubular dilatation, tubular cell abscission, and loss of brush border. Masson’s trichrome staining and graphic presentation showed fibrotic area of CP-induced AKI-CKD mice. *p < 0.05, **p < 0.01, and ***p < 0.001 vs. the control group (n = 6). Scale bar, 50 μm. **(D)** Western blotting analysis of NGAL, α-SMA, vimentin in kidney cortex of CP-induced AKI-CKD mice. *p < 0.05, **p < 0.01, and ***p < 0.001 vs. the control group (n = 3). **(E)** Immunohistochemical (IHC) staining for SGK3, p-TOPK, TOPK, and α-SMA in the kidney section of CP-stimulated AKI-CKD mice. *p < 0.05, **p < 0.01, and ***p < 0.001 vs. the control group (n = 6). Scale bar, 50 μm.

To obtain a mouse model of AKI–CKD transition, multiple and low-dose cisplatin was injected intraperitoneally into mice (Sharp et al., 2016; Fu et al., 2018; Su et al., 2020), as shown in Figure 1A. Compared with the control, the serum levels of creatinine and urea nitrogen increased gradually following repeated and low-dose CP injection (Figure 1B). Moreover, the estimated glomerular filtration rate (eGFR) of mice decreased gradually, as shown in Supplementary Figures S1. Kidney histological examination revealed severe tubular dilation and swelling on day 14 (2CP), and injured tubular cells representing atrophic and flattening epitheliums along with interstitial fibrosis were observed on day 21 (3CP) and were more obvious on day 28 (4CP) (Figure 1C). Furthermore, immunoblotting analysis showed that the renal protein expression levels of neutrophil gelatinase-associated lipocalin (NGAL), vimentin, and α-smooth muscle actin (α-SMA) (Figure 1D) were notably enhanced in the repeated CP exposure groups. Additionally, the expression of α-SMA increased gradually, which was detected by immunohistochemical staining (Figure 1E). All of the aforementioned results indicated that the repeated and low-dose CP-induced mouse AKI–CKD transition model was successfully set up. Our previous research demonstrated that the phosphorylation level of TOPK at Thr9 (p-TOPK) rather than TOPK had significant alteration staining in TECs with the administration of CP (Zhang et al., 2022). To investigate the role of SGK3 and TOPK in the CP-induced AKI–CKD transition, we tested the expression of SGK3, p-TOPK, and TOPK in mouse kidneys injected with CP. Figure 1E shows that SGK3 and p-TOPK but not TOPK gradually decreased in TECs being injected with repeated CP, suggesting the relevance of SGK3 and p-TOPK in TEC injury during the progression from AKI to CKD.

### 3.2 SGK3/TOPK signaling pathway inhibition exacerbated cisplatin-induced EMT of NRK-52E cells

**FIGURE 2:**
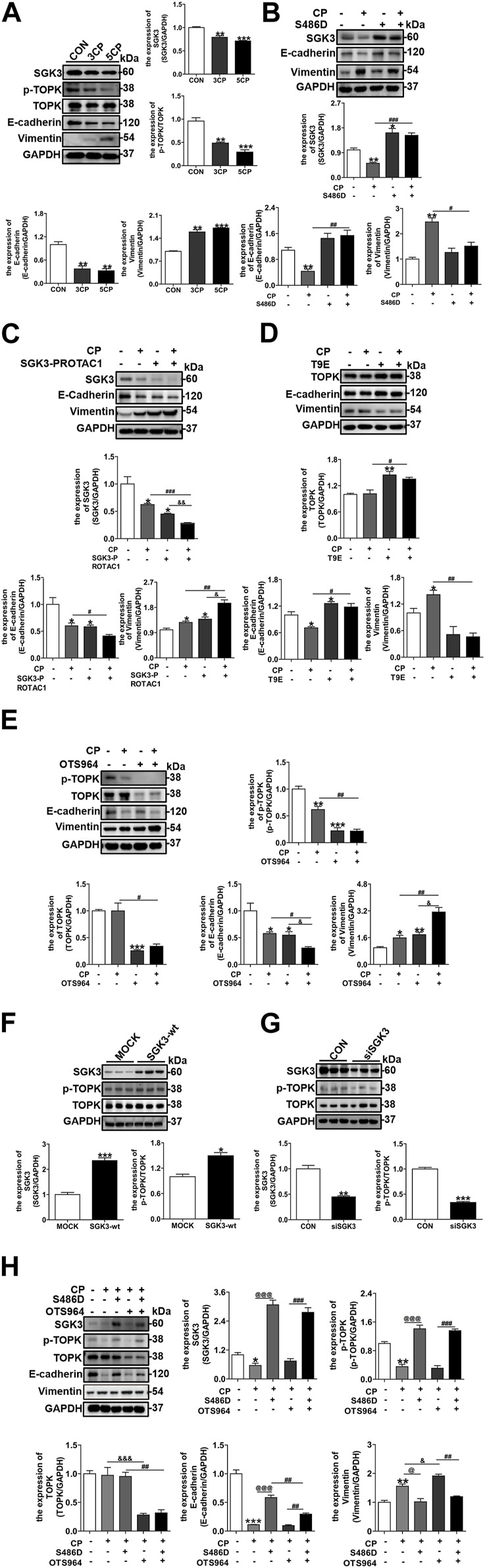
SGK3/TOPK signaling pathway inhibition exacerbated cisplatin-induced EMT of NRK-52E cells. **(A)** SGK3, p-TOPK, TOPK, E-cadherin, and vimentin were detected by Western blotting in NRK-52E with three or five times of CP (5 μM) stimulation. *p < 0.05, **p < 0.01, and ***p < 0.001 vs. the control group (n = 3). **(B)** NRK-52E cells were transiently transfected with SGK3-S486D plasmids for 48 h, and CP (20 μM) was applied within the last 24 h. Immunoblotting analysis for SGK3, E-cadherin, and vimentin expression. **p < 0.01 vs. the control group; ^**#**^ *p* < 0.05, ^**##**^ *p* < 0.01, and ^**###**^ *p* < 0.001 vs. the CP+/S486D group (n = 3). **(C)** NRK-52E cells were treated with CP and SGK3-PROTAC1 for 24 h and immunoblotting analysis for SGK3, E-cadherin, and vimentin expression. *p < 0.05 vs. the control group; ^**#**^ *p* < 0.05, ^**##**^ *p* < 0.01, and ^**###**^ *p* < 0.001 vs. the CP+/S486D group; ^&^ *p* < 0.05 and ^&&^ *p* < 0.01 vs. the CP-/SGK3-PROTAC + group (n = 3). **(D)** NRK-52E cells were transiently transfected with TOPK-T9E plasmid for 24 h and treated with CP for another 24 h, and the abundance of TOPK, E-cadherin, and vimentin was evaluated by Western blotting. *p < 0.05 and **p < 0.01 vs. the control group; ^**#**^ *p* < 0.05 and ^**##**^ *p* < 0.01 vs. the CP+/T9E group (n = 3). **(E)** NRK-52E cells were treated with CP and OTS964 for 24 h, and the expression of p-TOPK, TOPK, E-cadherin, and vimentin was evaluated by Western blotting. *p < 0.05, **p < 0.01, and ***p < 0.001 vs. the control group; ^**#**^ *p* < 0.05 and ^**##**^ *p* < 0.01 vs. the CP+/OTS964 group; ^&^ *p* < 0.05 and ^&&^ *p* < 0.01 vs. the CP/OTS964+ group (n = 3). **(F)** Immunoblotting analysis for SGK3, p-TOPK, and TOPK expression in NRK-52E transfected with SGK3 wild-type plasmids for 48 h. *p < 0.05 and ***p < 0.001 vs. the mock group (n = 3). **(G)** NRK-52E cells were transiently infected with SGK3 siRNA (30 nM) for 48 h, and then the expression of SGK3, p-TOPK, and TOPK was measured by Western blotting. **p < 0.01 and ***p < 0.001 vs. the control group (n = 3). **(H)** After SGK3-S486D plasmid transfection for 24 h, NRK-52E cells were exposed to CP and OTS964 for another 24 h, and then, the abundance of SGK3, p-TOPK, TOPK, E-cadherin, and vimentin was measured by Western blotting. *p < 0.05, **p < 0.01, and ***p < 0.001 vs. the control group; ^**@@**^ *p* < 0.01 and ^**@@@**^ *p* < 0.001 vs. the CP+/S486D-/OTS964 +group; ^&^ *p* < 0.05 and ^&&&^ *p* < 0.001 vs. the CP+/S486D-/OTS964 + group; ^**##**^ *p* < 0.01 and ^**###**^ *p* < 0.001 vs. the CP+/S486D+/OTS964 + group (n = 3).

To delineate the mechanism driving the AKI–CKD transition, multiple and low-dose CP was applied to cultured NRK-52E cells *in vitro*. As shown in Figure 2A, repeated and low-dose CP exposure increasingly inhibited SGK3 and p-TOPK expression and gradually aggravated NRK-52E EMT. Since the expression of SGK3 and p-TOPK presented continuous decreases under multiple times of low-dose CP applications, we used single CP stimulation thereafter to investigate the effects of SGK3 and p-TOPK on CP-induced NRK-52E EMT. NRK-52E cells were transfected with an SGK3 persistently activated plasmid, SGK3-S486D, followed by CP stimulation. Within expectation, SGK3 activation could confront the occurrence of CP-induced NRK-52E EMT (Figure 2B). On the contrary, using the SGK3 inhibitor, SGK3-PROTAC1 could deteriorate CP-induced EMT (Figure 2C). Similarly, the overexpression of TOPK with an activated TOPK-T9E plasmid and inhibition of TOPK inhibitor OTS964 in NRK-52E cells could counteract and exacerbate CP-induced EMT, respectively (Figures 2D,E). All these findings implied that SGK3 and TOPK were protective against CP-induced injury in NRK-52E cells.

Multiple studies have shown that AKT is the downstream of TOPK (Huang et al., 2021; Zhang et al., 2022); however, as a kinase sharing 55% of its domain with AKT (Tessier and Woodgett, 2006), whether SGK3 is related to TOPK has not been studied. We further explored the relationship between SGK3 and TOPK and their role in CP-triggered EMT of NRK-52E cells. First, NRK-52E cells were transfected with SGK3 wild-type plasmids and SGK3 siRNA (30 nM), respectively, *in vitro*. Immunoblotting analysis revealed an increase in p-TOPK expression in SGK3-overexpressing NRK-52E cells and a decrease in p-TOPK expression in SGK3-downregulated NRK-52E cells, indicating that SGK3 regulates the phosphorylation of TOPK (Figures 2F,G). Second, SGK3-S486D plasmid-transfected NRK-52E cells were treated with CP and OTS964 together. Western blotting analysis revealed that the inhibition of TOPK blocked SGK3 activation, alleviating CP-induced NRK-52E EMT (Figure 2H). Taken together, the inhibition of the SGK3/TOPK axis promoted CP-induced TEC EMT.

### 3.3 Inactivation of the SGK3/TOPK signaling pathway drives cisplatin-induced M1 macrophage polarization

**FIGURE 3:**
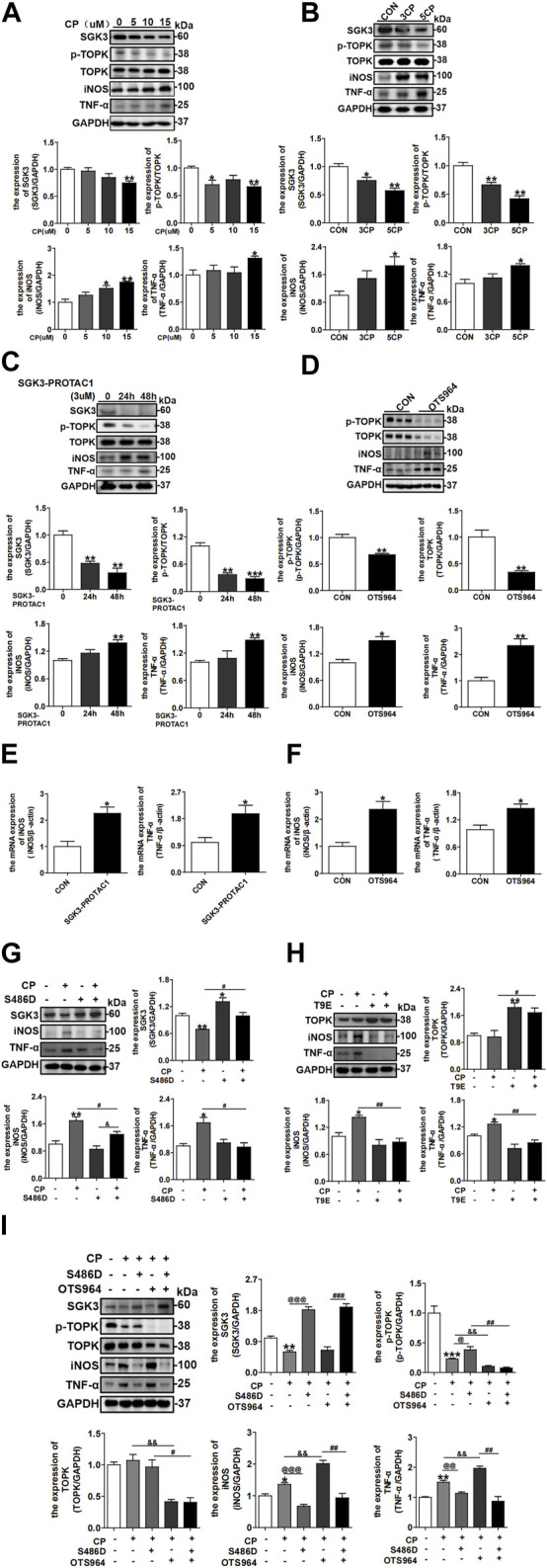
Inactivation of the SGK3/TOPK signaling pathway drives cisplatin-induced M1 macrophage polarization. **(A)** Western blotting analysis of SGK3, p-TOPK, TOPK, iNOS, and TNF-α in RAW264.7 cells treated with 0, 5, 10, and 15 μM CP for 24 h. *p < 0.05 and **p < 0.01 vs. the control group (n = 3). **(B)** RAW264.7 cells were exposed to three or five times of CP (5 μM), and the expression of SGK3, p-TOPK, TOPK, iNOS, and TNF-α was measured by Western blotting. *p < 0.05 and **p < 0.01 vs. the control group (n = 3). **(C)** RAW264.7 were treated with SGK3-PROTAC1 for 24 h or 48 h, and immunoblotting analysis for the abundance of SGK3, p-TOPK, TOPK, iNOS, and TNF-α. **p < 0.01 and ***p < 0.001 vs. the 0 h group (n = 3). **(D)** OTS964 was given to RAW264.7 for 24 h, and the expression of p-TOPK, TOPK, iNOS, and TNF-α was gauged by Western blotting. *p < 0.05 and **p < 0.01 vs. the control group (n = 3). **(E, F)** The RT-qPCR evaluated the mRNA level of iNOS and TNF-α in RAW264.7 cells treated by SGK3-PROTAC1 or OTS964 for 24 h. *p < 0.05 vs. the control group (n = 3). **(G)** RAW264.7 cells were transfected with SGK3-S486D plasmids for 24 h, and then, CP was added for another 24 h. The expression of SGK3, iNOS, and TNF-α was analyzed by Western blotting. *p < 0.05 and **p < 0.01 vs. the control group; ^**#**^ *p* < 0.05 vs. the CP+/S486D group; ^&^ *p* < 0.05 vs. the CP-/S486D + group (n = 3). **(H)** TOPK-T9E plasmid was transfected in RAW264.7 cells for 48 h, and CP was applied within the last 24 h; then, the abundance of TOPK, iNOS, and TNF-α was presented by immunoblotting. *p < 0.05 and **p < 0.01 vs. the control group; ^**#**^ *p* < 0.05 and ^**##**^ *p* < 0.01 vs. the CP+/T9E group (n = 3). **(I)** RAW264.7 cells were transfected with SGK3-S486D plasmid for 48 h and CP, OTS964 were added in the last 24 h. Western blotting analysis of SGK3, p-TOPK, TOPK, iNOS, and TNF-α. *p < 0.05, **p < 0.01, and ***p < 0.001 vs. the control group; ^**@**^ *p* < 0.05, ^**@@**^ *p* < 0.01, and ^**@@@**^ *p* < 0.001 vs. the CP+/S486D-/OTS964 group; ^&&^ *p* < 0.01 vs. the CP+/S486D-/OTS964 + group; ^**#**^ *p* < 0.05, ^**##**^ *p* < 0.01, and ^**###**^ *p* < 0.001 vs. the CP+/S486D+/OTS964 + group (n = 3).

Considering that SGK1 and TOPK regulate macrophage polarization (Inoue et al., 2016; Han et al., 2018) and that CP treatment results in M1 macrophage polarization (Tan et al., 2019), we investigated the role of the SGK3/TOPK signaling pathway in CP-induced macrophage phenotype switching. RAW246.7 cells were treated with different concentrations of CP for 24 h, and the protein expression levels of SGK3, TOPK, p-TOPK, iNOS, and TNF-α were detected by immunoblotting. As shown in Figure 3A, the levels of iNOS and TNF-α representing M1 macrophages were significantly upregulated in CP-treated groups compared with the control group. In addition, the expression levels of SGK3 and p-TOPK were decreased in a dose-dependent manner after CP administration. Consistently, repeated and low-dose CP stimulation resulted in a gradual downregulation of SGK3 and p-TOPK expression, followed by a growing M1 macrophage transformation (Figure 3B). Subsequently, we treated RAW264.7 cells with SGK3-PROTAC1 for 24 h and 48 h, or OTS964 for 24 h, and observed that the protein and mRNA levels of iNOS and TNF-α were significantly increased (Figures 3C–F). Additionally, we found that the stimulation of SGK3-PROTAC1 and OTS964 in RAW264.7 cells significantly increased the proportion of M1 macrophages (F4/80+ and iNOS+), which was determined by flow cytometry, as shown in Supplementary Figure S6. In addition, we treated BMDMs with SGK3-PROTAC1 or OTS964 for 24 h and found that the protein levels of iNOS and TNF-α were distinctly upregulated (Supplementary Figures S5A,B), which is consistent with the results in RAW264.7 cells. Nevertheless, SGK3 or TOPK activation could reverse CP-induced M1 macrophage polarization (Figures 3G,H). Finally, RAW264.7 cells were transfected with SGK3-S486D for 24 h and then treated with OTS964 and CP for another 24 h. Western blotting demonstrated that SGK3 activation alleviated TOPK inhibition, exacerbating CP-induced M1 macrophage polarization (Figure 3I). Collectively, these data suggested that the blockade of the SGK3/TOPK signaling pathway in macrophages promoted CP-induced M1 macrophage polarization.

### 3.4 The SGK3/TOPK signaling pathway is upregulated in CD206^+^ M2 macrophages while downregulated in TECs during the AKI-to-CKD transition

**FIGURE 4:**
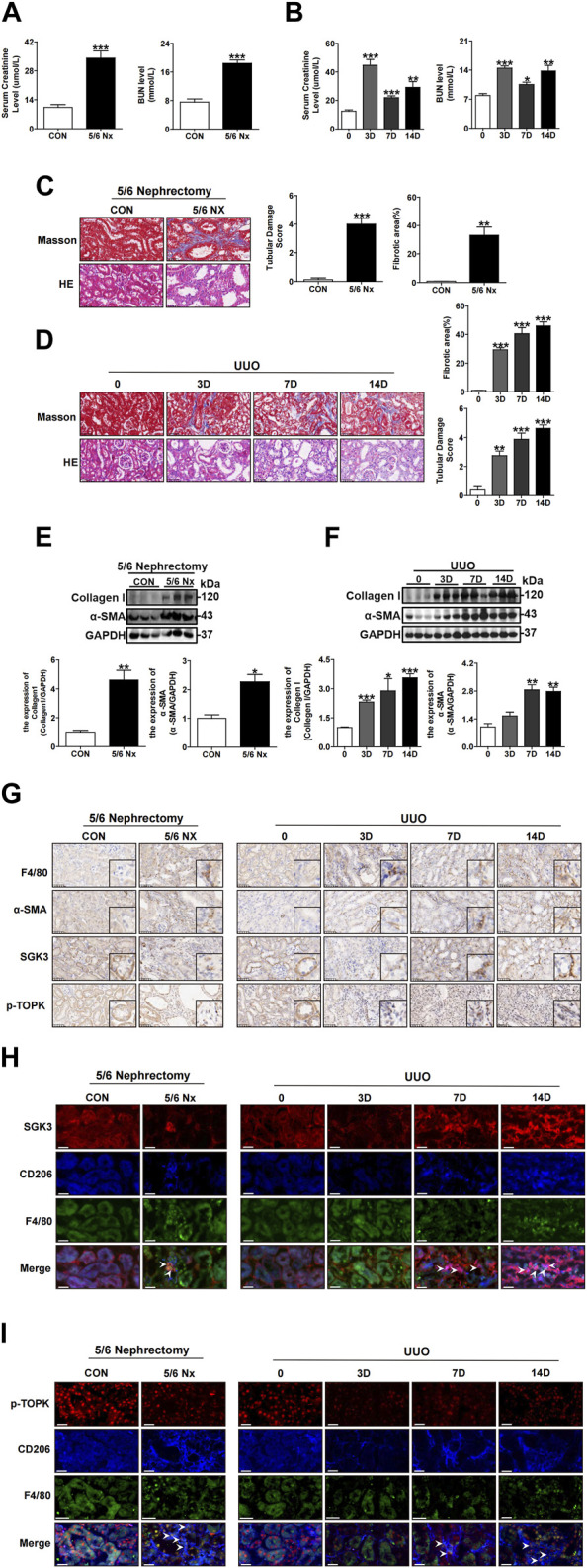
The SGK3/TOPK signaling pathway is upregulated in CD206^**+**^ M2 macrophages while downregulated in TECs during the AKI-to-CKD transition. **(A)** Scr and BUN from the CON and 5/6 Nx groups after 5/6 nephrectomy surgery. ***p < 0.001 vs. the control group (n = 4). **(B)** Scr and BUN were assessed at 0, 3, 7, and 14 days after UUO surgery. *p < 0.05, **p < 0.01, and ***p < 0.001 vs. the control group (n = 4). **(C)** H&E staining and tubular damage score demonstrating tubular damage; Masson’s trichrome staining and graphic presentation showing fibrotic areas of the CON and 5/6 Nx groups after 5/6 nephrectomy surgery. **p < 0.01 and ***p < 0.001 vs. the control group (n = 4). Scale bar, 50 μm. **(D)** H&E staining and tubular damage score demonstrating tubular damage; Masson’s trichrome staining and graphic presentation showing fibrotic areas of 0, 3, 7, and 14 days after UUO surgery. **p < 0.01 and ***p < 0.001 vs. the 0 D group (n = 4). Scale bar, 50 μm. **(E)** Western blotting analysis for collagen I and a-SMA protein levels in the CON and 5/6 Nx groups after 5/6 nephrectomy surgery. *p < 0.05 and **p < 0.01 vs. the control group (n = 3). **(F)** Western blotting analysis for collagen I and a-SMA protein levels in mice at 0, 3, 7, and 14 days after UUO surgery. *p < 0.05, **p < 0.01, and ***p < 0.001 vs. the 0D group (n = 3). **(G)** Immunohistochemical staining for F4/80, α-SMA, SGK3, and p-TOPK in CON, 5/6 Nx group kidney sections after 5/6 nephrectomy surgery and in 0, 3, 7, and 14 days of kidney sections after UUO surgery. Scale bar, 50 μm. **(H, I)** Representative immunofluorescent images showed staining with specific antibodies to SGK3 (red) or p-TOPK (red), CD206 (blue) and F4/80 (green) in renal sections of CON, and 5/6 Nx groups after 5/6 nephrectomy surgery and in kidney sections of 0, 3, 7, and 14 days mice after UUO surgery. Scale bar, 10 μm.

Since abnormal accumulation of CD206^**+**^ M2 macrophages and profibrotic TECs are key points for accelerating the transition from AKI to CKD (Cao et al., 2015; Venkatachalam et al., 2015; Wang et al., 2017; Feng et al., 2018; Tang et al., 2019), we further examined whether the SGK3/TOPK signaling pathway was linked to renal failure recovery. First, two classic kidney fibrosis models, namely, UUO and 5/6 Nx, were established (Chevalier et al., 2009; Nishiyama et al., 2019). The Scr, BUN, and tubular damage shown by H&E staining were more severe in the experimental group than in the control group (Figures 4A–D). Furthermore, the values of eGFR were decreased significantly in the 5/6 Nx group and UUO for 7 and 14 days groups when compared to the control group (Supplementary Figure S1). Notably, Masson’s trichrome staining showed that injured tubules were surrounded by fibrosis lesions both in the UUO and in 5/6 Nx mice (Figures 4C,D). In addition, immunoblotting analysis indicated that the protein levels of collagen I and α-SMA also dramatically increased in the UUO and 5/6 Nx groups when compared with the control groups (Figures 4E,F). Second, the expression and location of SGK3 and p-TOPK in kidneys of AKI-CKD mice were detected by immunohistochemistry (Figure 4G), and confocal immunofluorescence examination of SGK3 is exhibited in Figures 4H,I. We observed that the expression of SGK3 and p-TOPK was reduced in injured TECs, while they were gradually detected in the interstitial fibrosis region (Figure 4G). In addition, SGK3 and p-TOPK in the interstitial fibrosis region were mostly colocalized with CD206^**+**^ M2 macrophages (Figures 4H,I). Collectively, the aforementioned results indicated that the SGK3/TOPK signaling pathway might be involved in CD206^**+**^ M2 macrophage polarization and injured tubules.

### 3.5 Inhibition of the SGK3/TOPK signaling pathway alleviates TGF-β1-induced CD206^+^ M2 macrophage transition

**FIGURE 5:**
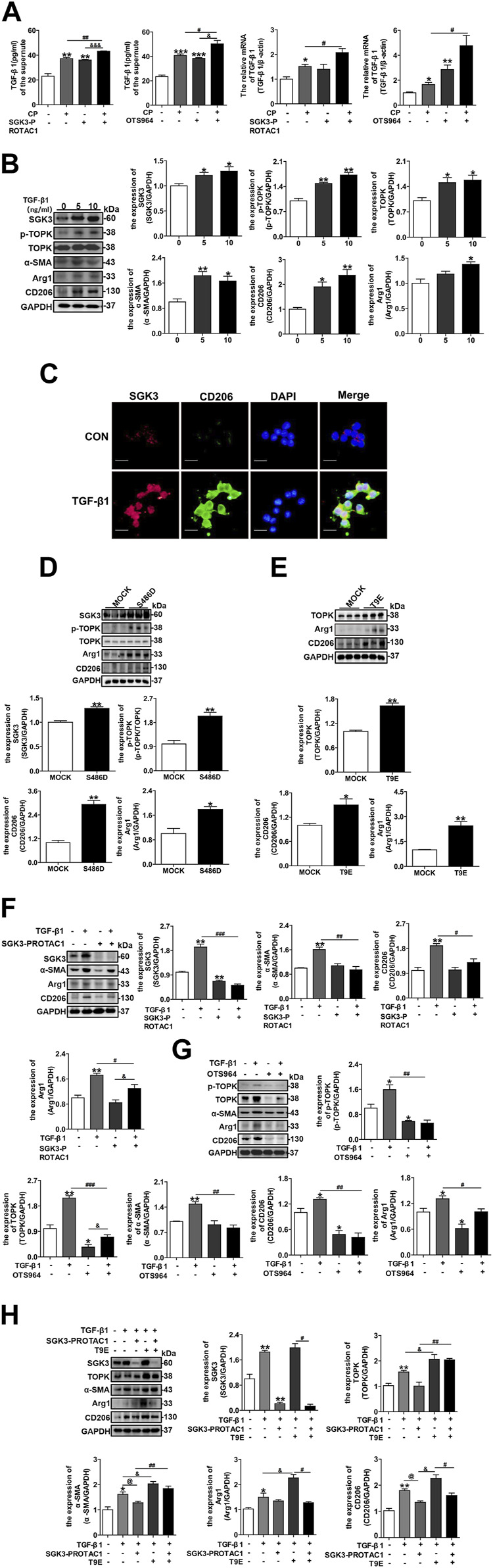
Inhibition of the SGK3/TOPK signaling pathway alleviates TGF-β1-induced CD206^**+**^ M2 macrophage transition. **(A)** The secretion and mRNA levels of TGF-β1 in NRK-52E cells treated with SGK3-PROTAC1 or OTS964 and CP for 24 h were measured by enzyme-linked immunoassay (ELISA) and RT-qPCR, respectively. *p < 0.05 and **p < 0.01 vs. the control group; ^**#**^ *p* < 0.05 and ^**##**^ *p* < 0.01 vs. the CP+/SGK3-PROTAC1 group; ^&&&^ *p* < 0.001 vs. the CP-/SGK3-PROTAC1 + group (n = 3); *p < 0.05 and ***p < 0.001 vs. the control group; ^**#**^ *p* < 0.05 vs. the CP+/OTS964 group; ^&&^ *p* < 0.01 vs. the CP-/OTS964+ group (n = 3). **(B)** RAW264.7 cells were exposed to different concentrations TGF-β1 (5 ng/mL and 10 ng/mL) for 24 h, and then, the expression of SGK3, p-TOPK, TOPK, CD206, Arg1, and α-SMA was measured by immunoblotting. *p < 0.05 and **p < 0.01 vs. the control group (n = 3). **(C)** Representative immunofluorescent images showed staining with specific antibodies to SGK3 (red), CD206 (green), and DAPI (blue) in RAW264.7 cells treated with TGF-β1 (10 ng/mL) for 24 h. Scale bar, 20 μm. **(D)** RAW264.7 cells were transiently transfected with SGK3-S486D plasmids for 48 h, immunoblotting analysis for SGK3, p-TOPK, TOPK, CD206, and Arg1 expression. *p < 0.05 and **p < 0.01 vs. the control group (n = 3). **(E)** RAW264.7 cells were transiently transfected with TOPK-T9E plasmids for 48 h; immunoblotting analysis for TOPK, CD206, and Arg1 expression. *p < 0.05 and **p < 0.01 vs. the control group (n = 3). **(F)** RAW264.7 cells were stimulated by TGF-β1 together with SGK3-PROTAC1 for 24 h, and the expression of SGK3, CD206, and Arg1 was measured by Western blotting. **p < 0.01 vs. the control group; ^**#**^ *p* < 0.05, ^**##**^ *p* < 0.01, and ^**###**^ *p* < 0.001 vs. the TGF-β1+/SGK3-PROTAC1 group; ^&^ *p* < 0.05 vs. TGF-β1+/SGK3-PROTAC1 + group (n = 3). **(G)** TGF-β1 and OTS964 were applied to RAW264.7 cells for 24 h, and the abundance of p-TOPK, TOPK, CD206, and Arg1 was measured by Western blotting. *p < 0.05 and **p < 0.01 vs. the control group; ^**##**^ *p* < 0.01 and ^**###**^ *p* < 0.001 vs. the TGF-β1+/OTS964 group; ^**&**^ *p* < 0.05 vs. the TGF-β1+/OTS964 + group (n = 3). **(H)** RAW264.7 cells were transiently transfected with TOPK-T9E plasmids for 48 h, and then SGK3-PROTAC1 and TGF-β1 were applied within the last 24 h. Immunoblotting analysis for the expression of SGK3, TOPK, α-SMA, CD206, and Arg1. *p < 0.05 and **p < 0.01 vs. the control group; ^**@**^ *p* < 0.05 vs. the TGF-β1+/SGK3-PROTAC1-/T9E group; ^&^ *p* < 0.05 vs. the TGF-β1+/SGK3-PRO- TAC1-/T9E + group; ^**#**^ *p* < 0.05 and ^**##**^ *p* < 0.01 vs. the TGF-β1+/SGK3-PROTAC1+/T9E + group (n = 3).

It is well known that profibrotic TECs secrete various profibrotic factors, including TGF-β1, and, thus, lead to neighborly macrophage CD206^**+**^ M2 phenotype transformation and even MMT (Meng et al., 2016b; Tang et al., 2019; Ruiz-Ortega et al., 2020; Schiessl, 2020). We further investigated whether the SGK3/TOPK axis participated in CP-induced TGF-β1 secretion in TECs and in TGF-β1 stimulated macrophage phenotype switching. As shown in Figure 5A, the inhibition of SGK3 or TOPK enhanced the secretion levels and mRNA expression of TGF-β1 in NRK-52E cells stimulated by CP. Additionally, the overexpression of SGK3 or TOPK in NRK-52E cells alleviated the secretion of TGF-β1 induced by cisplatin as shown in Supplementary Figures S4A,B. Subsequently, RAW264.7 cells were exposed to different concentrations of TGF-β1 for 24 h. Immunoblotting analysis revealed that the protein expression of SGK3 and p-TOPK was increased in a dose-dependent manner by TGF-β1 stimulation. Moreover, the expression of CD206, Arg1, and α-SMA, indicating M2 macrophage polarization and MMT, was upregulated (Figure 5B). In addition, immunofluorescence staining further confirmed that SGK3 was upregulated in TGF-β1-induced CD206^**+**^ M2 macrophages (Figure 5C). All of the aforementioned results supported that SGK3/TOPK signaling pathway was involved in TGF-β1-mediated CD206^+^ M2 macrophage transformation and MMT. We further transfected RAW264.7 cells and BMDMs with SGK3-S486D or TOPK-T9E plasmids for 48 h and detected that the protein abundance of CD206 and Arg1 increased by immunoblotting examination in the transfected groups as compared with the mock groups (Figures 5D,E; Supplementary Figures S5C,D). Furthermore, SGK3-PROTAC1 or OTS964 administration reversed TGF-β1-induced CD206^+^ M2 macrophage polarization and MMT (Figures 5F,G). Notably, TOPK activation reversed SGK3 inhibition diminishing TGF-β1-induced M2 macrophage transformation and MMT (Figure 5H). Thus, it is clear that SGK3/TOPK signaling pathway inhibition might be a protective factor against TGF-β1-induced CD206^**+**^M2 macrophage polarization and MMT.

### 3.6 Suppression of the SGK3/TOPK axis in macrophages hinders the profibrotic TEC-induced CD206^+^ M2 macrophage polarization and MMT

**FIGURE 6:**
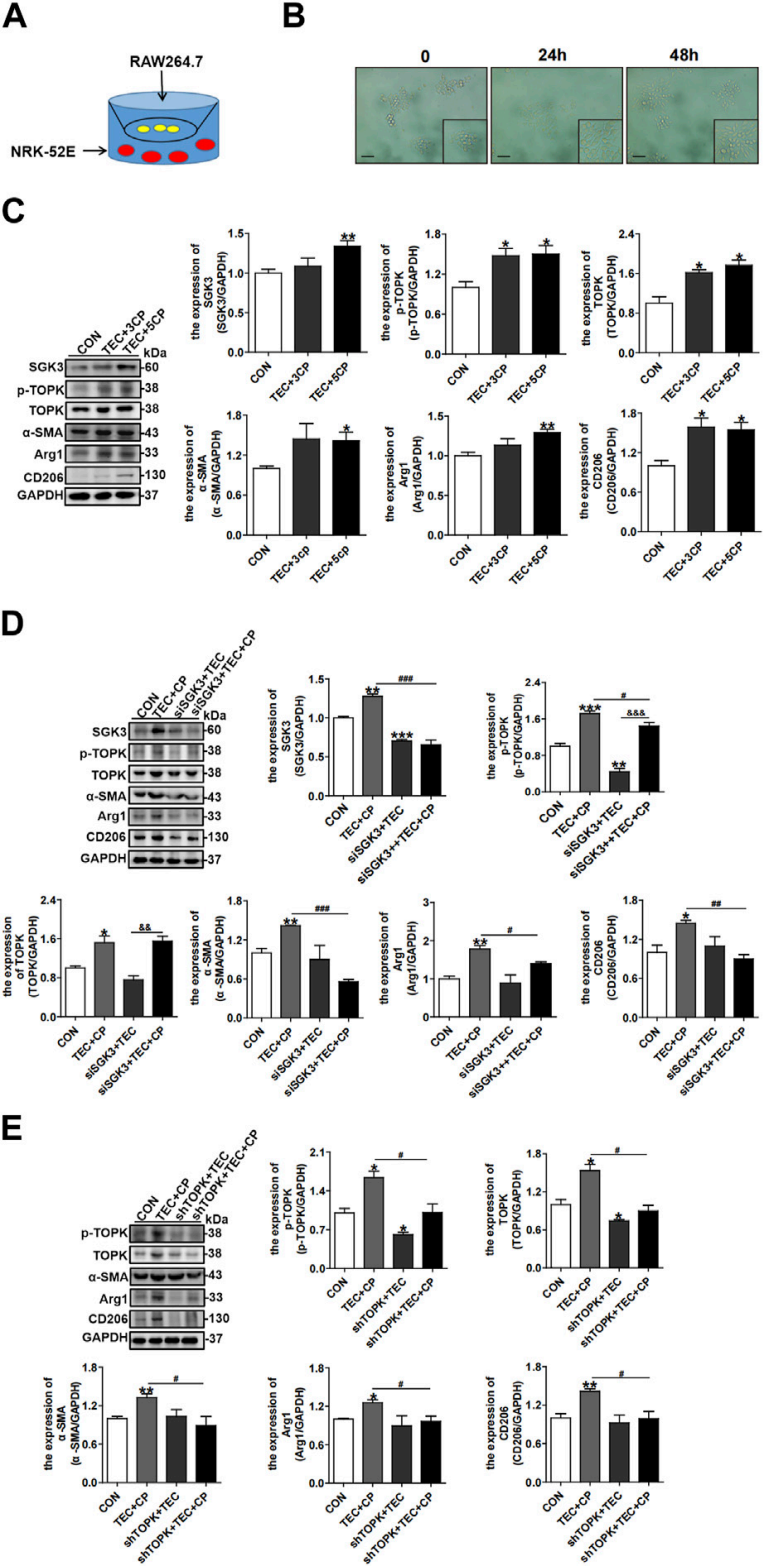
Suppression of the SGK3/TOPK axis in macrophages hinders the profibrotic TEC-induced CD206^**+**^ M2 macrophage polarization and MMT. **(A)** An *in vitro* co-culture system was used in which RAW264.7 cells were seeded in the top compartment, separated by a porous membrane from NRK-52E cells that were cultured in the bottom compartment. **(B)** Significant morphology alteration was observed in RAW264.7 cells, incubated with CP-induced profibrotic TECs for 24 h and 48 h, respectively. Scale bar, 20 µm. **(C)** The expression of SGK3, p-TOPK, TOPK, CD206, Arg1, and α-SMA was detected by Western blotting in RAW264.7 cells co-cultured with multiple times of CP (0, 3CP, and 5CP)-stimulated profibrotic TECs. *p < 0.05, **p < 0.01, and ***p < 0.001 vs. the control group (n = 3). **(D)** Immunoblotting analysis for SGK3, p-TOPK, TOPK, CD206, Arg1, and α-SMA in RAW264.7 cells that were pretreated with siSGK3 for 24 h and then co-cultured with profibrotic TECs for another 24 h. *p < 0.05, **p < 0.01, and ***p < 0.001 vs. the control group; ^**#**^ *p* < 0.05, ^**##**^ *p* < 0.01, and ^**###**^ *p* < 0.001 vs. the siSGK3-/TEC+/CP + group; ^&&^ *p* < 0.01 and ^&&&^ *p* < 0.001 vs. siSGK3+/TEC+/CP group (n = 3). **(E)** Immunoblotting analysis for p-TOPK, TOPK, CD206, Arg1, and α-SMA in RAW264.7 cells that were pretreated with shTOPK for 24 h and then co-cultured with profibrotic TECs for another 24 h. *p < 0.05 and **p < 0.01 vs. the control group; ^**#**^ *p* < 0.05 vs. the shTOPK-/TEC+/CP + group (n = 3).

To closely simulate the contact cells *in vivo*, we co-cultured NRK-52E and RAW264.7 cells *in vitro* through the Corning 0.4 μm polycarbonate membrane, as exhibited in Figure 6A. CP was used for inducing the profibrotic phenotype of TECs. The cellular morphology of RAW264.7 cells gradually approached that of the slender type of fibroblasts when being co-cultured with profibrotic NRK-52E cells for 24 h and 48 h (Figure 6B). Then, we treated NRK-52E cells seeded in the lower chamber with multiple times of cisplatin (0, 3CP, and 5CP). As expected, Western blotting analysis showed that the protein expression of CD206, Arg1, and α-SMA in co-cultured macrophages was upregulated, indicating CD206^+^ M2 macrophage polarization and MMT. Consistently, the abundance of SGK3 and p-TOPK in RAW264.7 cells increased significantly (Figure 6C). Next, RAW264.7 cells were pretreated with siSGK3 or shTOPK for 24 h and then co-cultured with profibrotic TECs for another 24 h. More interestingly, RAW264.7 cells pretreated with siSGK3 or shTOPK exhibited reduced macrophage CD206^+^ M2 transformation and MMT (Figures 6D,E). These data indicated that inactivating the SGK3/TOPK axis in macrophages could reduce macrophage CD206^+^ M2 transformation and MMT when co-cultured with profibrotic TECs.

### 3.7 Activation of the SGK3/TOPK signaling pathway in TECs attenuates TGF-β1-stimulated CD206^+^ M2 macrophage-induced EMT

**FIGURE 7:**
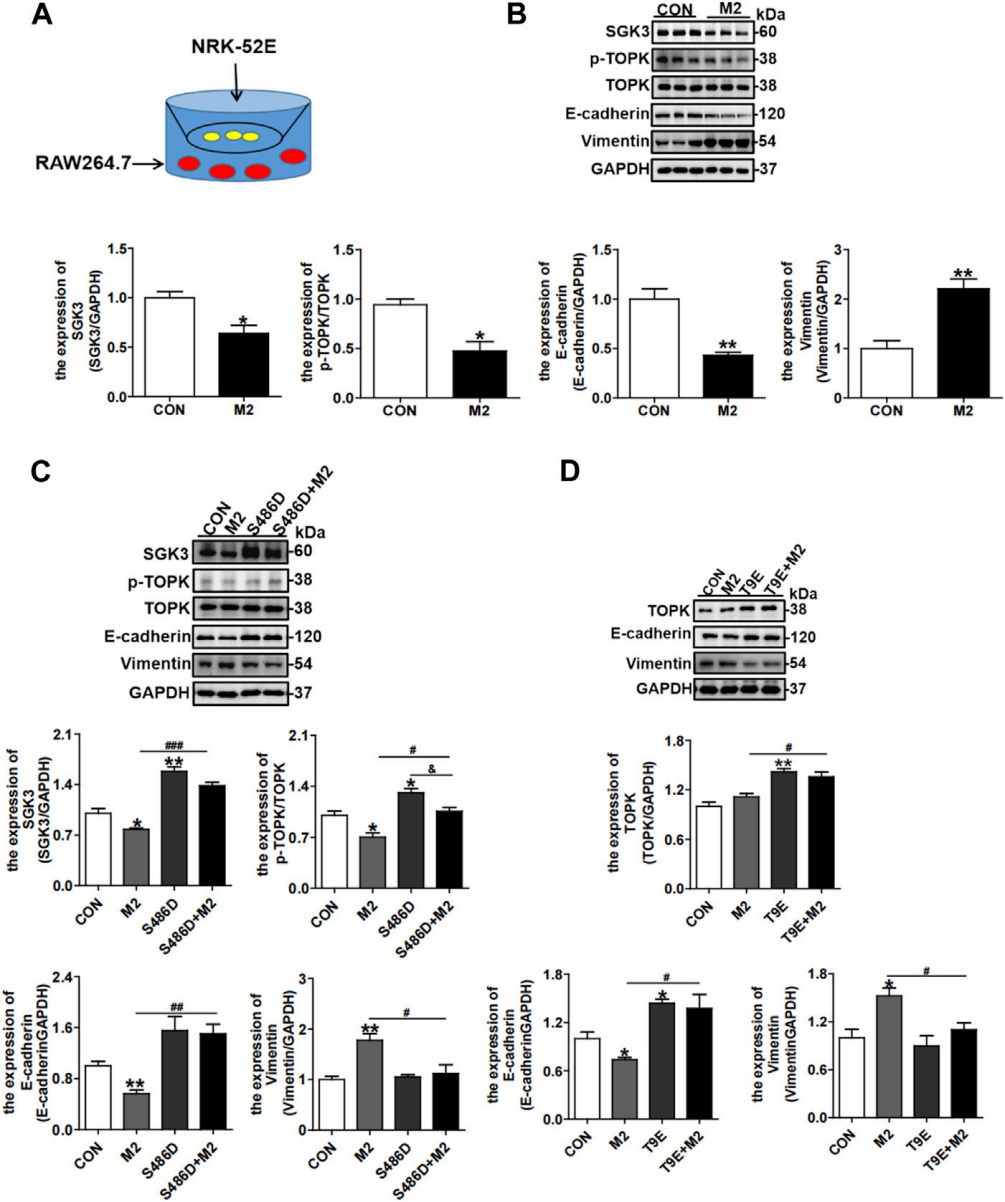
Activation of the SGK3/TOPK signaling pathway in TECs attenuates TGF-β1-stimulated CD206^**+**^ M2 macrophage-induced EMT. **(A)** An *in vitro* co-culture system was used in which NRK-52E cells were seeded in the top compartment, separated by a porous membrane from RAW264.7 cells that were cultured in the bottom compartment. **(B)** The expression of SGK3, p-TOPK, TOPK, E-cadherin, and vimentin was detected by Western blotting in NRK-52E cells co-cultured with TGF-β1-stimulated C206^**+**^ M2 macrophages. *p < 0.05 and **p < 0.01 vs. the control group (n = 3). **(C)** Immunoblotting analysis for SGK3, p-TOPK, TOPK, E-cadherin, and vimentin in NRK-52E cells that were pretreated with SGK3-S486D plasmid for 24 h and then co-cultured with TGF-β1-stimulated CD206^**+**^ M2 macrophages for another 24 h. *p < 0.05 and **p < 0.01 vs. the control group; ^#^ *p* < 0.05, ^##^ *p* < 0.01, and ^###^ *p* < 0.001 vs. S486D-/M2 + group; ^**&**^ *p* < 0.05 vs. the S486D+/M2 group (n = 3). **(D)** Immunoblotting analysis for TOPK, E-cadherin, and vimentin in NRK-52E cells that were pretreated with TOPK-T9E plasmids for 24 h and then co-cultured with TGF-β1-stimulated CD206^**+**^ M2 macrophages for another 24 h. *p < 0.05 and **p < 0.01 vs. the control group; ^**#**^ *p* < 0.05 vs. the T9E-/M2 + group (n = 3).

Considering that CD206^**+**^ M2 macrophages in the interstitial area could also indirectly affect TECs, we further co-cultured NRK-52E cells with RAW264.7 cells, as displayed in Figure 7A, and RAW264.7 cells were treated with TGF-β1 to elicit macrophage CD206^**+**^ M2 polarization. The NRK-52E cells co-cultured with CD206^+^ M2 macrophages exhibited significantly decreased expression of E-cadherin and increased expression of vimentin, representing EMT of TECs, accompanied by the downregulated expressions of SGK3 and p-TOPK (Figure 7B). However, the CD206^**+**^ M2 macrophage-triggered EMT of NRK-52E cells was mitigated by SGK3 or TOPK activation (Figures 7C,D). Collectively, these data inferred that profibrotic TECs facilitated profibrotic M2 macrophage phenotype polarization, which in turn aggravated EMT of TECs. Activating the SGK3/TOPK axis in TECs or suppressing it in macrophages could alleviate the profibrotic phenotype transition of TECs and reduce profibrotic M2 macrophage polarization.

## 4 Discussion

In the current study, we elucidated a novel SGK3/TOPK axis in AKI–CKD transition. This work provides the first demonstration that SGK3 and p-TOPK expression is downregulated in profibrotic TECs but enhanced in CD206^**+**^ M2 macrophages during the progression from AKI to CKD. Regarding the mechanism, SGK3 activation promoted TOPK phosphorylation at Thr9. In addition, the inhibition of the SGK3/TOPK axis in macrophages reversed the profibrotic TECs that triggered profibrotic M2 macrophage transformation. Moreover, the activation of the SGK3/TOPK axis in TECs is alleviated by TGF-β1-triggered CD206^**+**^ M2 macrophage-elicited EMT of TECs.

To our knowledge, this is the first study to reveal that SGK3 regulates the phosphorylation level of TOPK at Thr9. Actually, we found that SGK3 could not interact with TOPK, suggesting an indirect effect of SGK3 on p-TOPK. Accumulating reports have shown that CDK1/cyclinB1 phosphorylates TOPK at Thr9, which promotes G2/M cell cycle transition (Gaudet et al., 2000; Matsumoto et al., 2004). Recently, Hiraoka et al. demonstrated that SGK could directly phosphorylate CDC25 and Myt1 to trigger CDK1/cyclinB1 activation in starfish oocytes (Hiraoka et al., 2019). Thus, SGK3 might regulate p-TOPK by affecting CDC25 activity. As expected, our data indicated that p-TOPK was elevated by SGK3 wild-type plasmid transfection and weakened by SGK3 siRNA transfection. Although further studies are needed to delineate in detail the mechanism by which SGK3 regulates p-TOPK, our findings confirm the role of SGK3 on TOPK phosphorylation.

Profibrotic TECs characterized by EMT and profibrotic factor secretion have been implicated in the progression from AKI to CKD (Venkatachalam et al., 2015; Dan et al., 2019; Kurzhagen et al., 2020; Yamashita et al., 2020). As a serine/threonine protein kinase, our previous study demonstrated that the inhibition of TOPK regulated CP-induced G2/M arrest. However, the potential role of SGK3 in AKI and even in CKD has never been reported. Numerous reports indicated that SGK1, as an isoform sharing 80% amino acid sequence homology with SGK3, promotes the occurrence of EMT (Sayarlioglu et al., 2016; Abbruzzese et al., 2019). In contrast, our data demonstrated that SGK3 was downregulated in the AKI–CKD transition in TECs, implying different roles of SGK isoforms in different causes of AKI. In addition, abundant studies presented that TGF-β1 stimulation could upregulate the expression of SGK1 and TOPK in cancer cells to promote EMT (Abbruzzese et al., 2019; Lee et al., 2020). However, our previous report showed that TOPK depletion exacerbated the CP-induced G2/M phase cell cycle arrest in TECs (Zhang et al., 2022). Our current study revealed that SGK3 and TOPK inhibition in TECs facilitated CP-induced TGF-β1 synthesis and secretion in TECs. Thus, there might be negative feedback between the SGK3/TOPK signaling pathway and TGF-β1 in CP-elicited injury of TECs. However, the mechanism by which SGK3 and TOPK regulate TGF-β1 requires further exploration. One possibility is that the inhibition of SGK3 triggers the increased phosphorylation of JNK, which contributes to TGF-β1 expression (Hoang et al., 2016; Pirovano et al., 2017). In conclusion, the present findings infer that SGK3 and TOPK activation serves as a protective effect in ameliorating the profibrotic phenotype of TECs.

Following injury to the kidney, macrophages exhibit a proinflammatory phenotype (M1) at early stages, but at later stages, M2 macrophages exhibit anti-inflammatory features and are involved in renal repair and fibrosis (Spiller et al., 2015). TOPK is known to be involved in solar UV light-induced inflammation, and overexpression of TOPK promoted M2 macrophage polarization (Li et al., 2011; Han et al., 2018). Additionally, it has been reported that β-catenin, as a downstream of SGK3 and TOPK, can suppress M2 macrophage polarization when ablated (Brown-Clay et al., 2015; Feng et al., 2018; Liu et al., 2018). In line with these reports, we found that the activation or inhibition of the SGK3/TOPK axis could reduce CP-induced M1 macrophage polarization or alleviate CD206^+^ M2 macrophage polarization, respectively, suggesting that SGK3/TOPK signaling pathway eliciting macrophage polarization might regulate the development of CKD. However, SGK3/TOPK-mediated macrophage transformation through β-catenin remains to be further determined.

The relationship between TECs SGK3/TOPK axis inhibition and renal macrophage SGK3/TOPK signaling pathway activation has not been elucidated before. We first showed that in the progression of AKI to CKD, interstitial CD206^**+**^ M2 macrophage polarization was associated with SGK3/TOPK axis-inhibited tubules, and MMT was markedly upregulated following AKI. As reported, TGF-β1 is one of the key factors mediating the crosstalk between injured tubular cells and macrophages (Venkatachalam et al., 2015; Zhu et al., 2017). In this respect, we hypothesized that TGF-β1 may mediate SGK3/TOPK axis activity in macrophages, thus affecting macrophage M2 polarization and MMT. Interestingly, we provided direct *in vitro* evidence that the inhibition of the SGK3/TOPK axis in macrophages alleviated TGF-β1-triggered CD206^**+**^ M2 macrophage polarization and MMT. Therefore, it may be concluded that SGK3/TOPK signaling pathway activation mediated macrophage CD206^**+**^ M2 polarization and, thus, contributed to AKI–CKD transition. As inferred previously, the persistence of abnormal CD206^**+**^ M2 macrophages promotes kidney fibrosis not only through MMT but also disorganizing physiological of peripheral TECs (Tang et al., 2019; Wang et al., 2022). Here, our results showed that the CD206^**+**^ M2 macrophages triggered the EMT of TECs, but it could be alleviated by SGK3/TOPK signaling pathway activation in TECs. These findings shed new light on the transition from AKI to CKD, although whether targeted intervention with the SGK3/TOPK pathway in TECs and macrophages could serve as a novel therapeutic to ameliorate the AKI–CKD transition still needs to be further explored.

## 5 Conclusion

**FIGURE 8:**
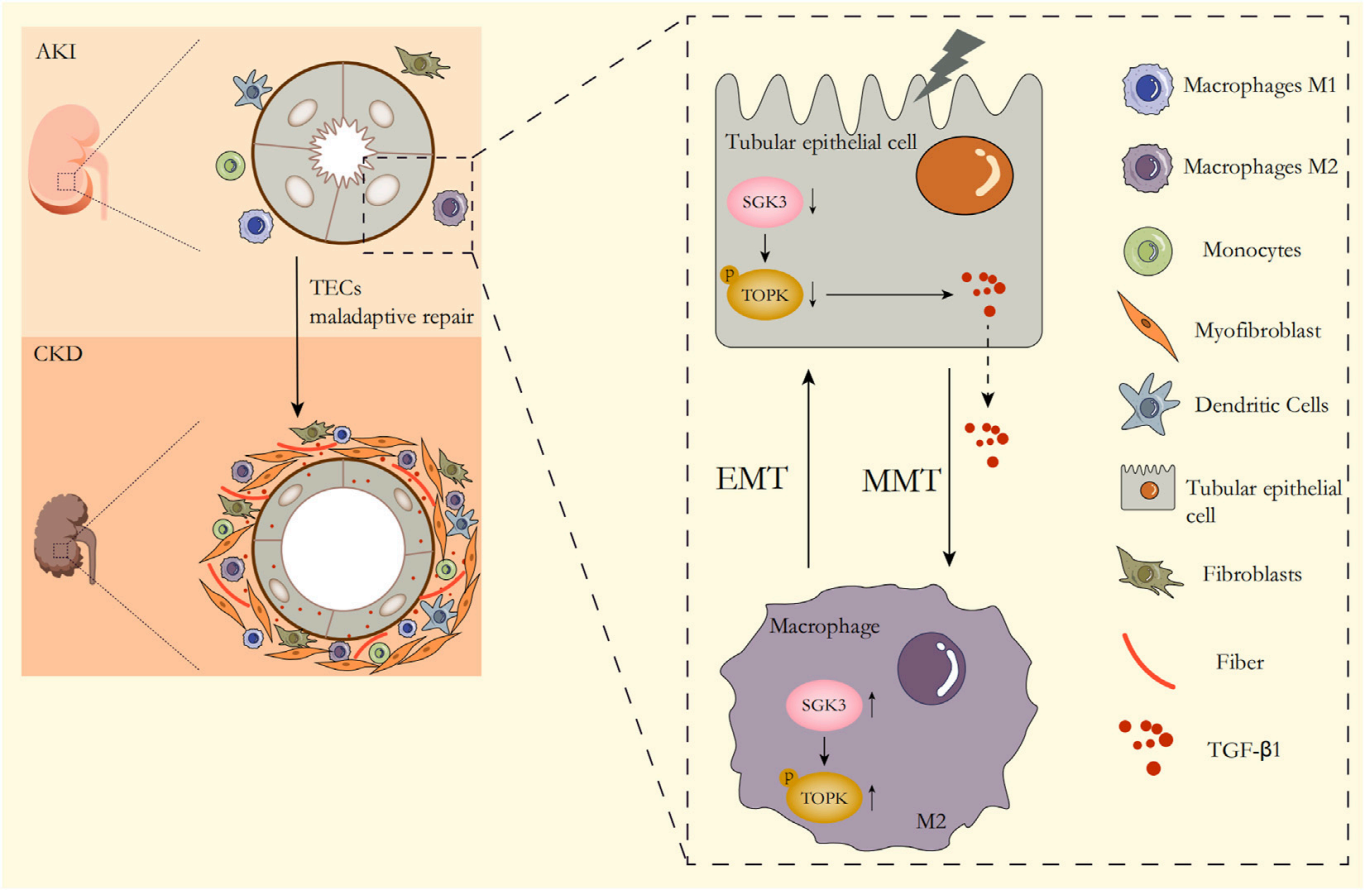
Summary schematics of the role of the SGK3/TOPK axis in the AKI–CKD transition. With the occurrence of AKI, the expression of SGK3 in TECs decreases, which downregulates TOPK phosphorylation at Thr9 and then aggravates EMT of TECs and the secretion of TGF-β1. TGF-β1 secreted from TECs upregulates the activation of the SGK3/TOPK signaling pathway in macrophages, which exacerbates MMT of macrophages. M2 macrophages that undergo MMT transformation in turn promote EMT of TECs, which together promote AKI–CKD transition.

In summary, following injury to the kidney, SGK3 inactivation exacerbated the EMT of TECs and magnified the secretion of TGF-β1 through downregulating the phosphorylation level of TOPK. Moreover, TGF-β1 from profibrotic TECs triggered the upregulation of the SGK3/TOPK signaling pathway in macrophages to promote CD206^**+**^ M2 transformation and MMT, while TGF-β1 stimulated CD206^**+**^ M2 macrophages, in turn, exacerbating the EMT of TECs, which together accelerated the transition from AKI to CKD (Figure 8).

## Figures and Tables

**Table:** 

**AKI**	Acute kidney injury
**CKD**	Chronic kidney disease
**AKI-CKD**	AKI-to-CKD transition
**EMT**	Epithelial-to-mesenchymal transition
**MMT**	Macrophage-to-myofibroblast transition
**TECs**	Tubular epithelial cells
**UUO**	Unilateral ureteral obstruction
**5/6 Nx**	5/6 nephrectomy
**CP**	Cisplatin
**BUN**	Blood urea nitrogen
**Scr**	Serum creatinine
**IHC**	Immunohistochemical
**HE**	Hematoxylin and eosin
**SGK3**	Serum and glucocorticoid-inducible kinase
**TOPK**	T-LAK-cell-originated protein kinase
**NGAL**	Neutrophil gelatinase-associated lipocalin
**α-SMA**	α-smooth muscle actin

## Data Availability

The original contributions presented in the study are included in the article/Supplementary Materials; further inquiries can be directed to the corresponding author.
