# Supplementary material for: The role of the SGK3/TOPK signaling pathway in the transition from acute kidney injury to chronic kidney disease
Source: Front Pharmacol. 2023 Jun 8;14:1169054. doi: 10.3389/fphar.2023.1169054 (PMC10285316; doi:10.3389/fphar.2023.1169054)

The role of SGK3/TOPK signaling pathway in the transition from acute kidney injury to chronic kidney disease

Original data

Figure 1C MASSON

CON

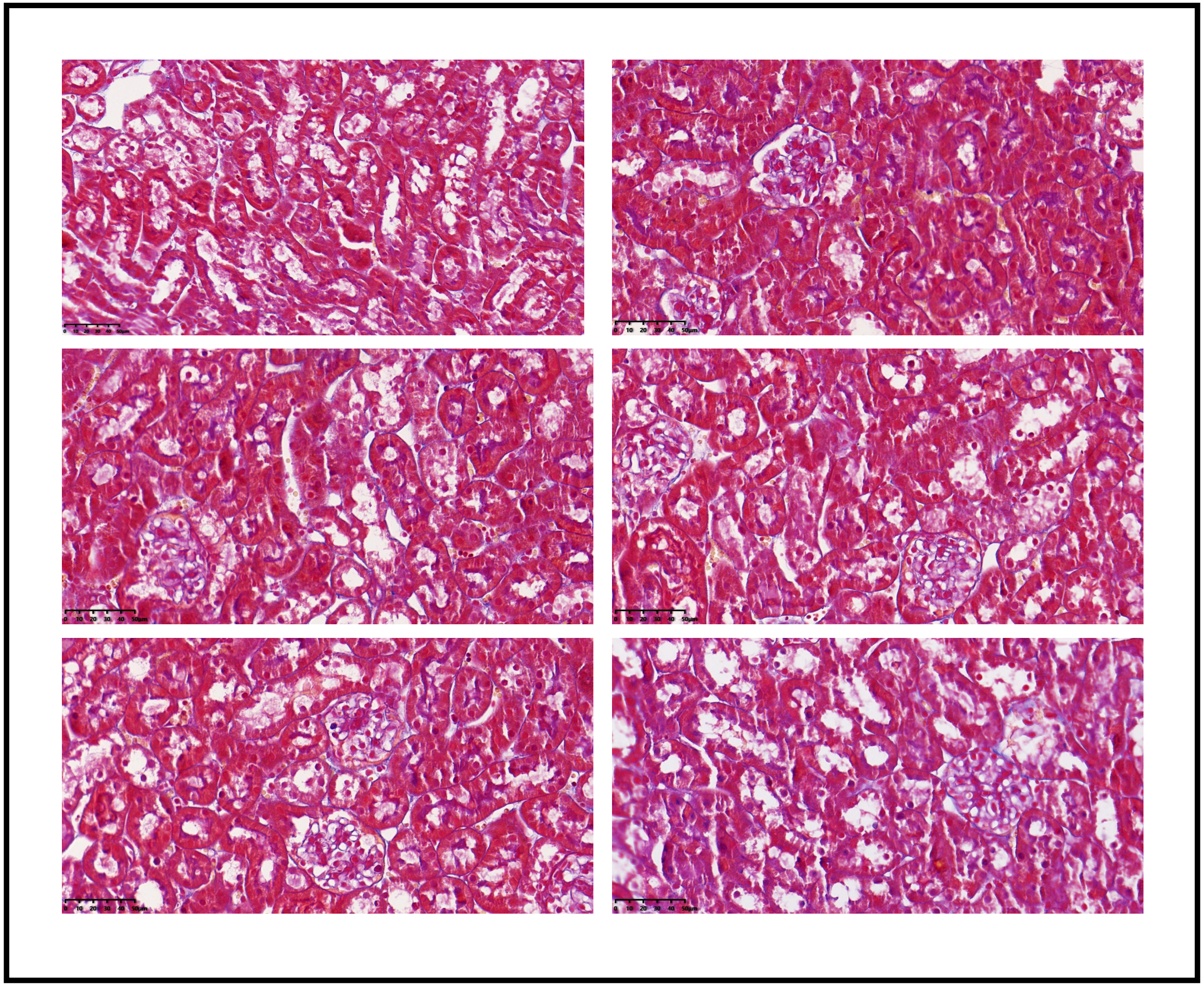

Figure 1C MASSON

2CP

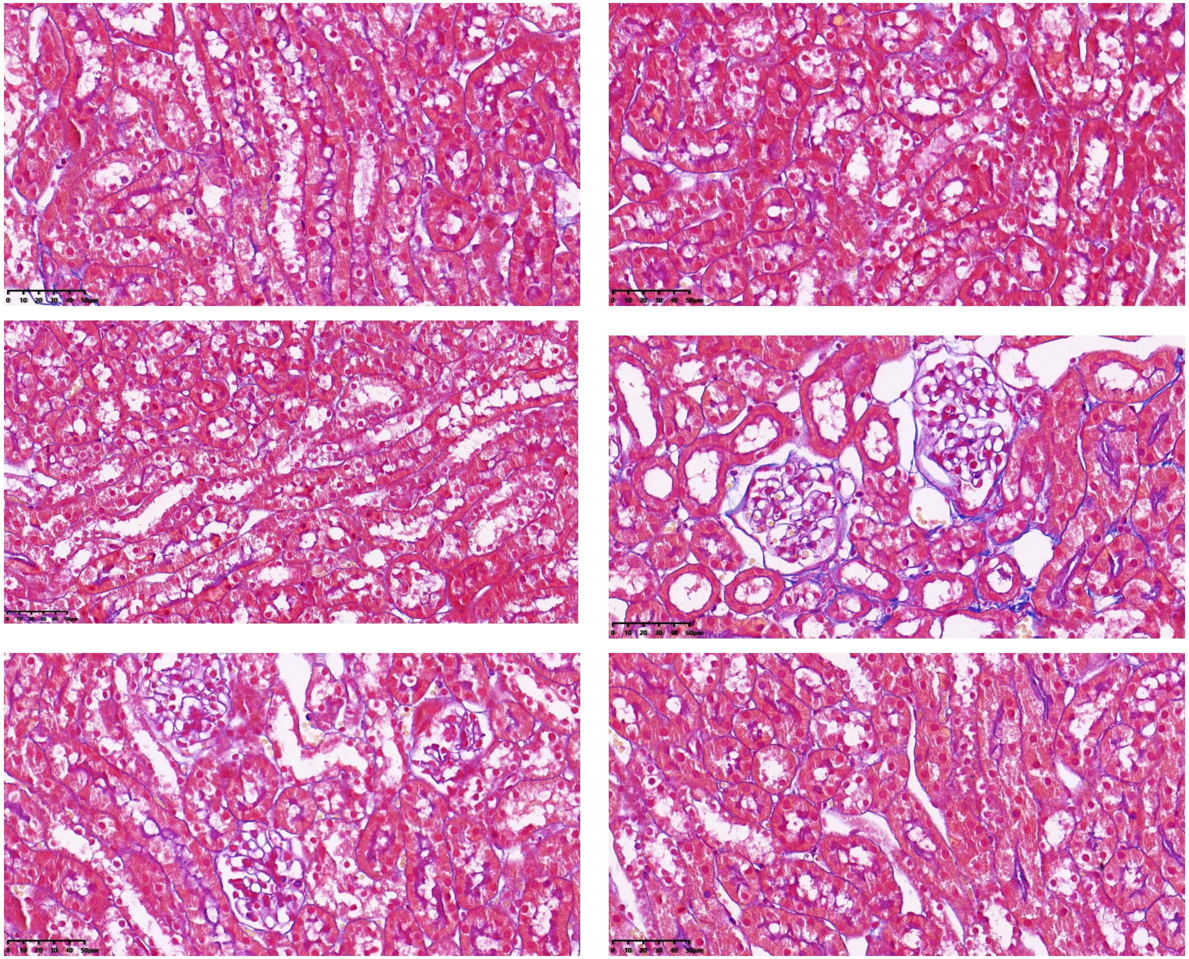

Figure 1C MASSON

3CP

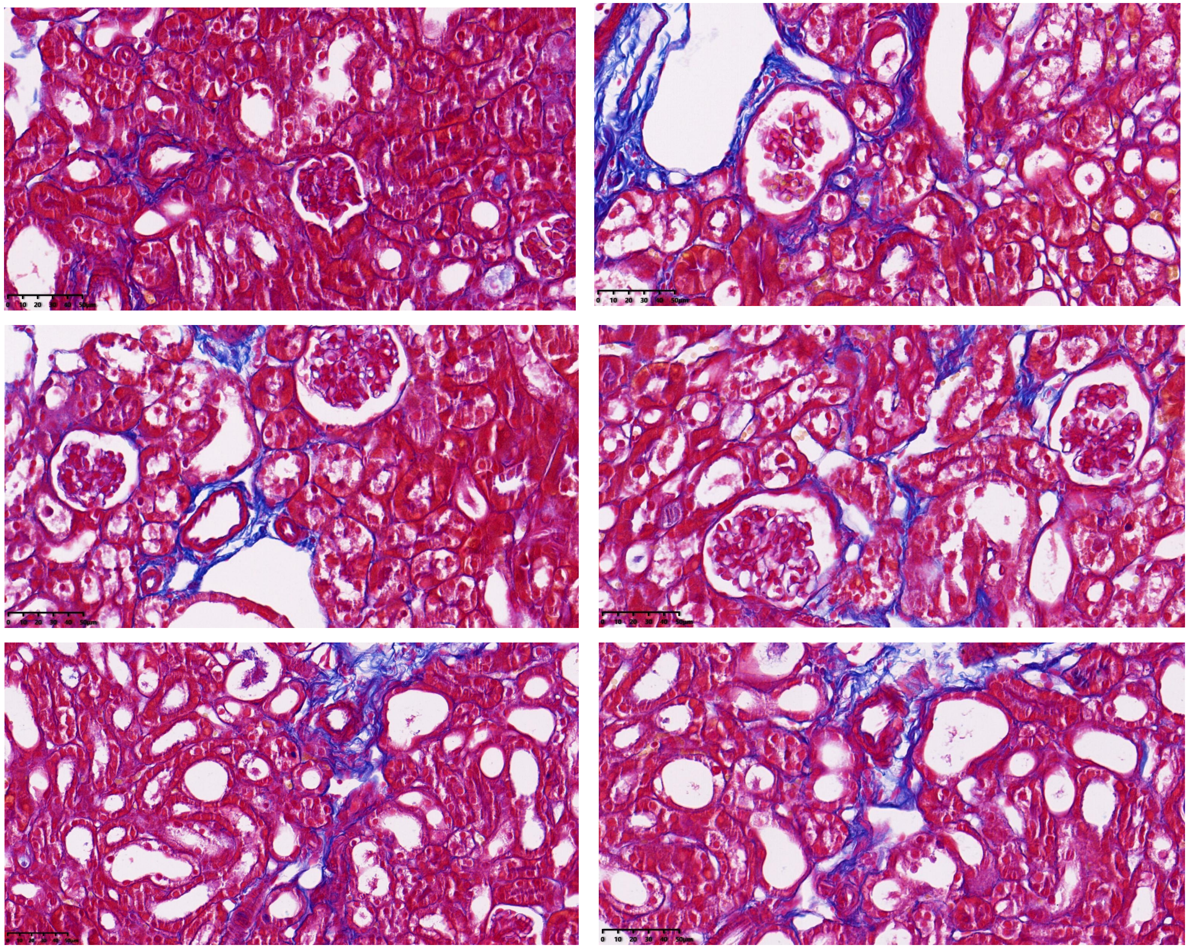

Figure 1C MASSON

4CP

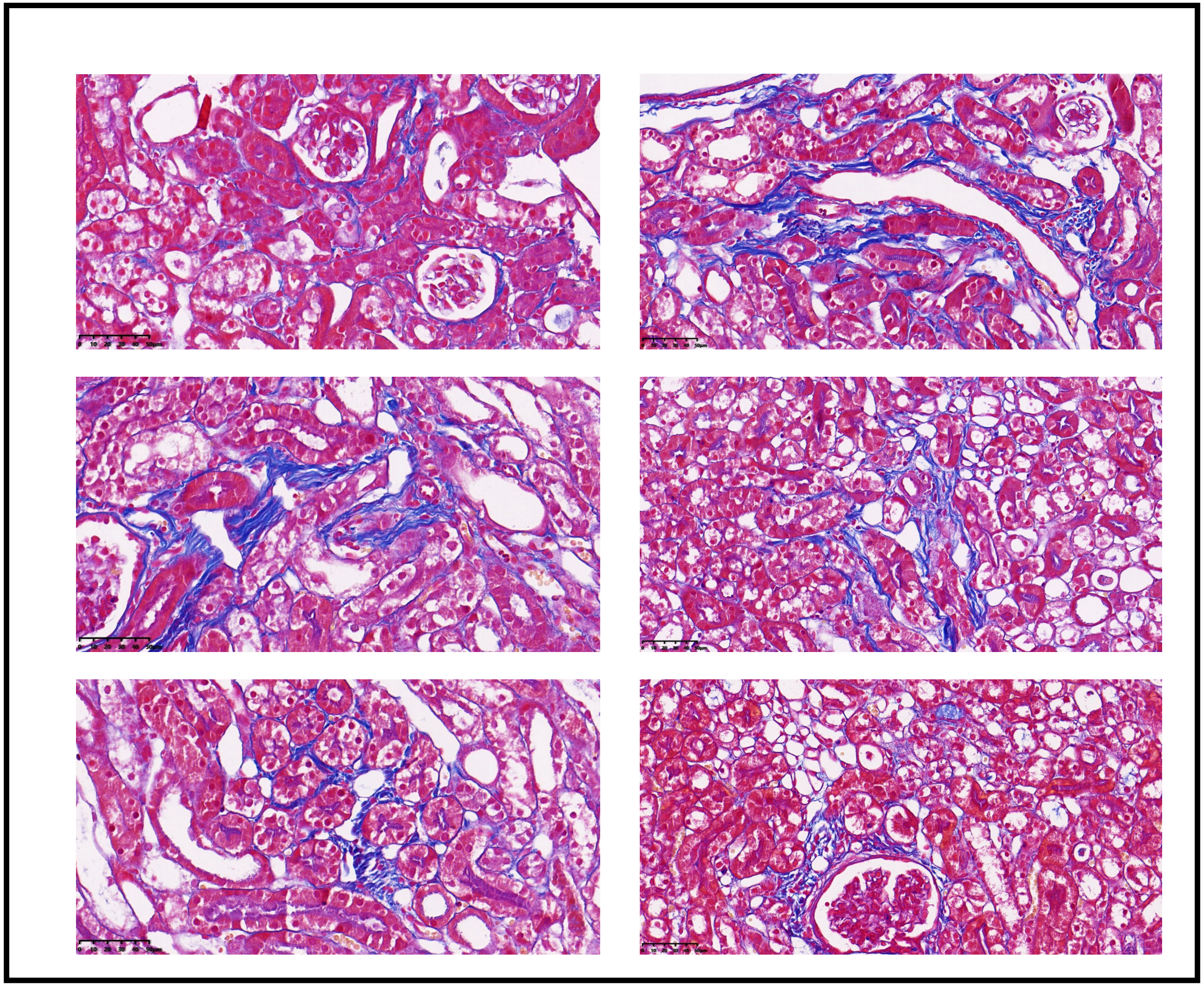

Figure 1E

IHC α-SMA

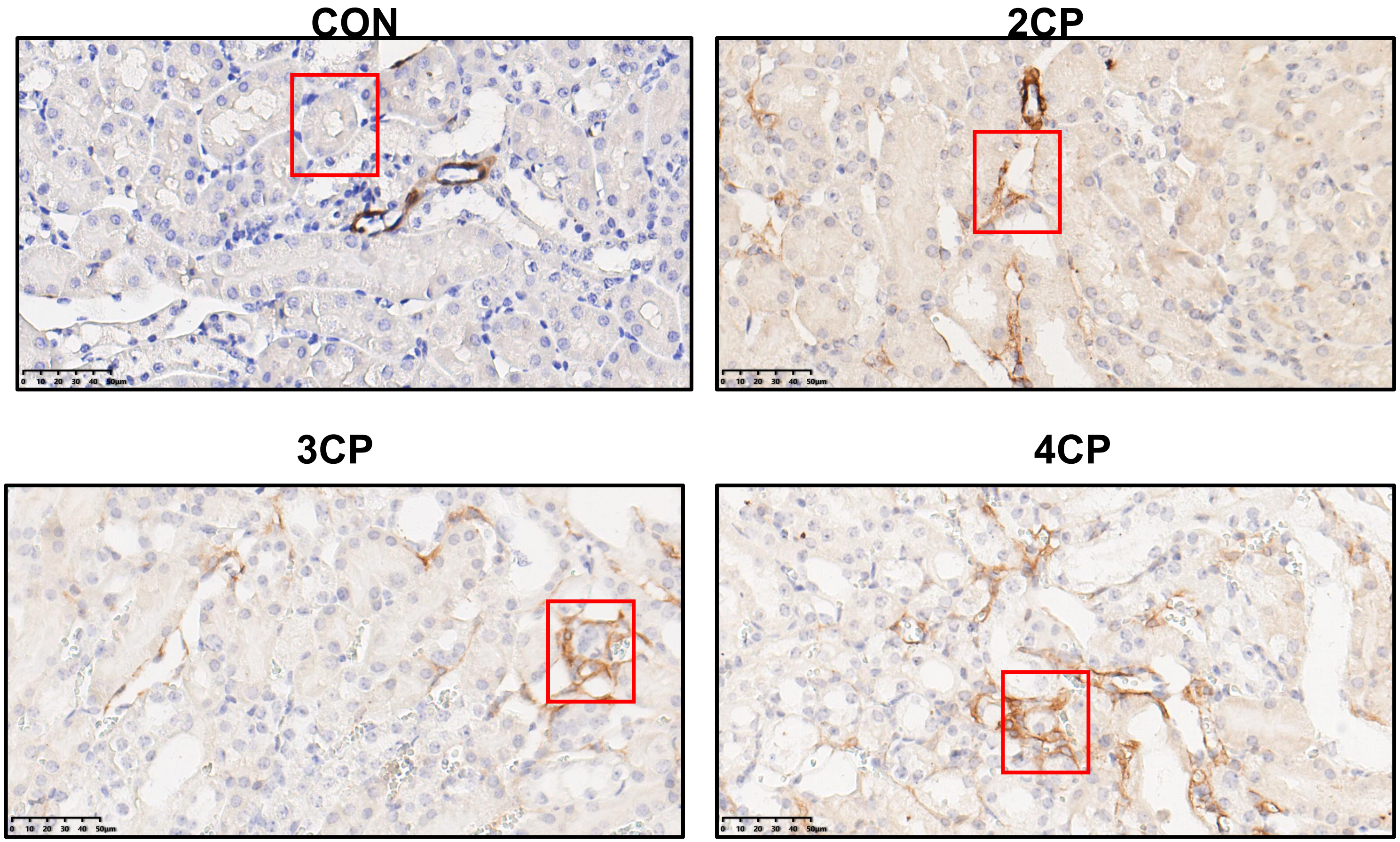

Figure 2H

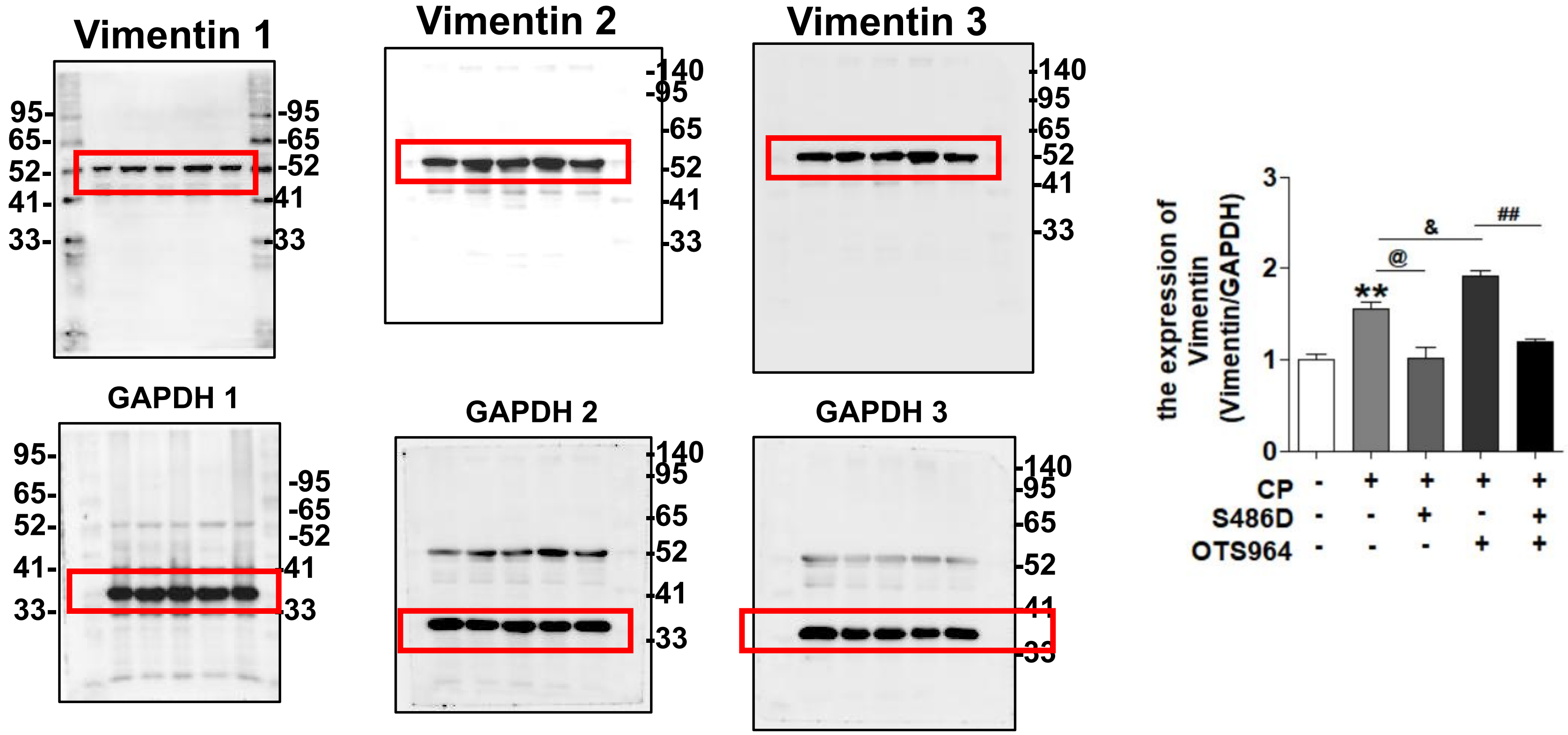

	VIMENTIN	GAPDH	VIMENTIN/GAPDH	control group was normalized to 1
CON 1	7946.376	13609.861	0.583869005	0.871213983
CON 2	14003.962	19110.74	0.732779683	1.093409482
CON 3	15003.912	21622.983	0.69388724	1.035376534
CP 1	13155.225	13700.518	0.960199096	1.432750963
CP 2	16308.397	15675.912	1.040347573	1.552343668
CP 3	15981.154	14086.841	1.134473939	1.692793334
CP+S486D 1	9511.083	16506.447	0.576204134	0.859776927
CP+S486D 2	11836.569	18383.468	0.643870297	0.960744279
CP+S486D 3	14476.347	17463.74	0.828937387	1.236890187
CP+OTS 1	16890.983	14059.669	1.201378425	1.792624157
CP+OTS 2	19206.811	14551.083	1.31995749	1.96956066
CP+OTS 3	17313.447	13043.669	1.32734486	1.980583645
CP+S486D+OTS 1	11528.962	13987.083	0.824257781	1.229907563
CP+S486D+OTS 2	13950.64	17195.811	0.811281306	1.210544851
CP+S486D+OTS 3	12619.69	16371.154	0.770849141	1.150214423

Figure 3A

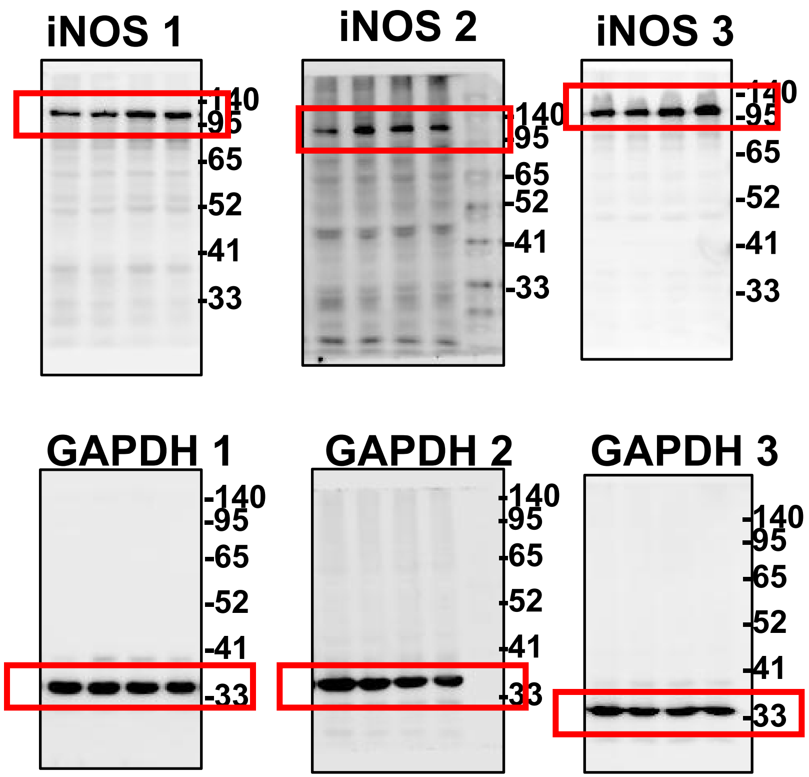

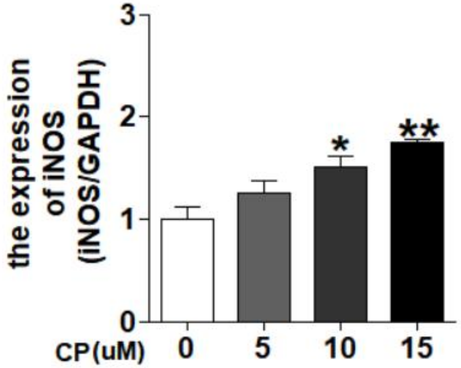

	iNOS	GAPDH	iNOS/GAPDH	control group was normalized to 1
0	12332.054	17038.841	0.723761317	1.226522042
0	9710.447	19202.74	0.505680283	0.85695104
0	9354.719	17296.79	0.540835554	0.916526916
5	11340.447	16381.912	0.692254176	1.173128469
5	12224.69	13878.983	0.880805892	1.492657615
5	9213.79	13985.912	0.658790789	1.116419743
10	15868.205	15610.326	1.01651977	1.722645125
10	11463.569	13304.79	0.861612171	1.460130978
10	12090.497	15113.69	0.799969895	1.355668901
15	13657.276	13404.891	1.018827829	1.726556477
15	10097.861	10093.74	1.000408273	1.695341777
15	13556.497	12693.74	1.067967124	1.809830378

Figure 3C

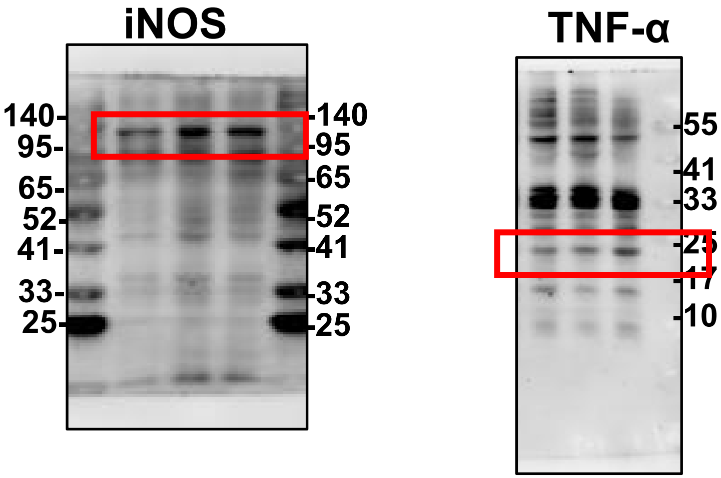

Figure 5E

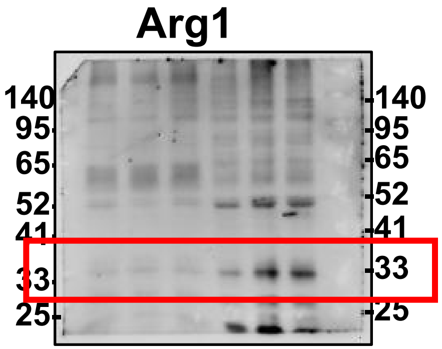

Supplement: Supplementary file 1 [file DataSheet2.PDF]
