# Supplementary figures and images for: The role of the SGK3/TOPK signaling pathway in the transition from acute kidney injury to chronic kidney disease
Source: Front Pharmacol. 2023 Jun 8;14:1169054. doi: 10.3389/fphar.2023.1169054 (PMC10285316; doi:10.3389/fphar.2023.1169054)

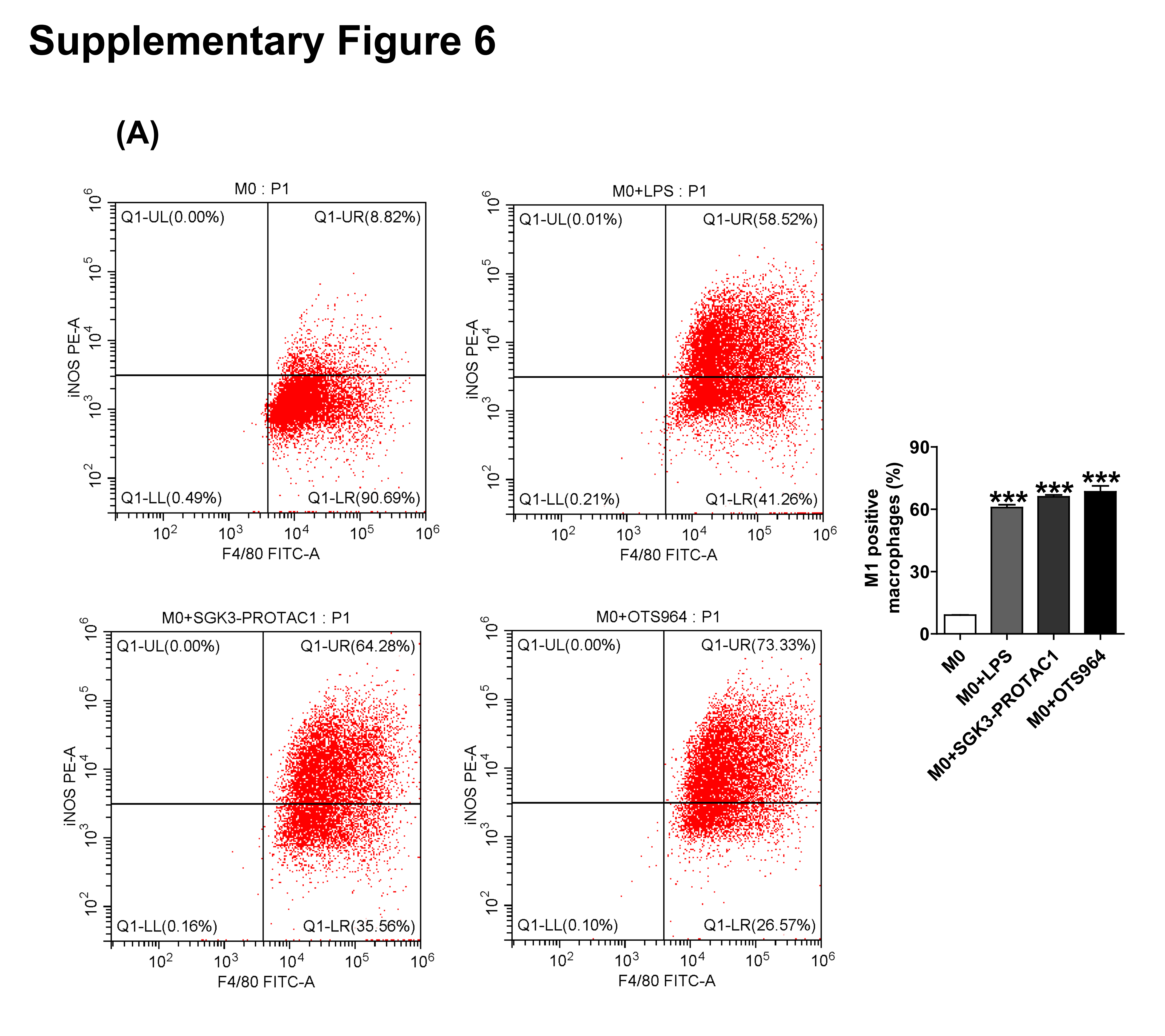

Supplement: Supplementary file 4 [file Image6.TIF]

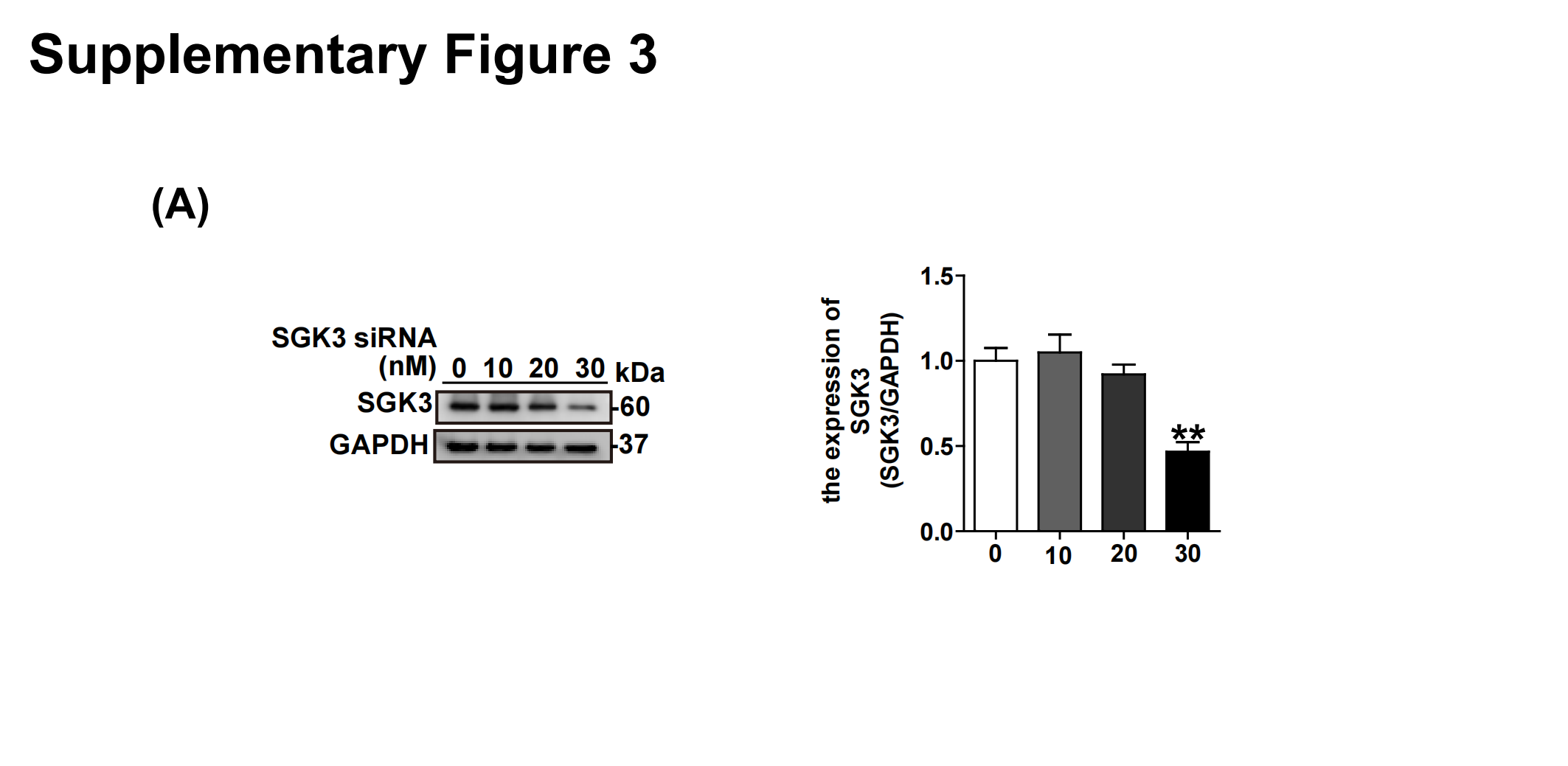

Supplement: Supplementary file 5 [file Image3.TIF]

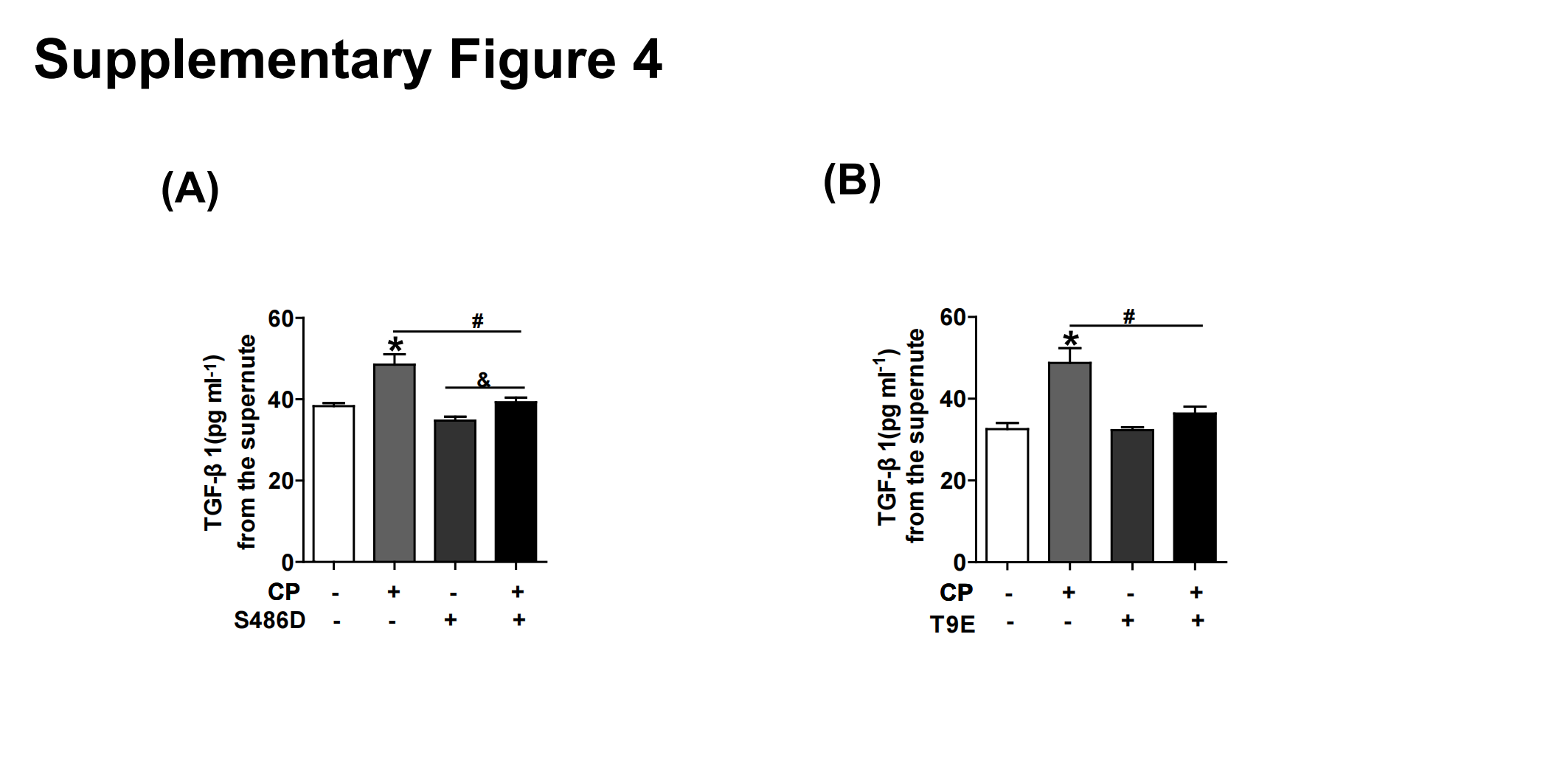

Supplement: Supplementary file 6 [file Image4.TIF]

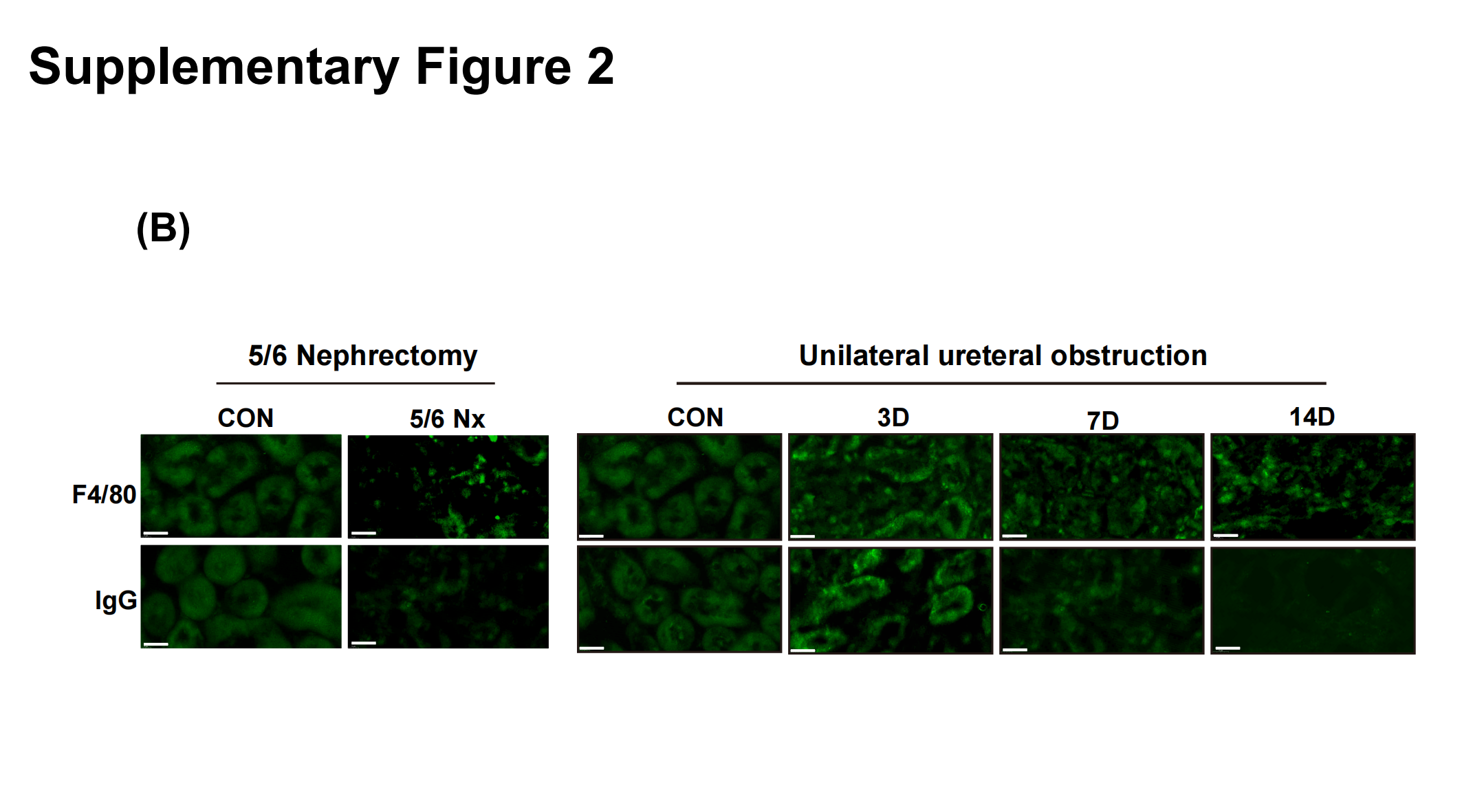

Supplement: Supplementary file 7 [file Image2.TIF]

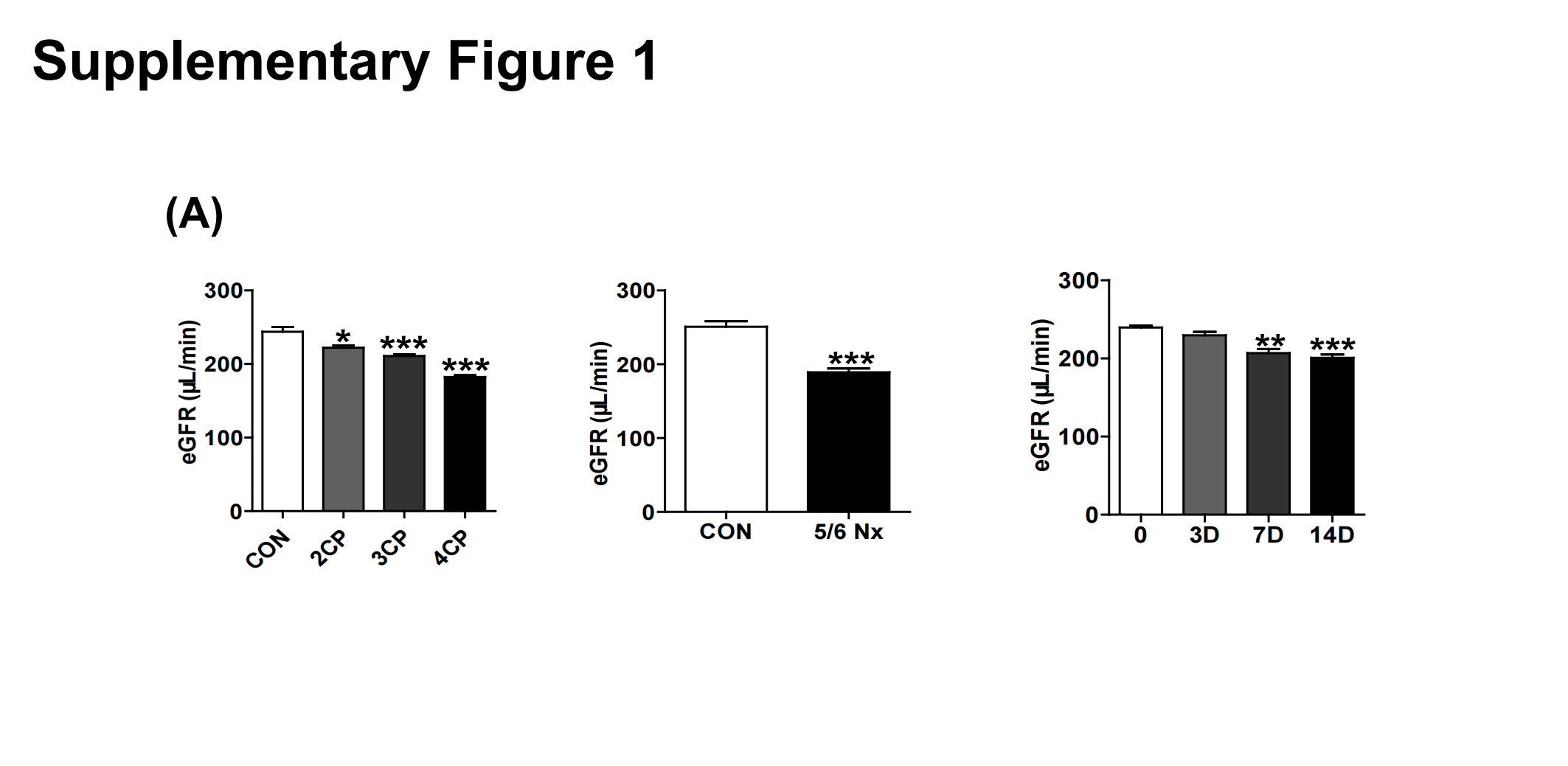

Supplement: Supplementary file 9 [file Image1.TIF]

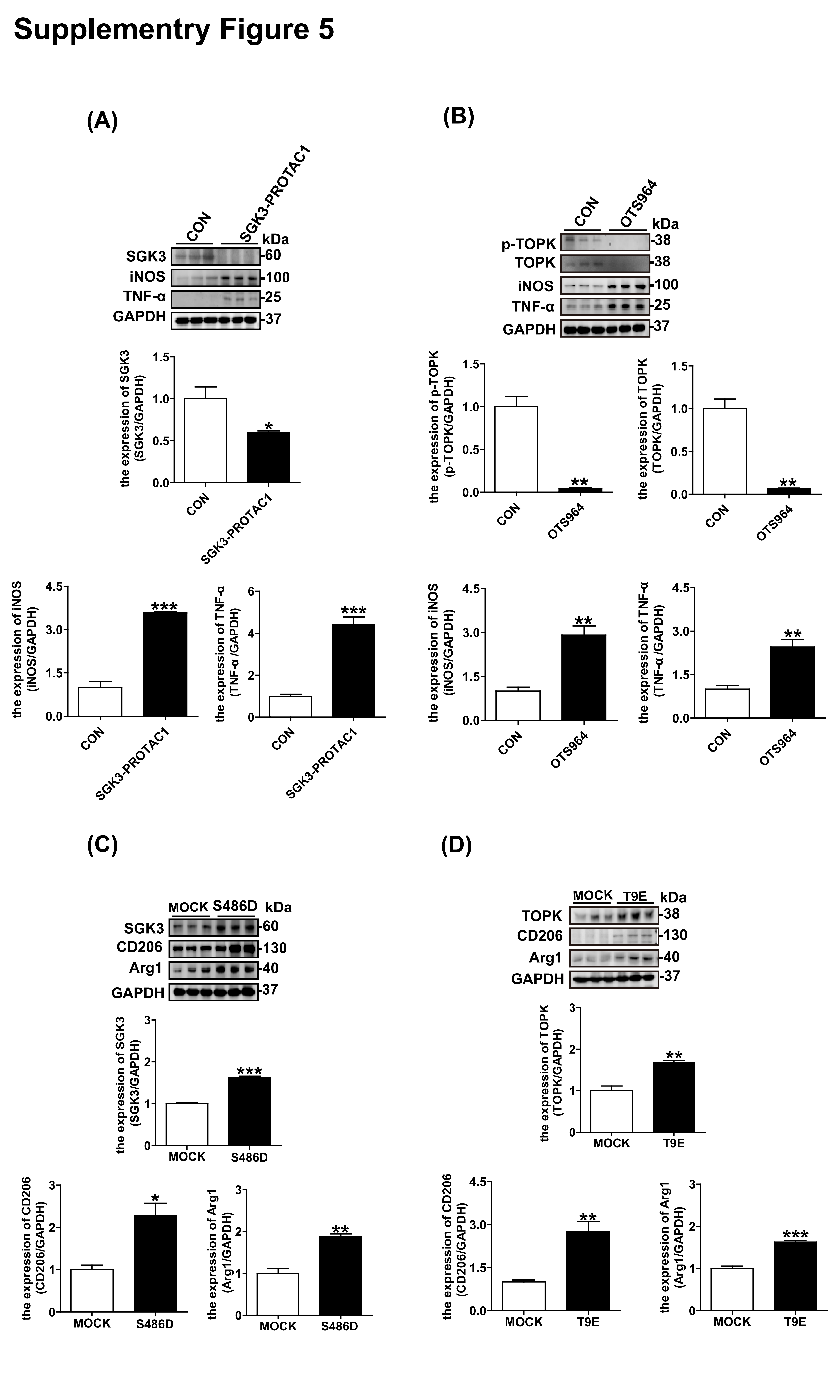

Supplement: Supplementary file 12 [file Image5.TIF]
