# Supplementary material for: The role of the SGK3/TOPK signaling pathway in the transition from acute kidney injury to chronic kidney disease
Source: Front Pharmacol. 2023 Jun 8;14:1169054. doi: 10.3389/fphar.2023.1169054 (PMC10285316; doi:10.3389/fphar.2023.1169054)

The role of SGK3/TOPK signaling pathway in the transition from acute kidney injury to chronic kidney disease

Supplementary data

Supplementary Figure 3

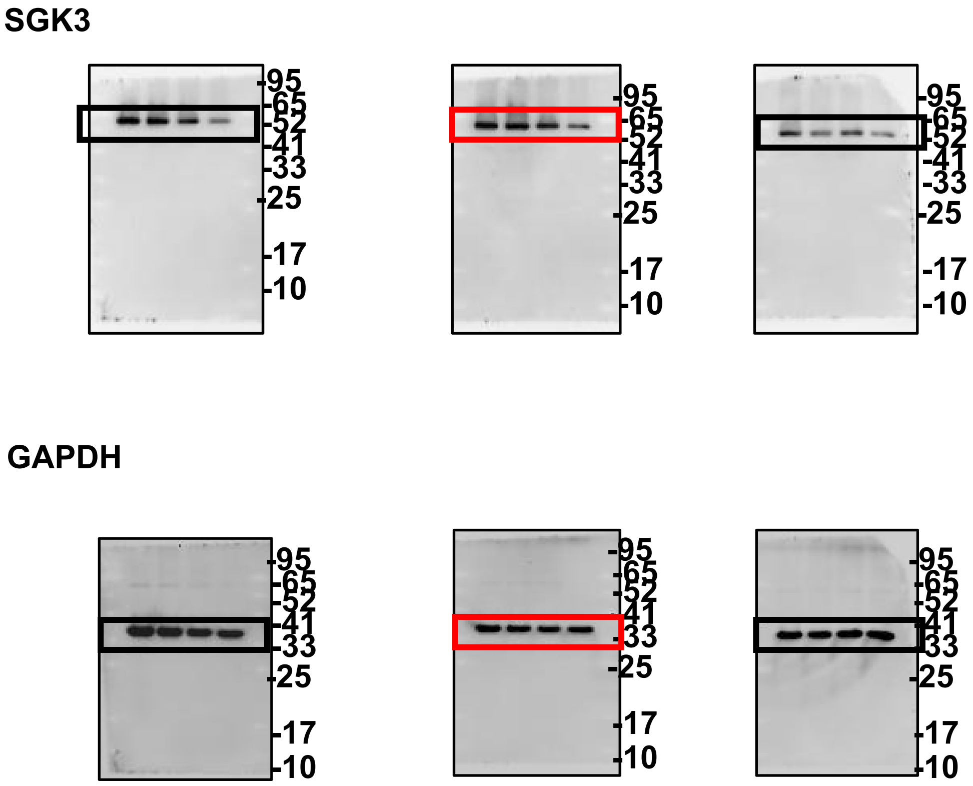

Supplementary Figure 5A

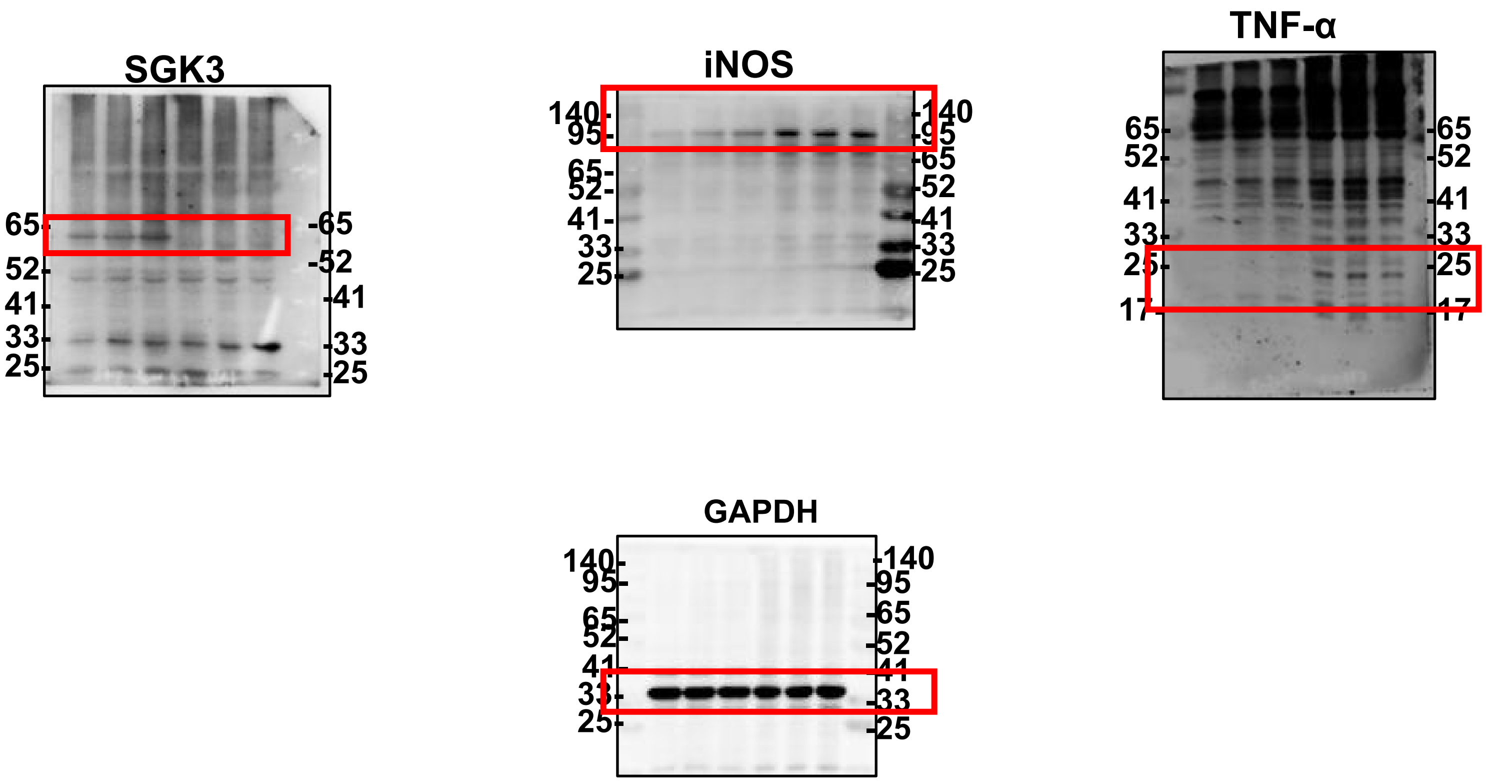

Supplementary Figure 5B

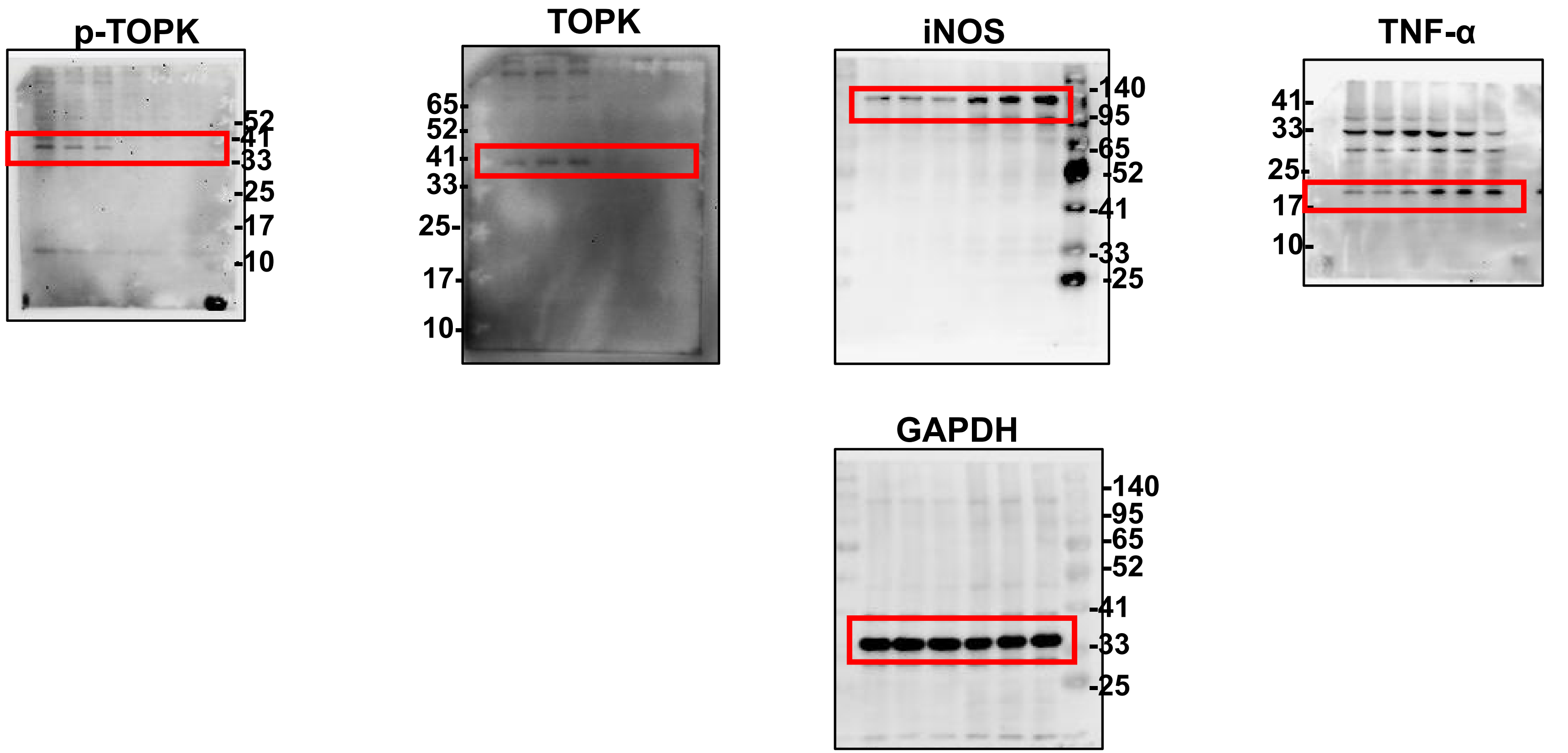

Supplementary Figure 5C

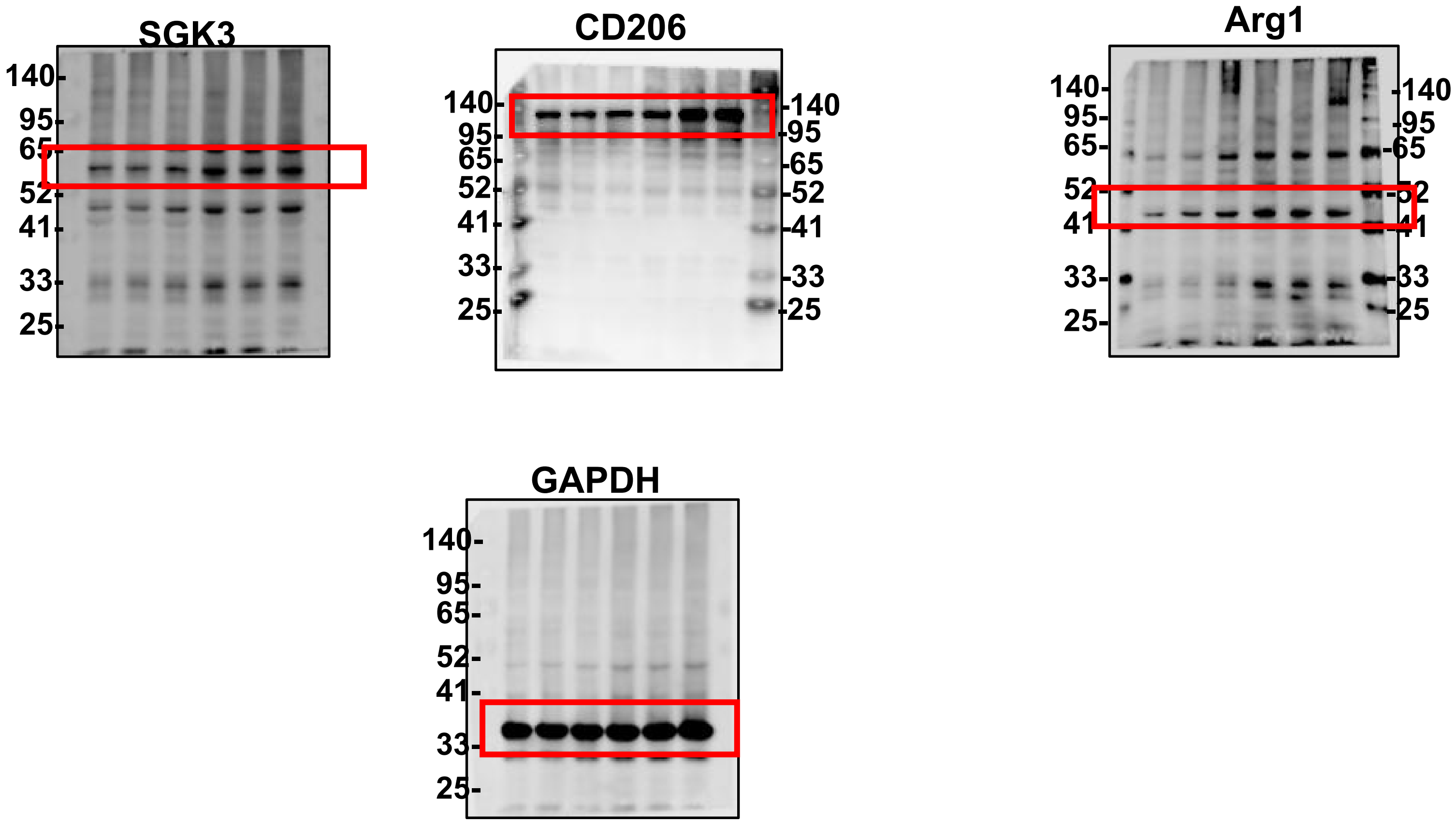

Supplementary Figure 5D

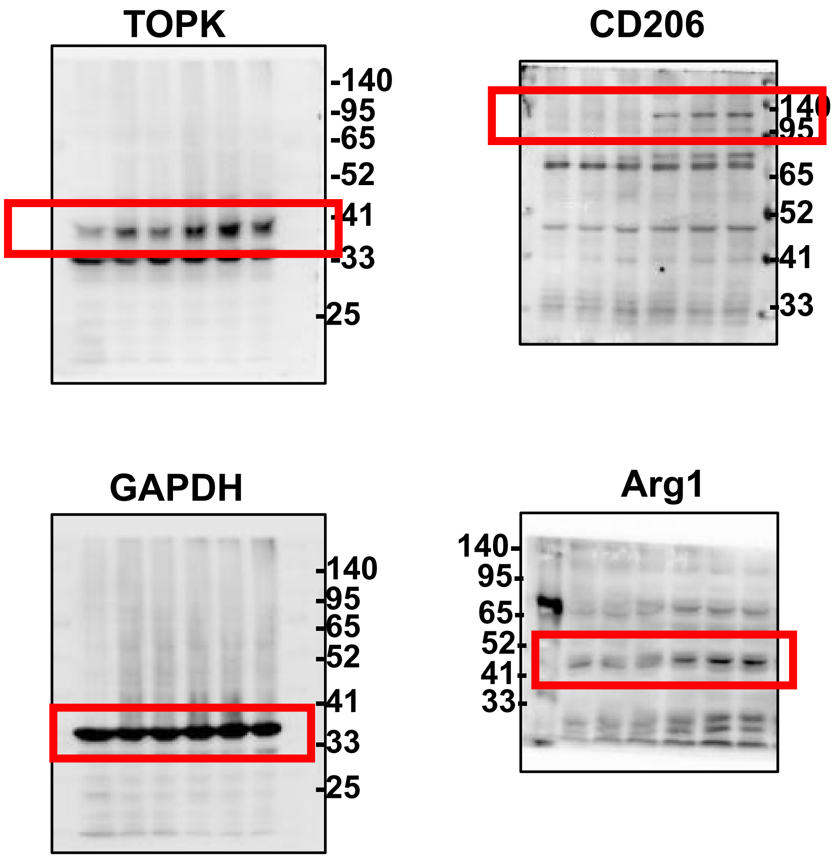

Supplement: Supplementary file 10 [file DataSheet3.PDF]
