# Supplementary material for: The role of the SGK3/TOPK signaling pathway in the transition from acute kidney injury to chronic kidney disease
Source: Front Pharmacol. 2023 Jun 8;14:1169054. doi: 10.3389/fphar.2023.1169054 (PMC10285316; doi:10.3389/fphar.2023.1169054)
Supplement: Supplementary file 11 [file DataSheet1.PDF]

The role of SGK3/TOPK signaling pathway in the progress from AKI to CKD

Figure1

B Masson CON

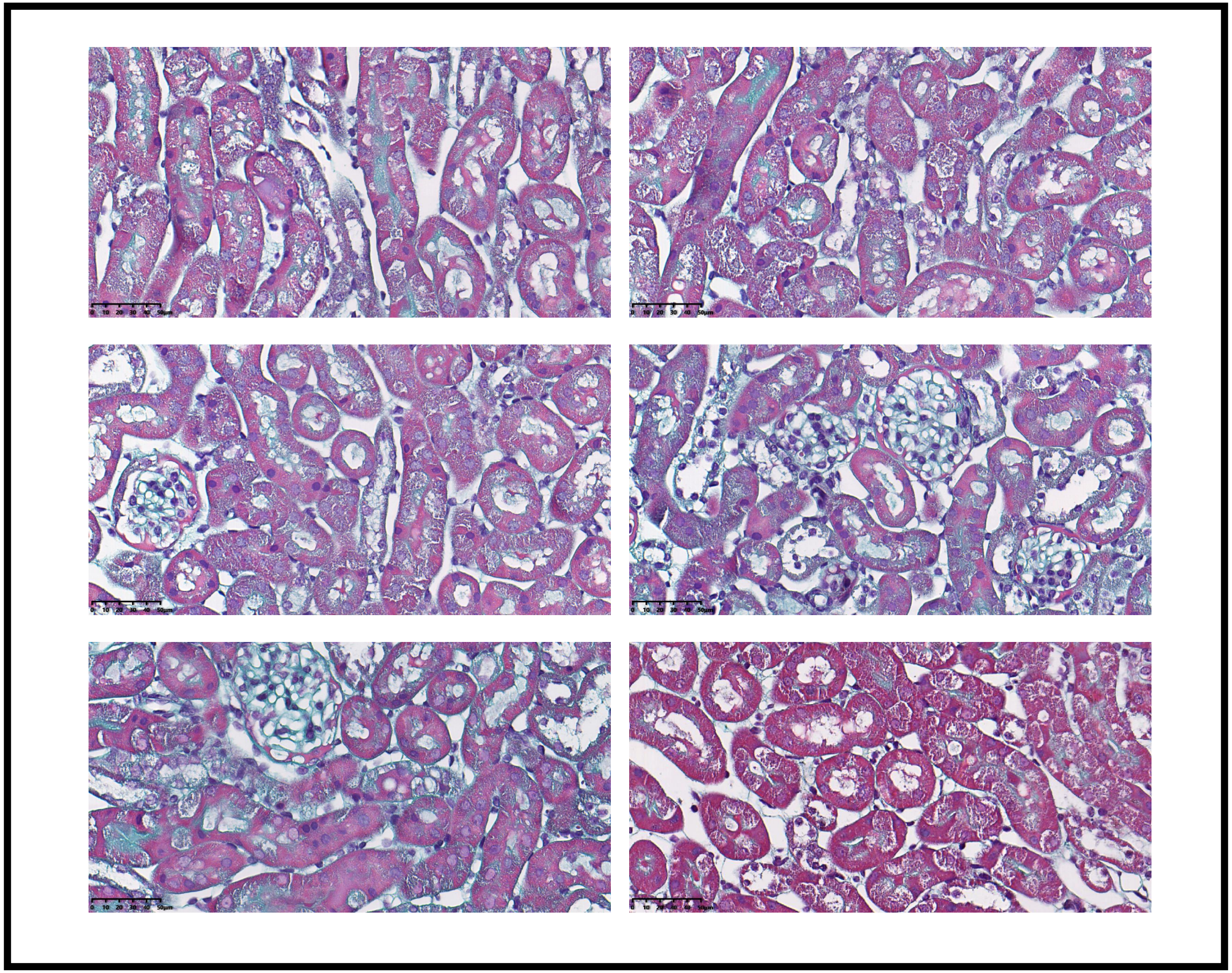

Figure1

B **Masson 2CP**

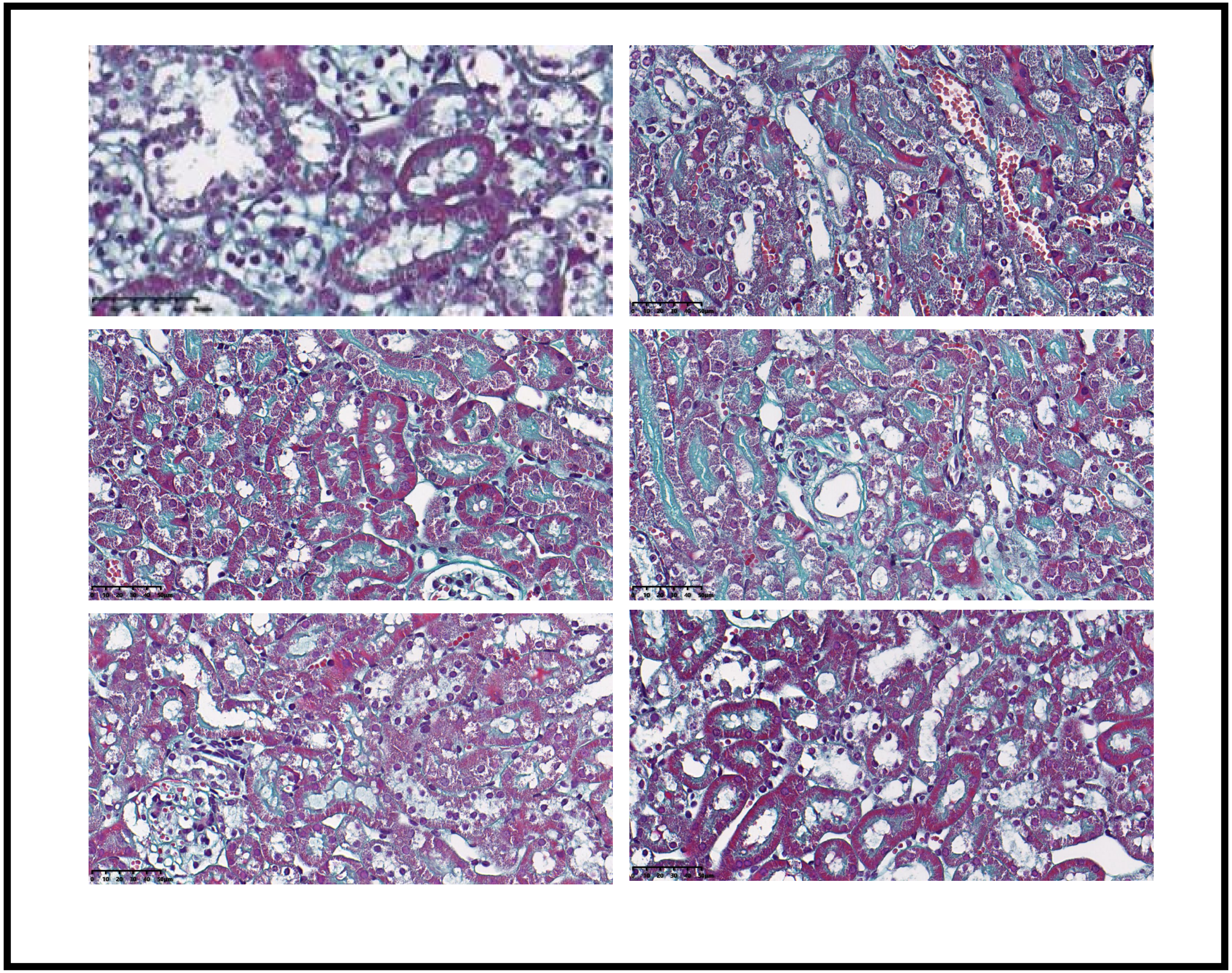

Figure1

B Masson 3CP

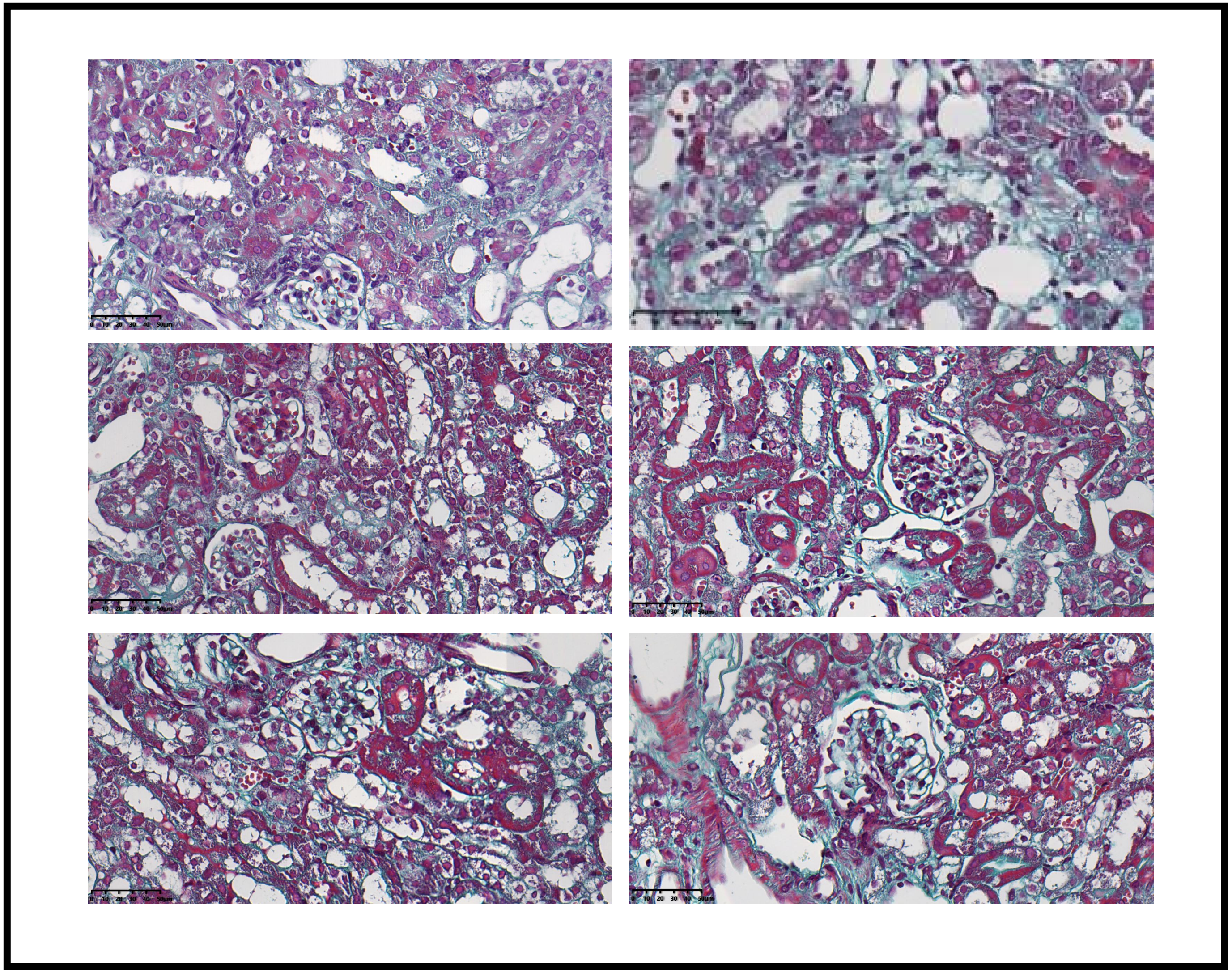

Figure1

B Masson 4CP

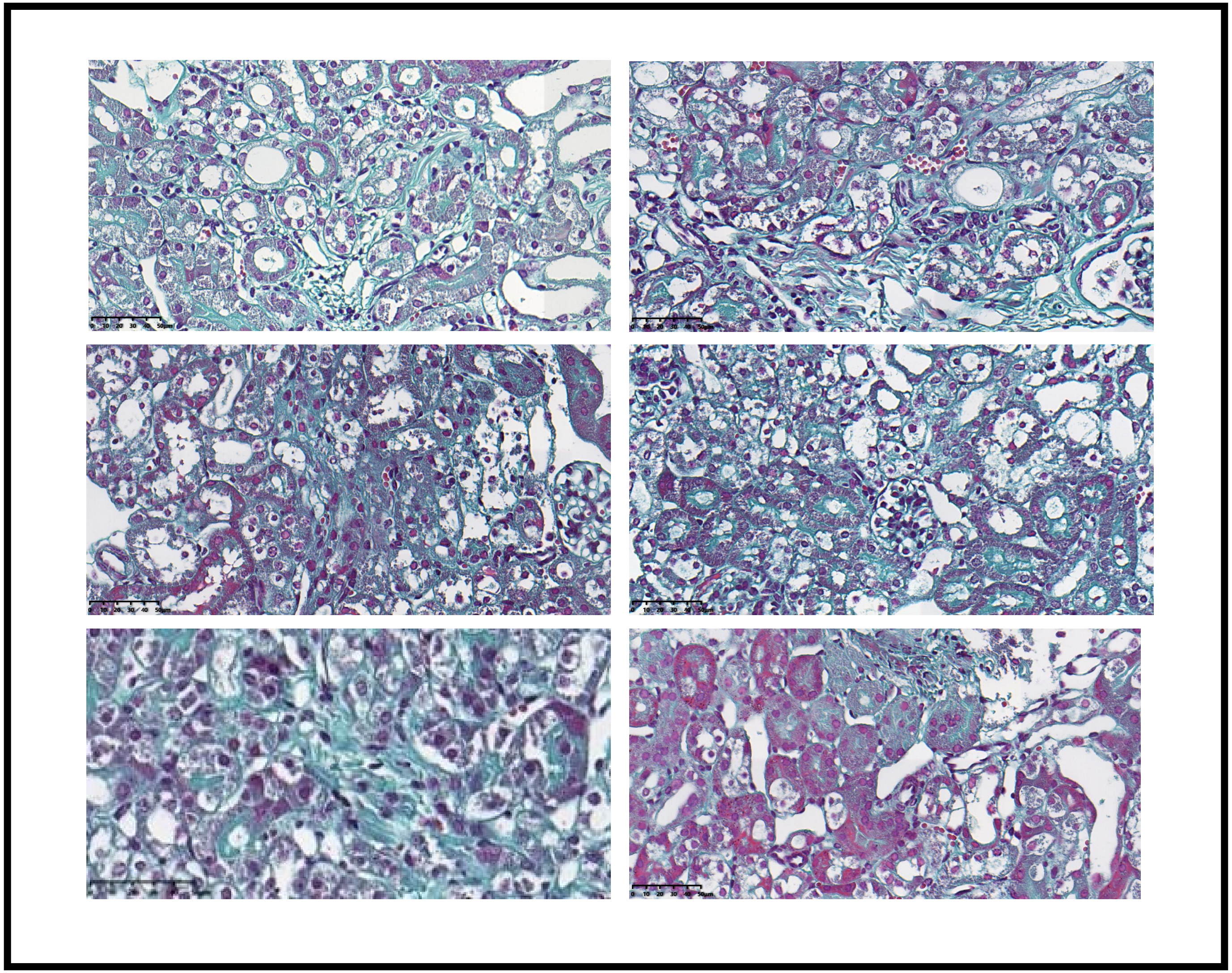

Figure1

B **HE CON**

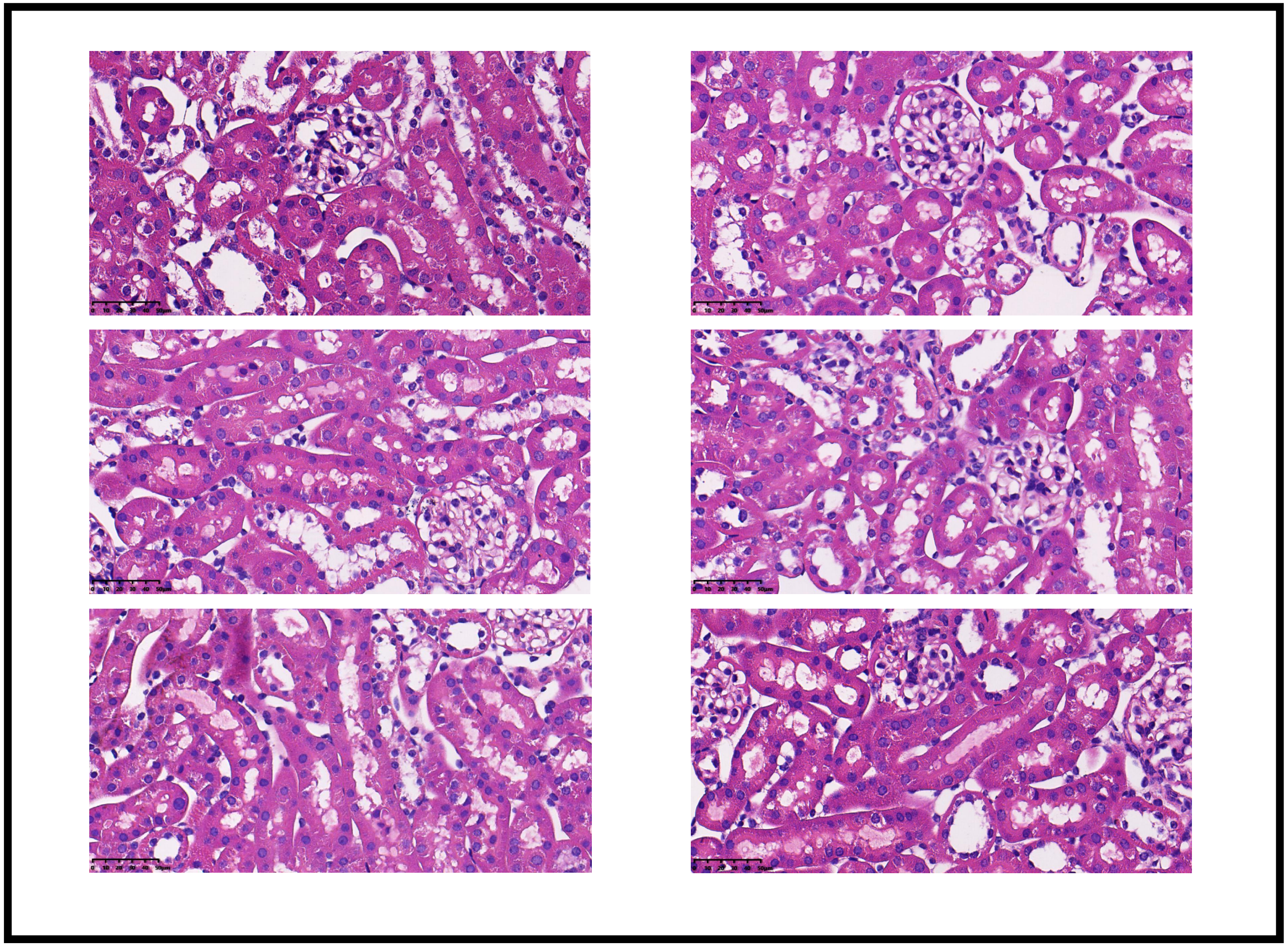

Figure1

B **HE 2CP**

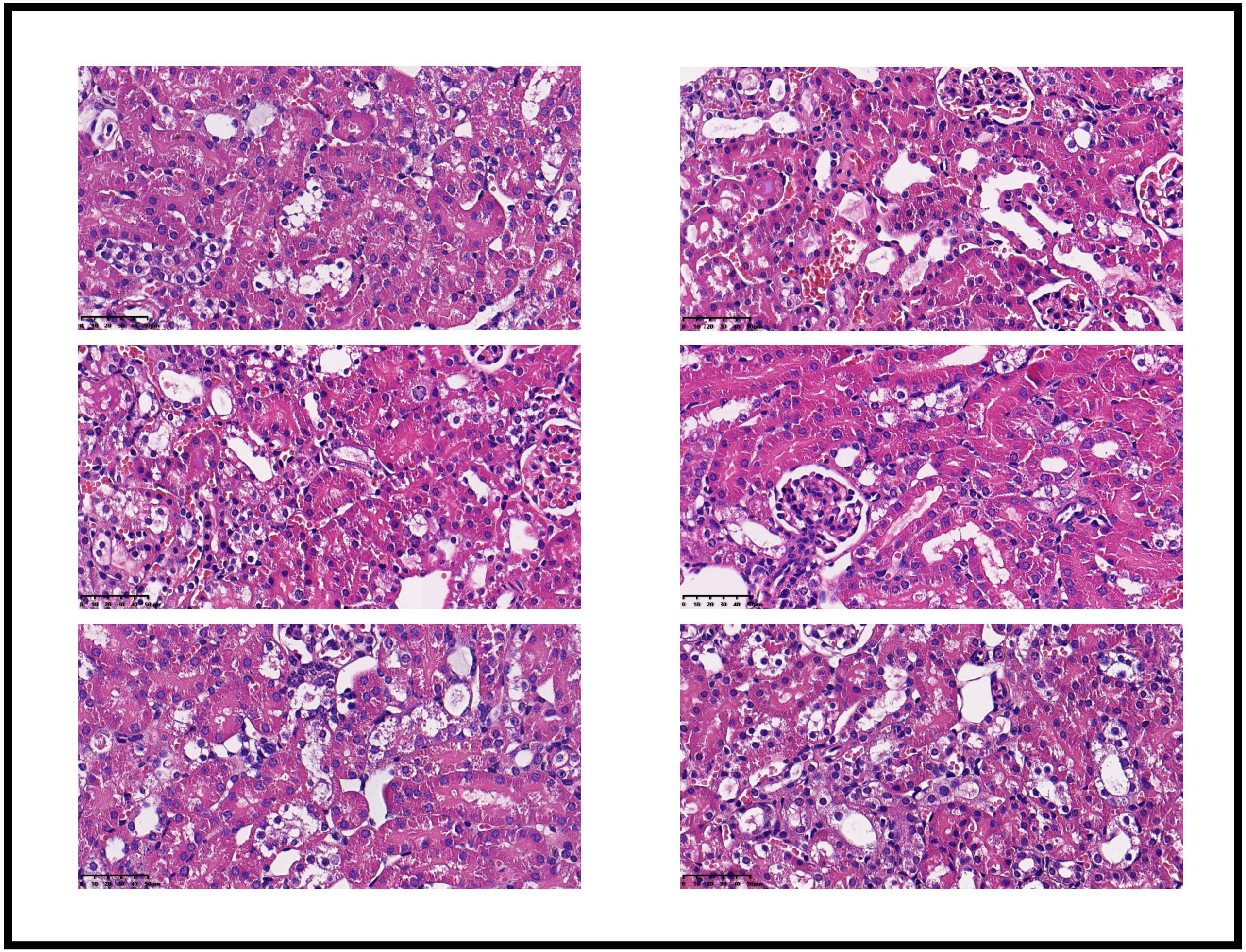

Figure1

B HE 3CP

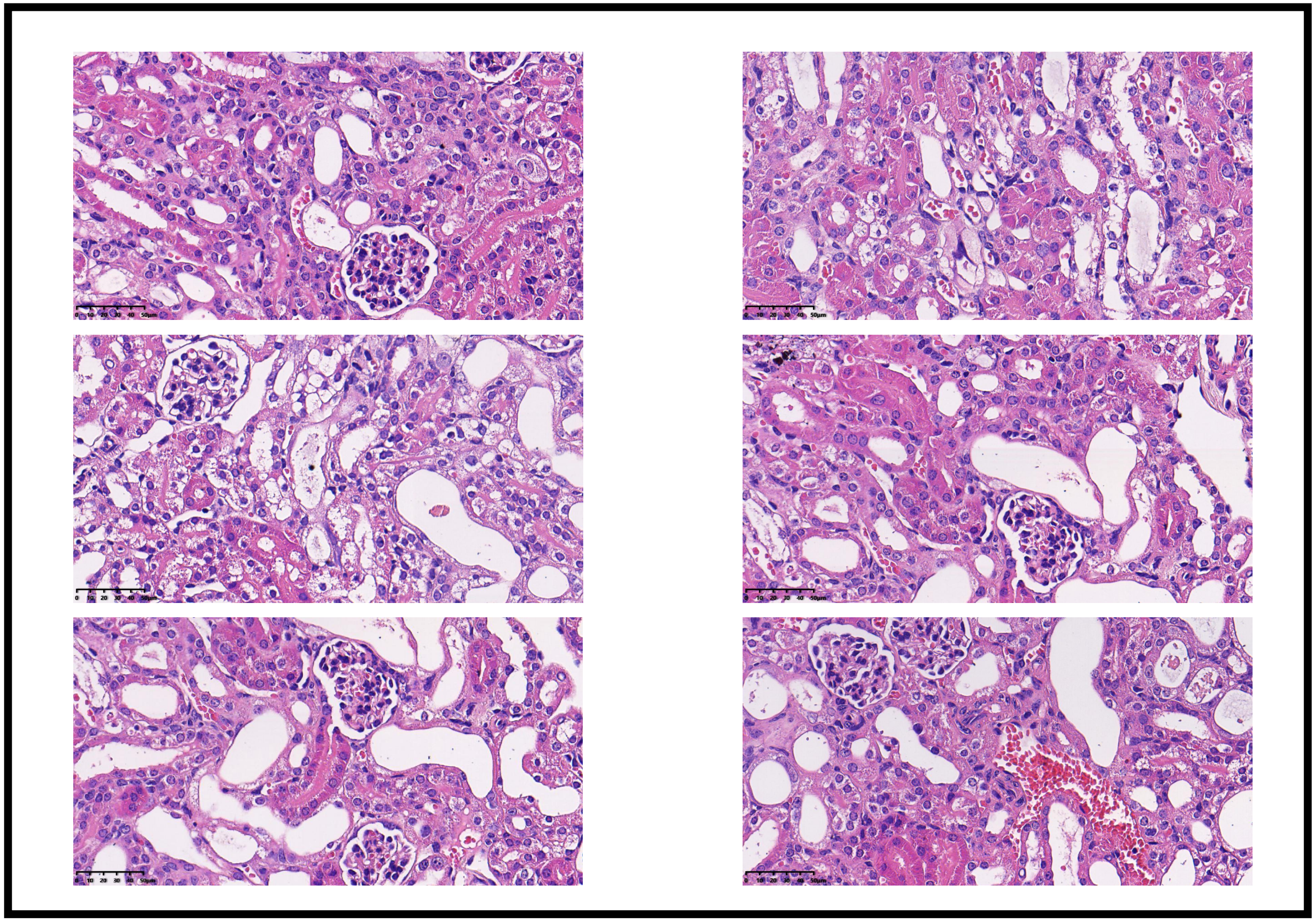

Figure1

B **HE 4CP**

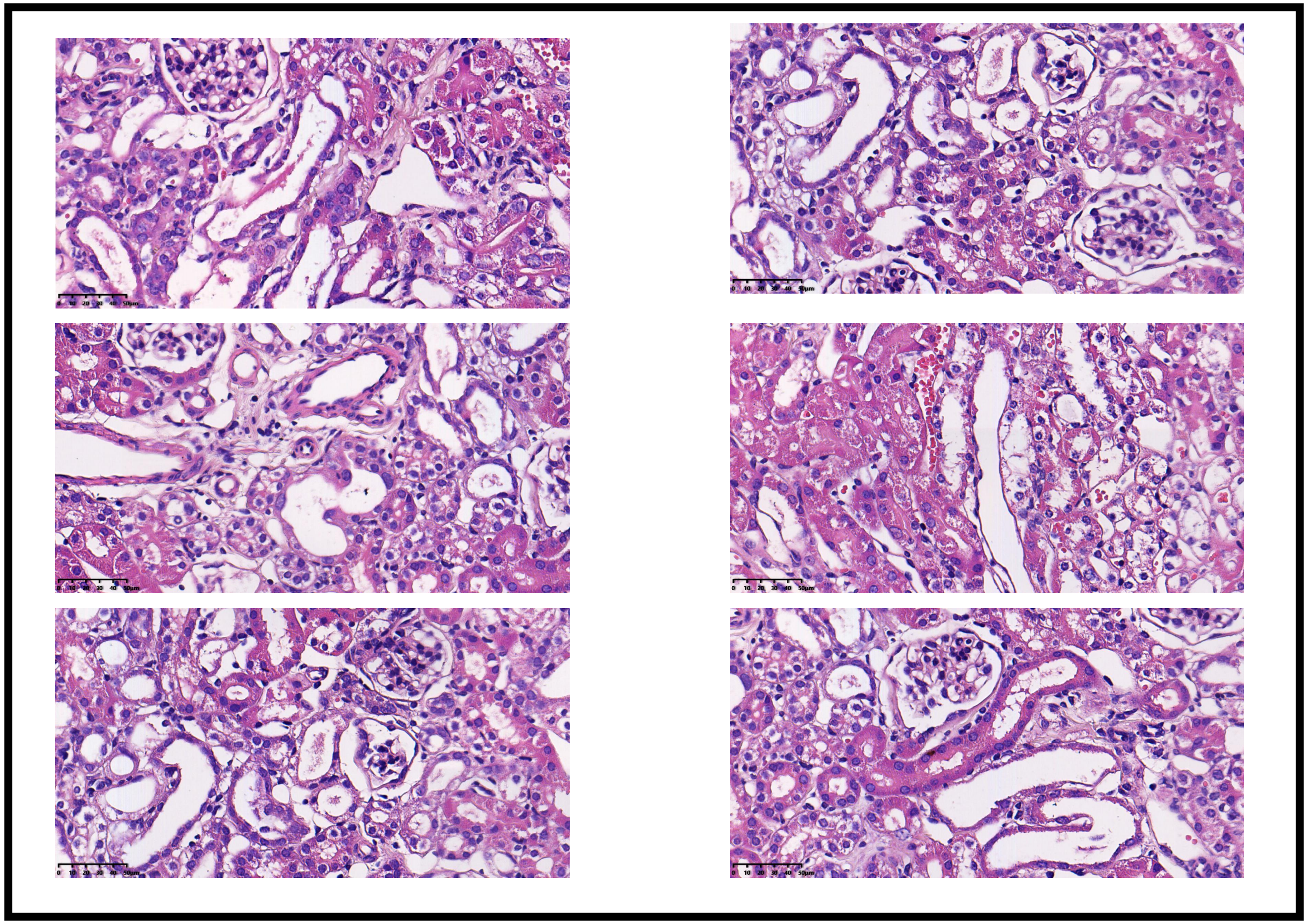

Figure1

C

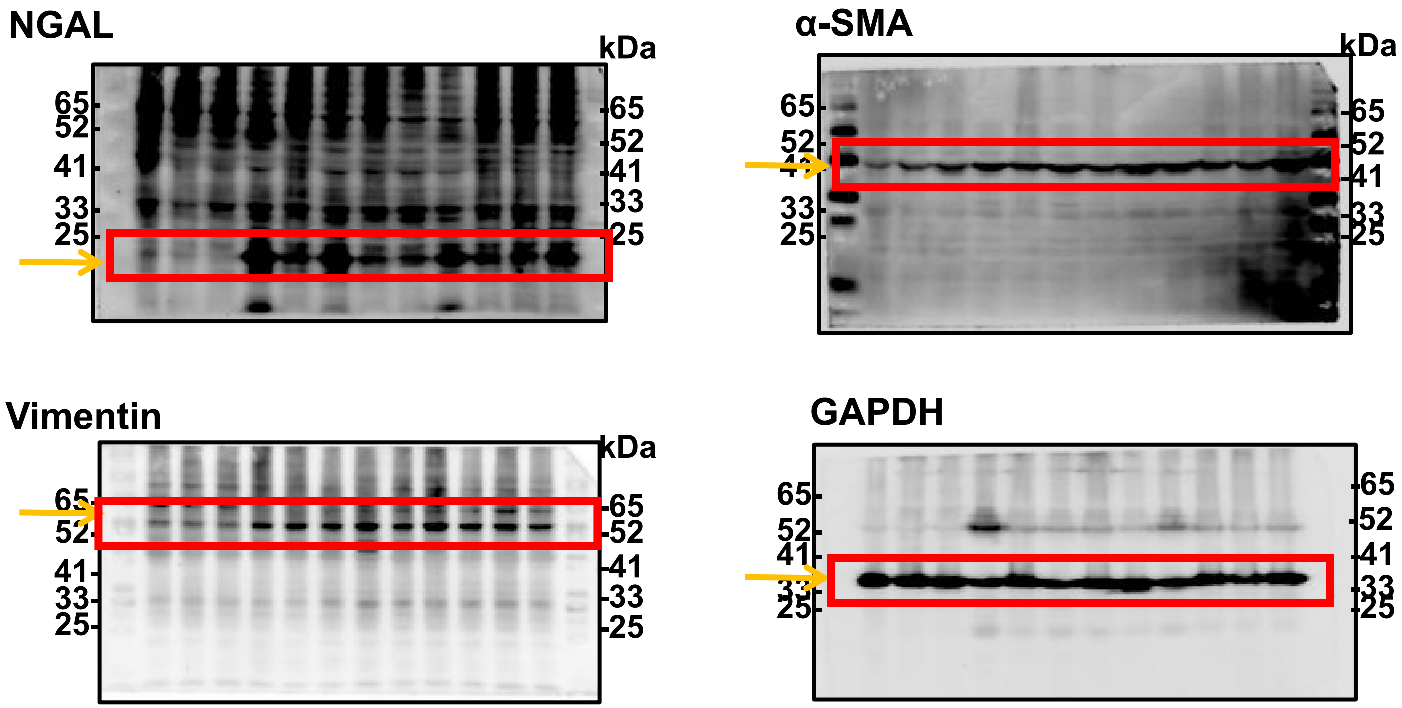

Figure1

D SGK3

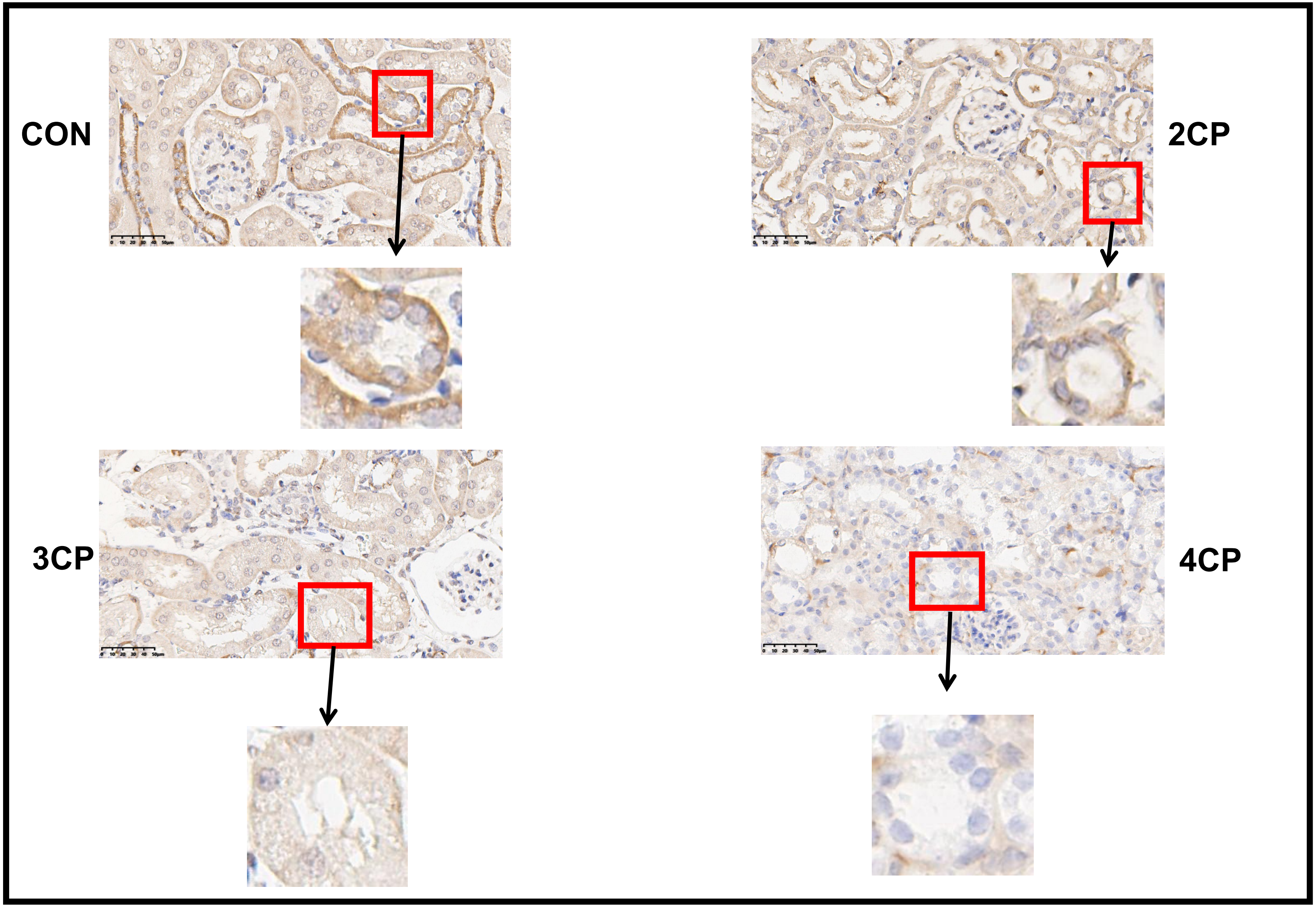

Figure1

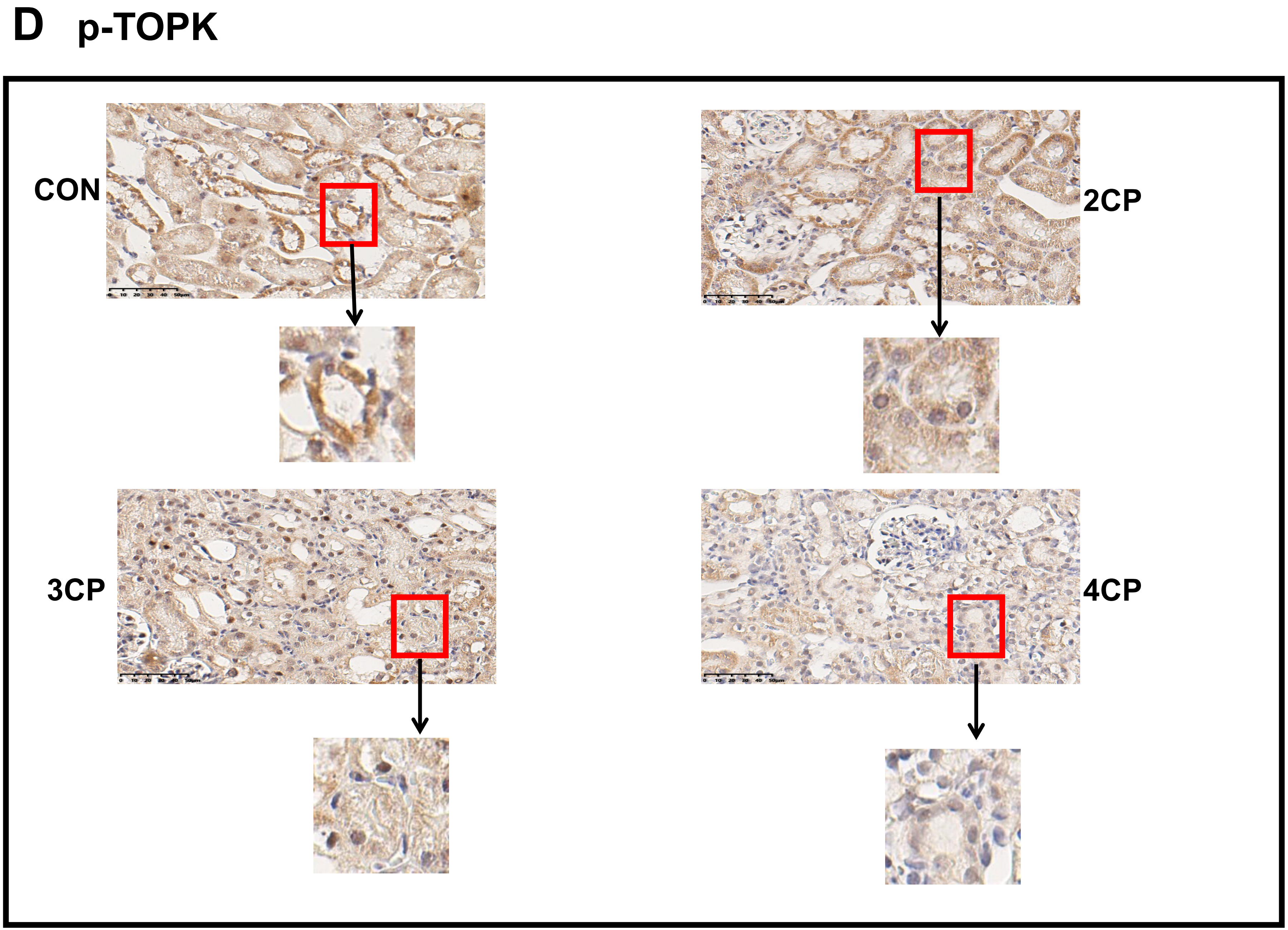

Figure1

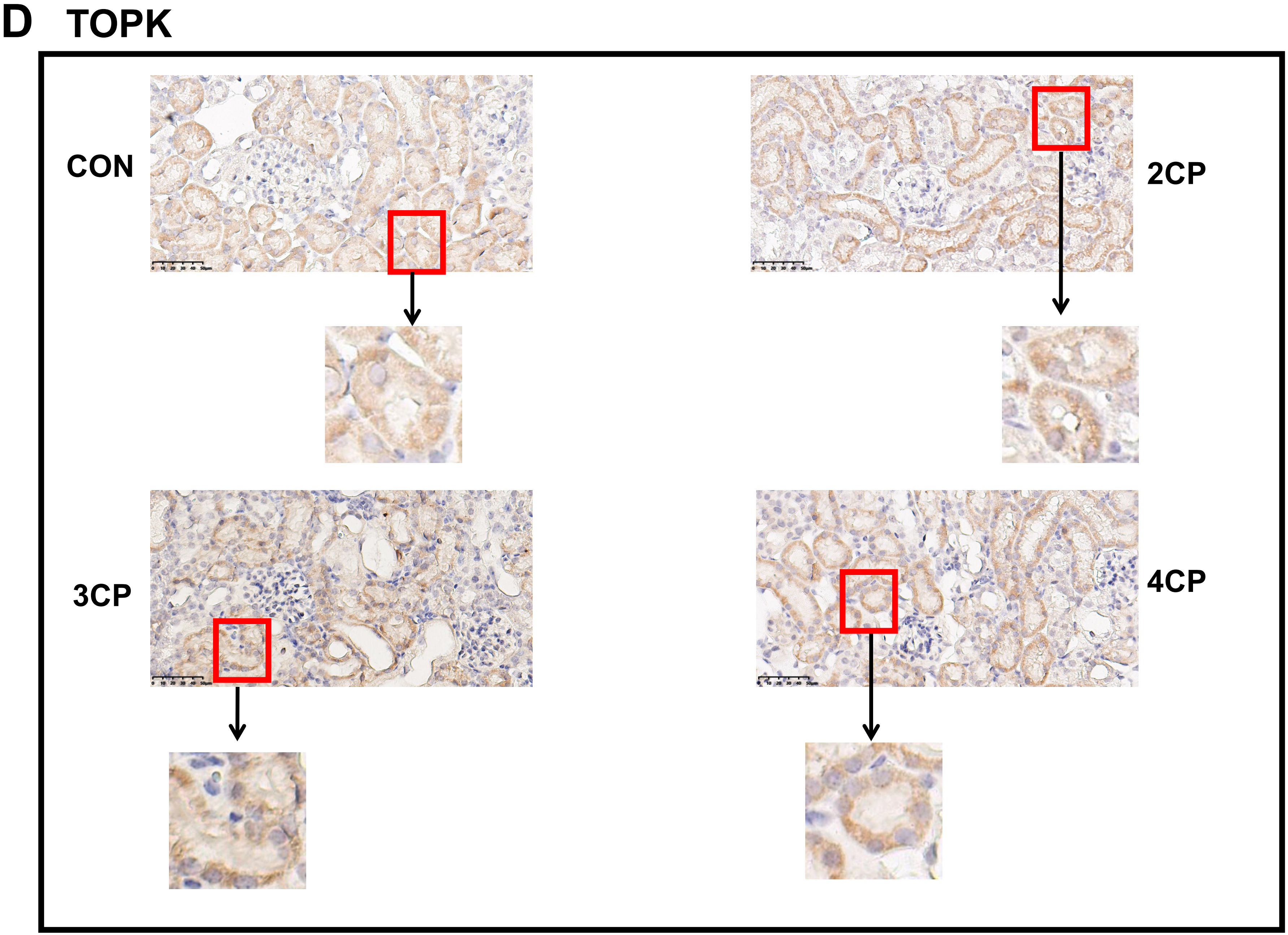

Figure2

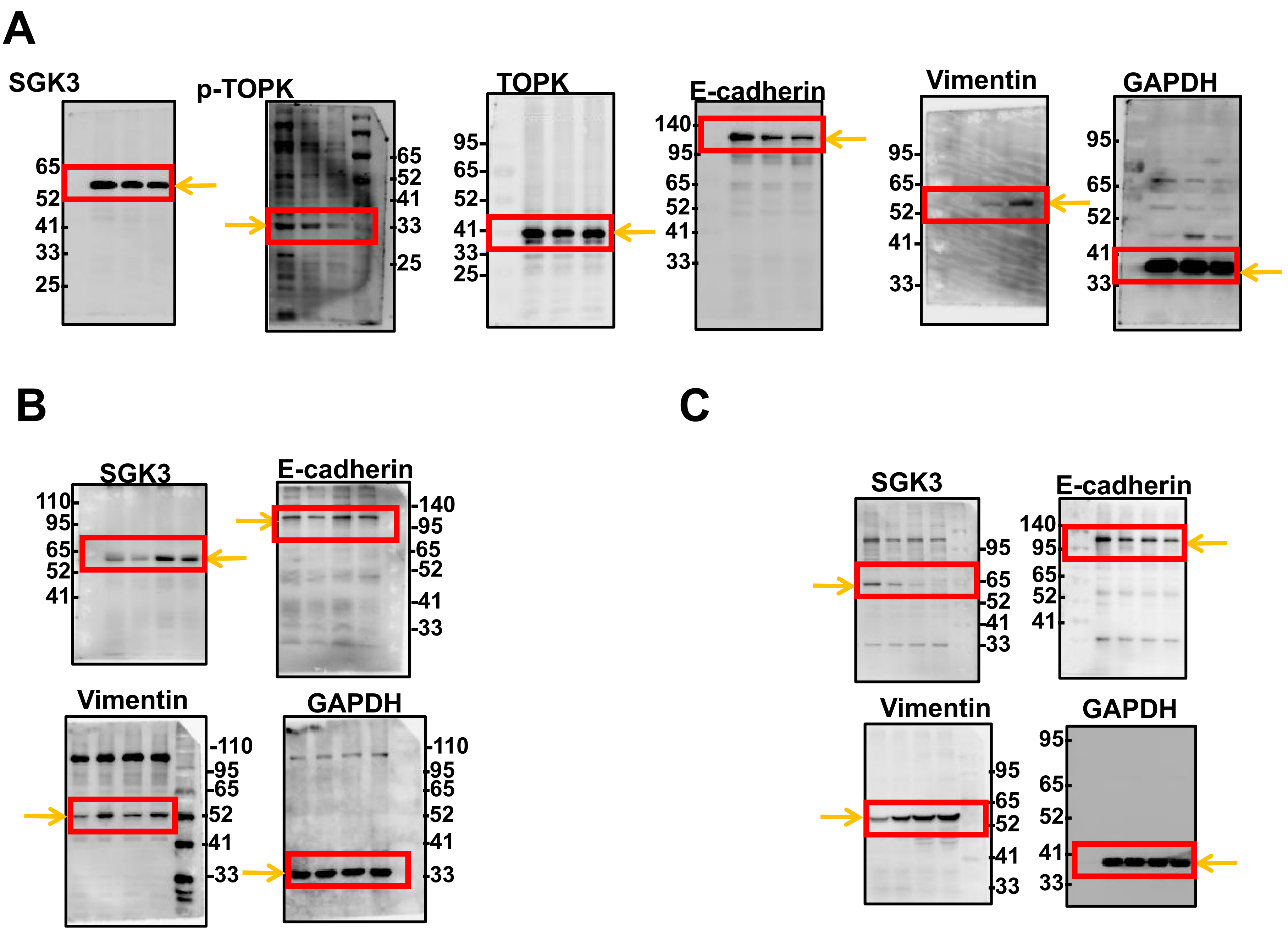
A
SGK3
p-TOPK
TOPK
E-cadherin
Vimentin
GAPDH
B
SGK3
E-cadherin
Vimentin
GAPDH
C
SGK3
E-cadherin
Vimentin
GAPDH

Figure2

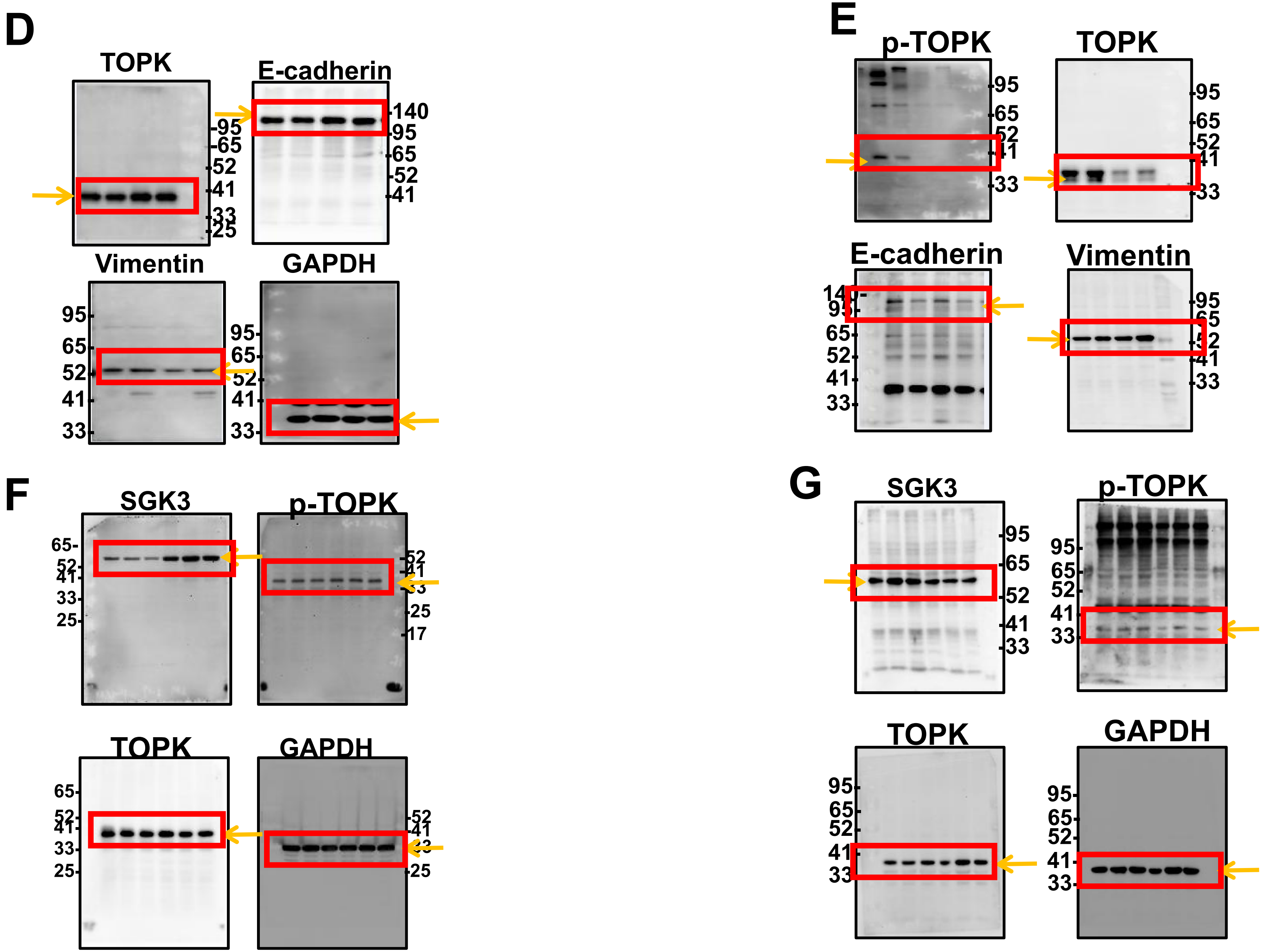
D
TOPK
E-cadherin
Vimentin
GAPDH
140
95
65
52
41
33
25
E
p-TOPK
TOPK
E-cadherin
Vimentin
F
SGK3
p-TOPK
TOPK
GAPDH
17
G
SGK3
p-TOPK
TOPK
GAPDH

Figure2

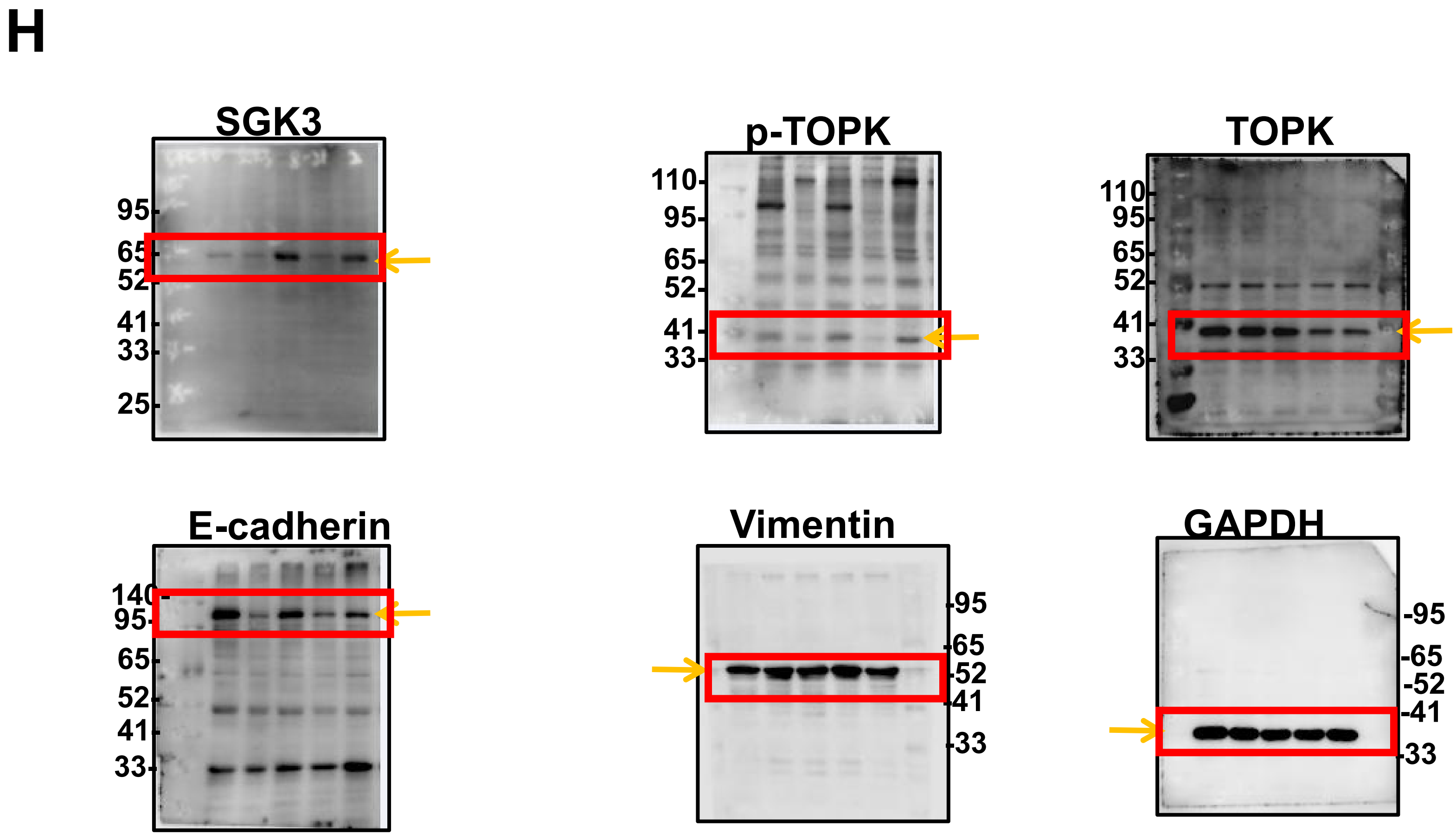
H
SGK3
95
65
52
41
33
25
p-TOPK
110
95
65
52
41
33
TOPK
110
95
65
52
41
33
E-cadherin
140
95
65
52
41
33
Vimentin
95
65
52
41
33
GAPDH
95
65
52
41
33

Figure3

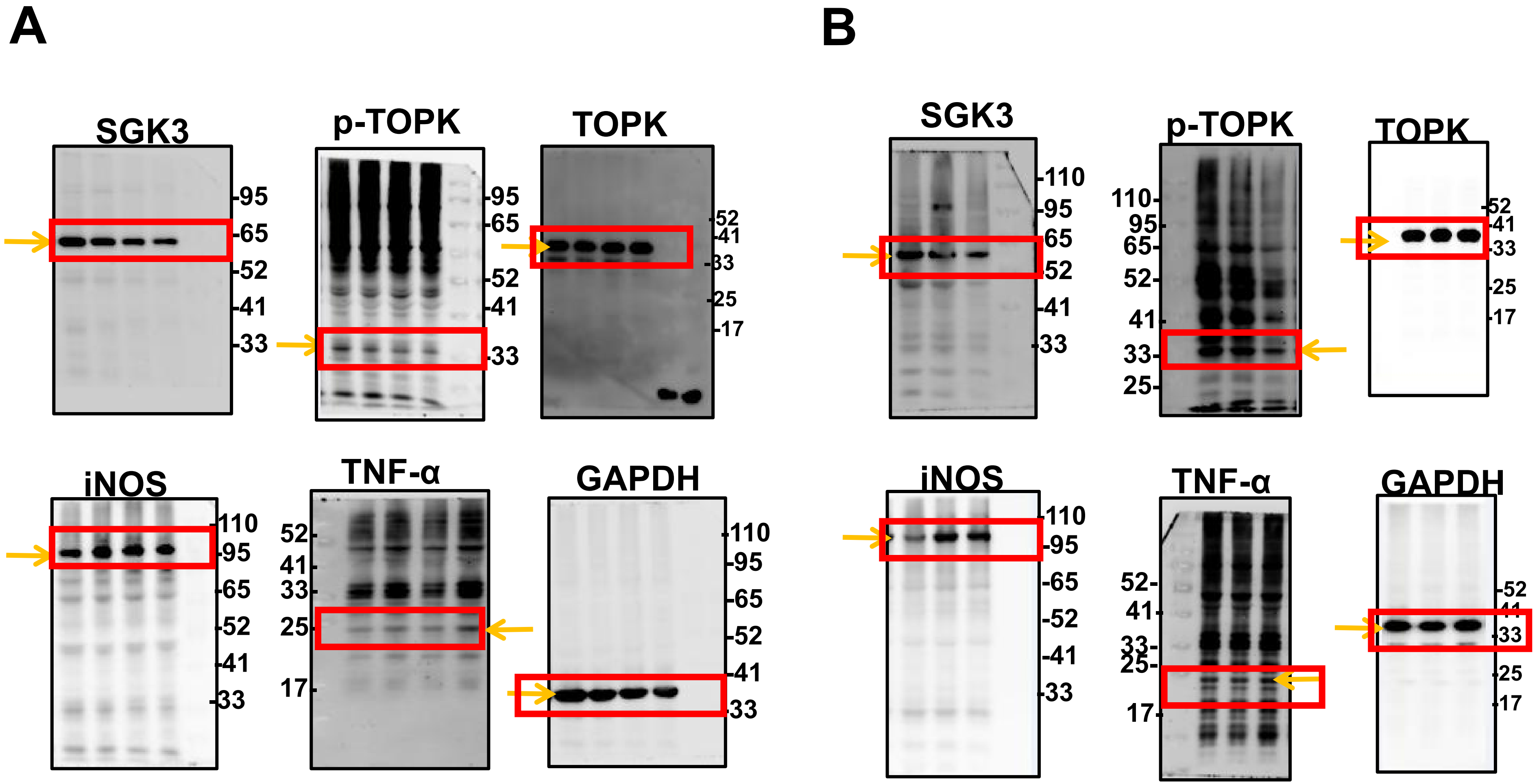
A
SGK3
95
65
52
41
33
p-TOPK
95
65
52
41
33
TOPK
52
41
33
25
17
iNOS
110
95
65
52
41
33
TNF-α
52
41
33
25
17
GAPDH
110
95
65
52
41
33
B
SGK3
110
95
65
52
41
33
p-TOPK
110
95
65
52
41
33
25
TOPK
52
41
33
25
17
iNOS
110
95
65
52
41
33
TNF-α
52
41
33
25
17
GAPDH
52
41
33
25
17

Figure3

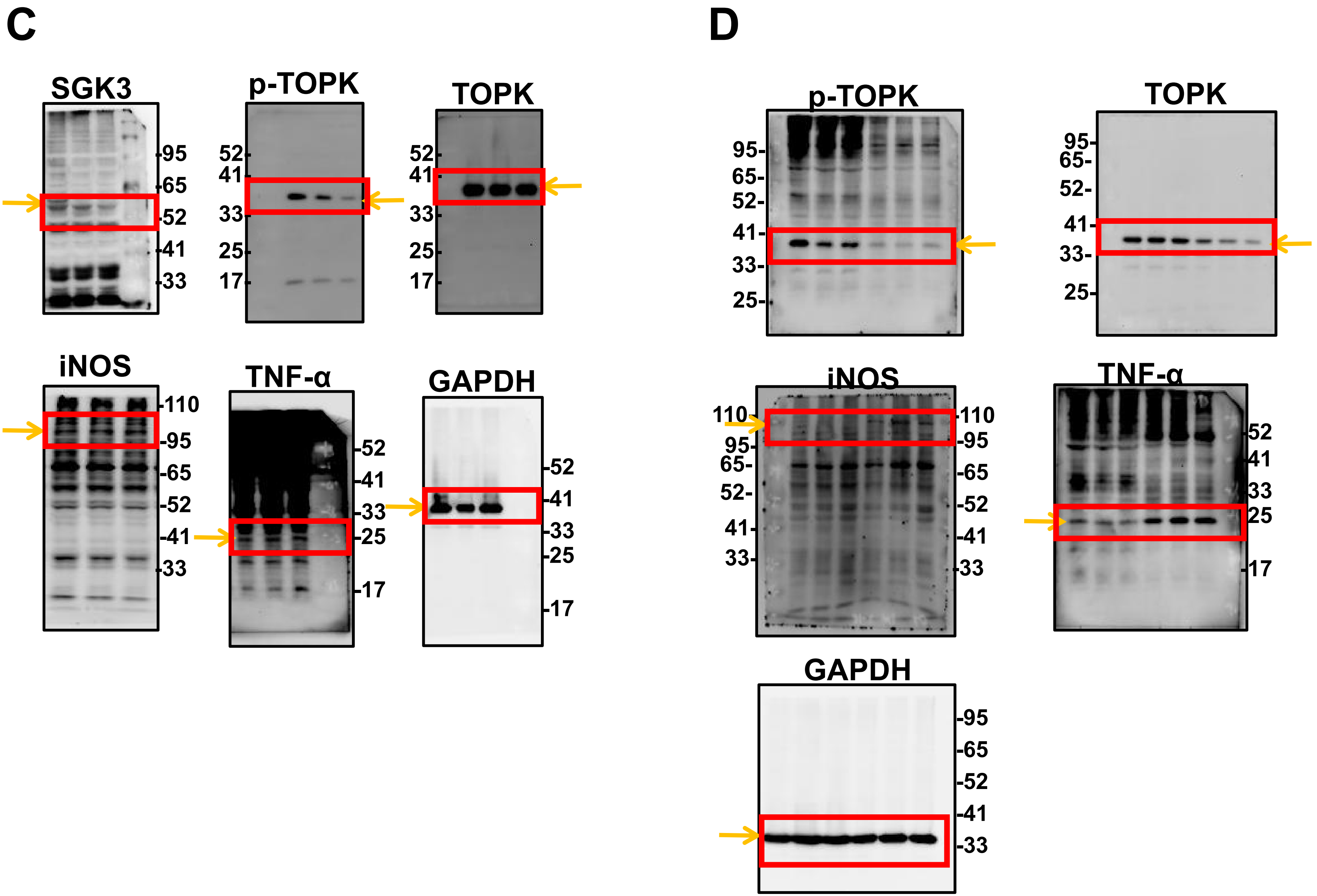

C
SGK3
95
65
52
41
33
p-TOPK
52
41
33
25
17
TOPK
52
41
33
25
17
iNOS
110
95
65
52
41
33
TNF-α
52
41
33
25
17
GAPDH
52
41
33
25
17
D
p-TOPK
95
65
52
41
33
25
TOPK
95
65
52
41
33
25
iNOS
110
95
65
52
41
33
TNF-α
52
41
33
25
17
GAPDH
95
65
52
41
33

Figure3

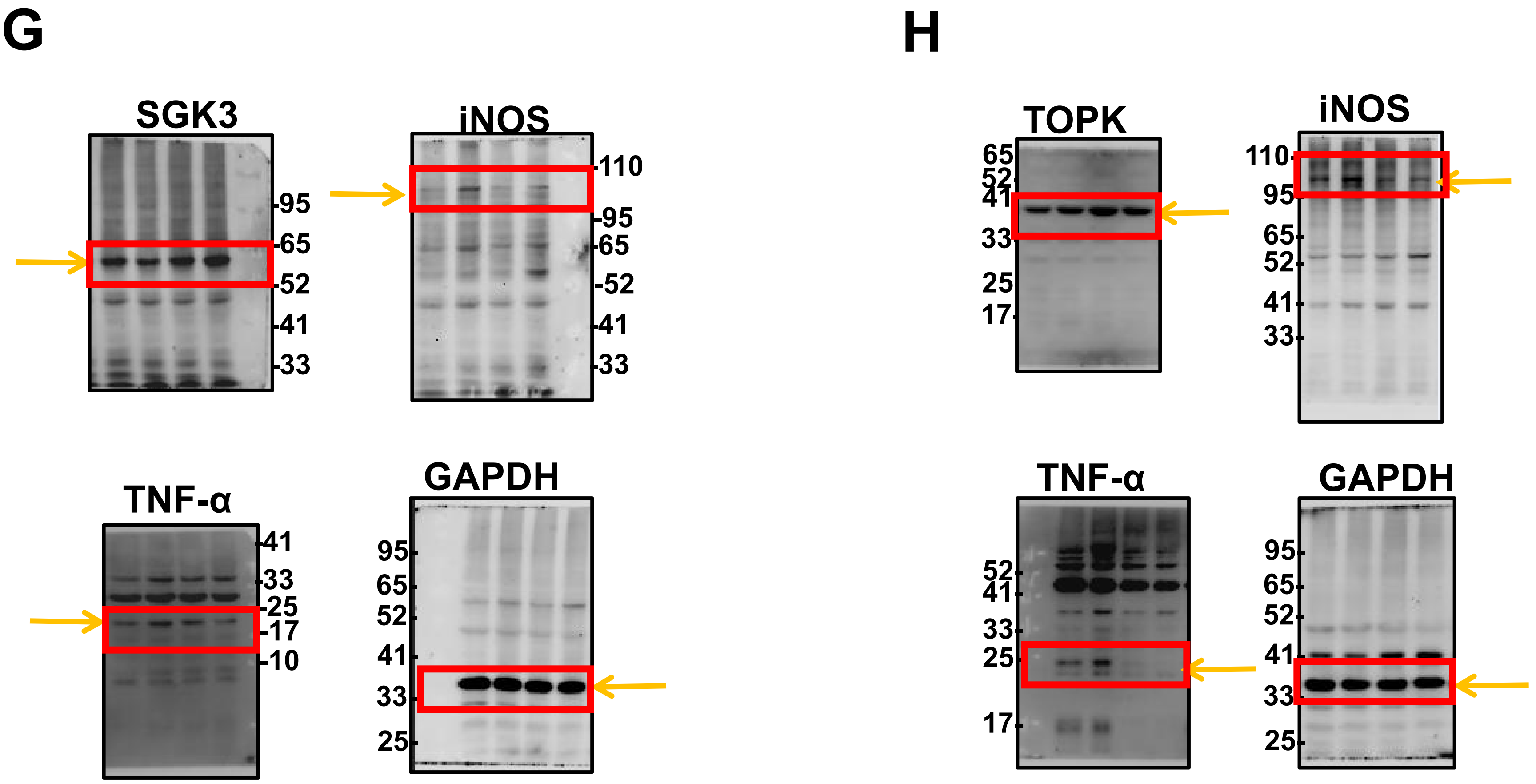

G
SGK3
95
65
52
41
33
iNOS
110
95
65
52
41
33
TNF-α
41
33
25
17
10
GAPDH
95
65
52
41
33
25
H
TOPK
65
52
41
33
25
17
iNOS
110
95
65
52
41
33
TNF-α
52
41
33
25
17
GAPDH
95
65
52
41
33
25

Figure3

I

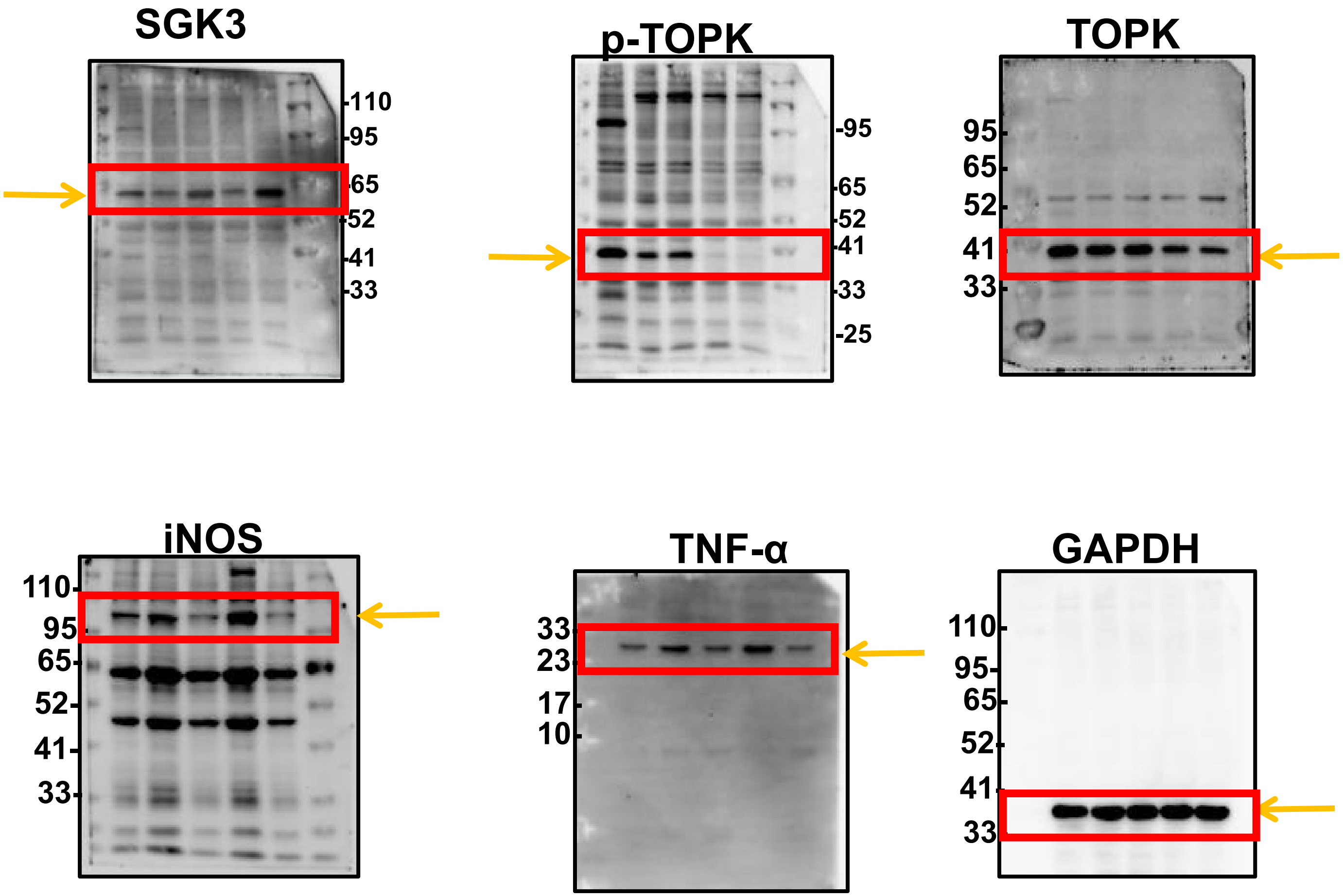
SGK3
110
95
65
52
41
33
p-TOPK
95
65
52
41
33
25
TOPK
95
65
52
41
33
iNOS
110
95
65
52
41
33
TNF-α
33
23
17
10
GAPDH
110
95
65
52
41
33

Figure4

C **Masson 5/6 Nephrectomy**

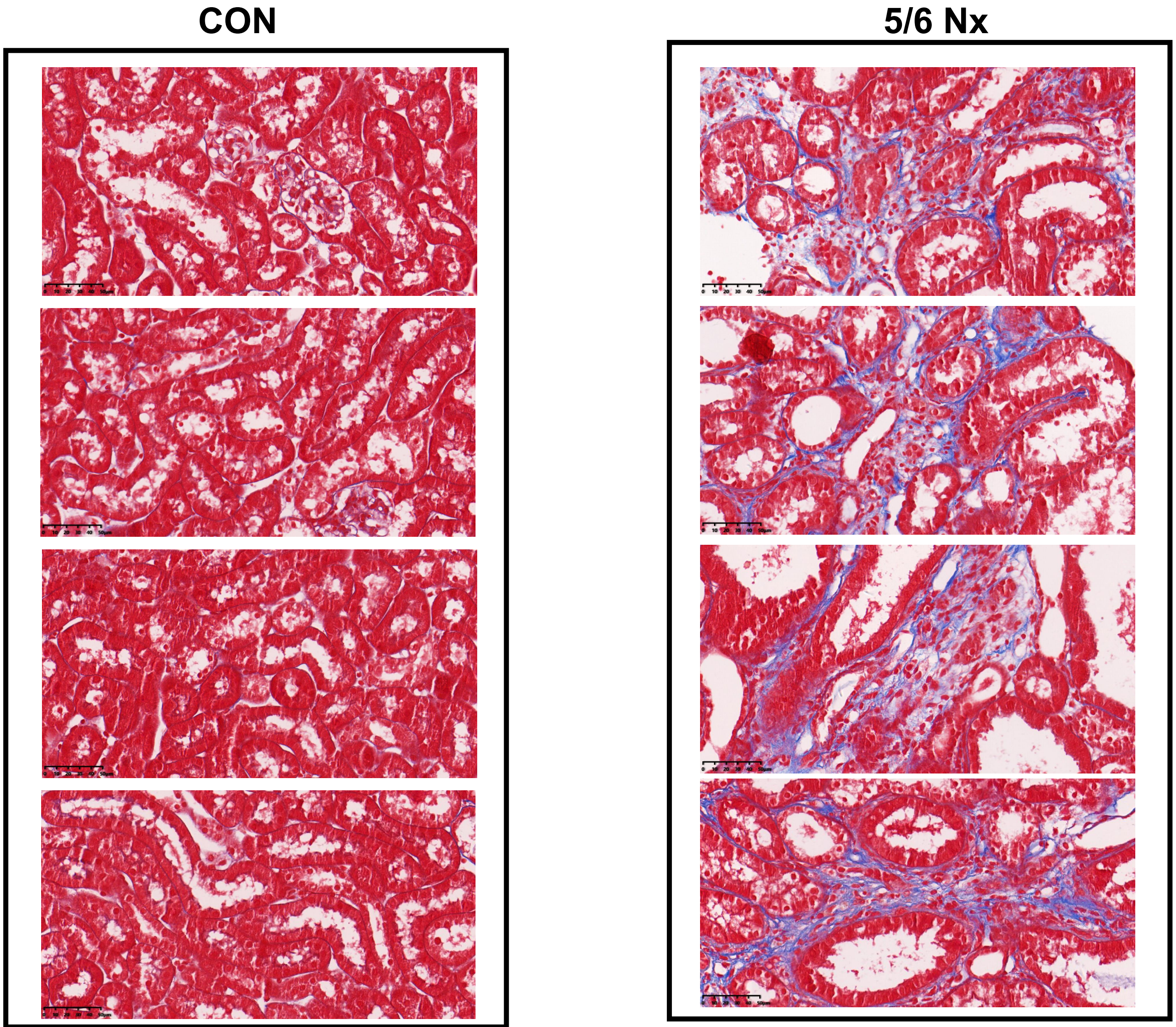

Figure4

C HE 5/6 Nephrectomy

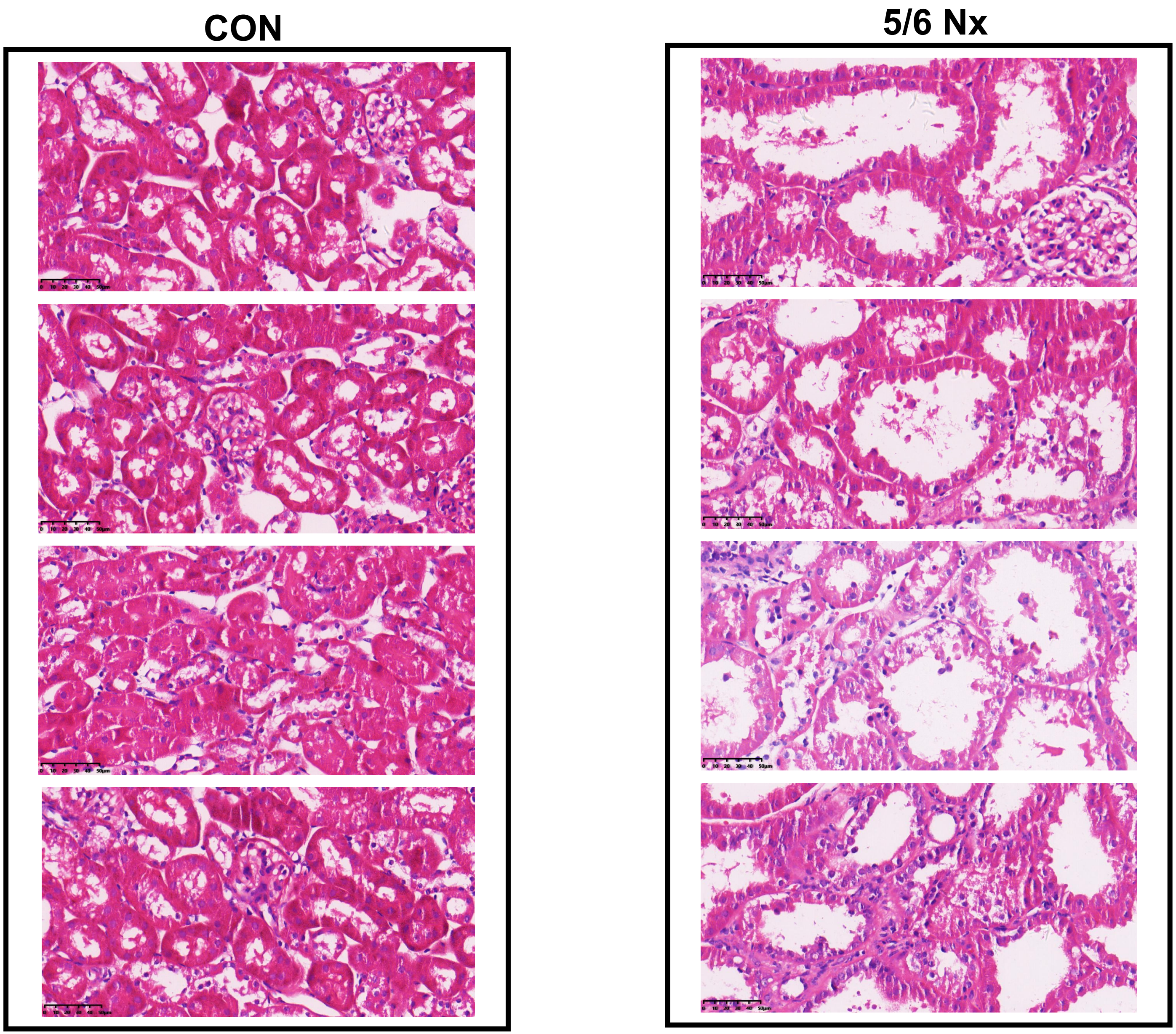

Figure4

D **Masson UUO**

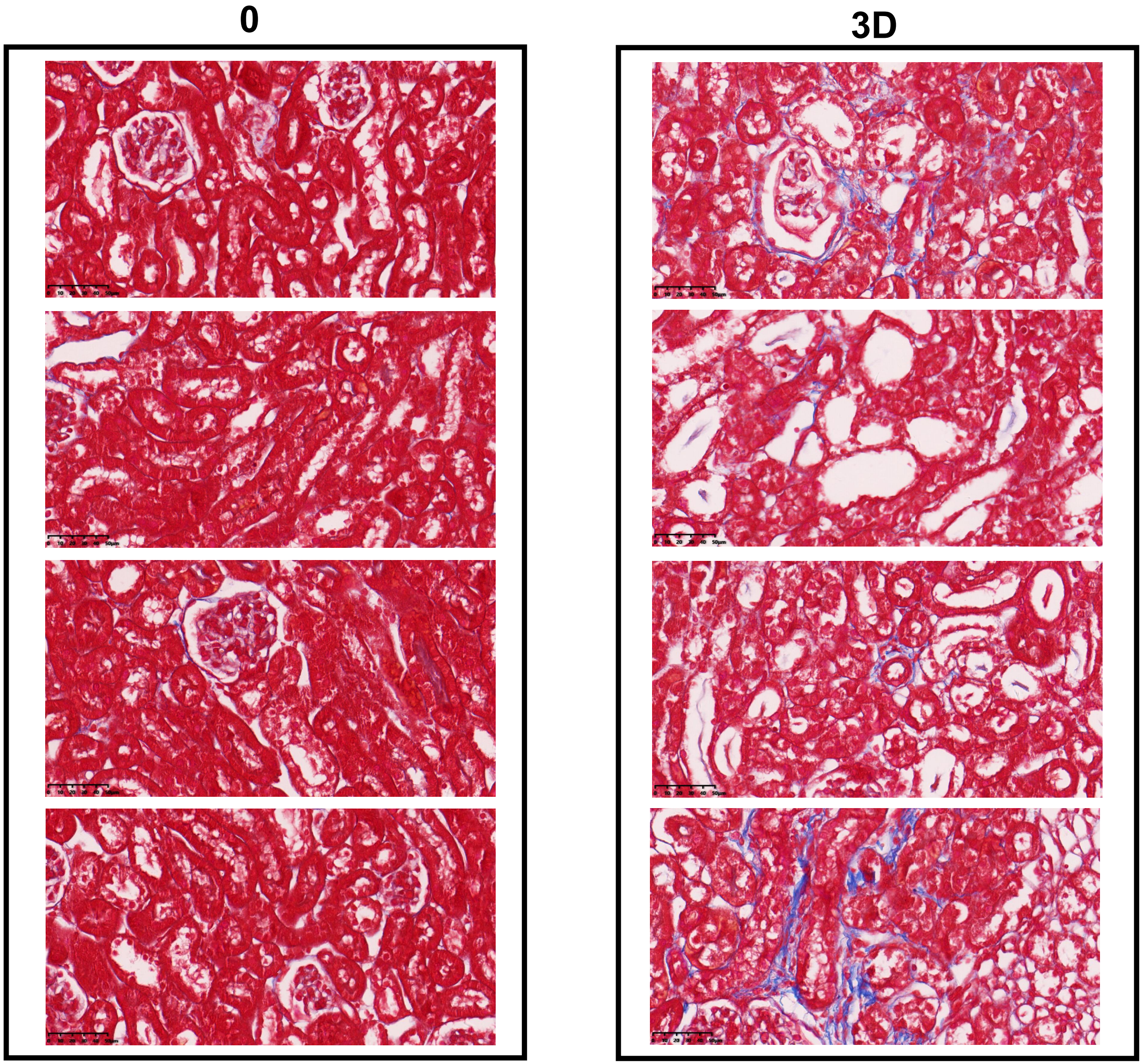

Figure4

D Masson UUO

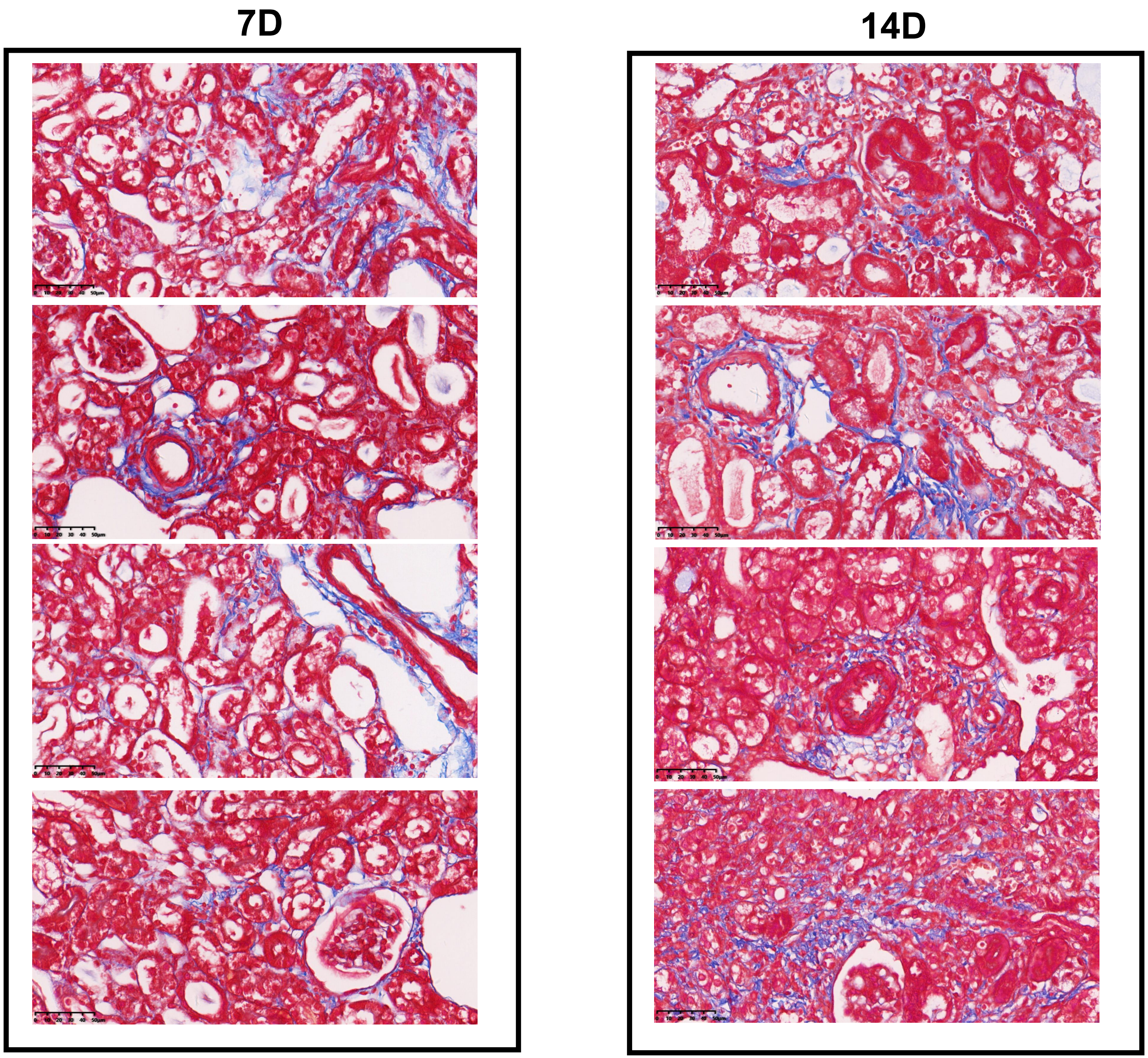

Figure4

D HE UUO

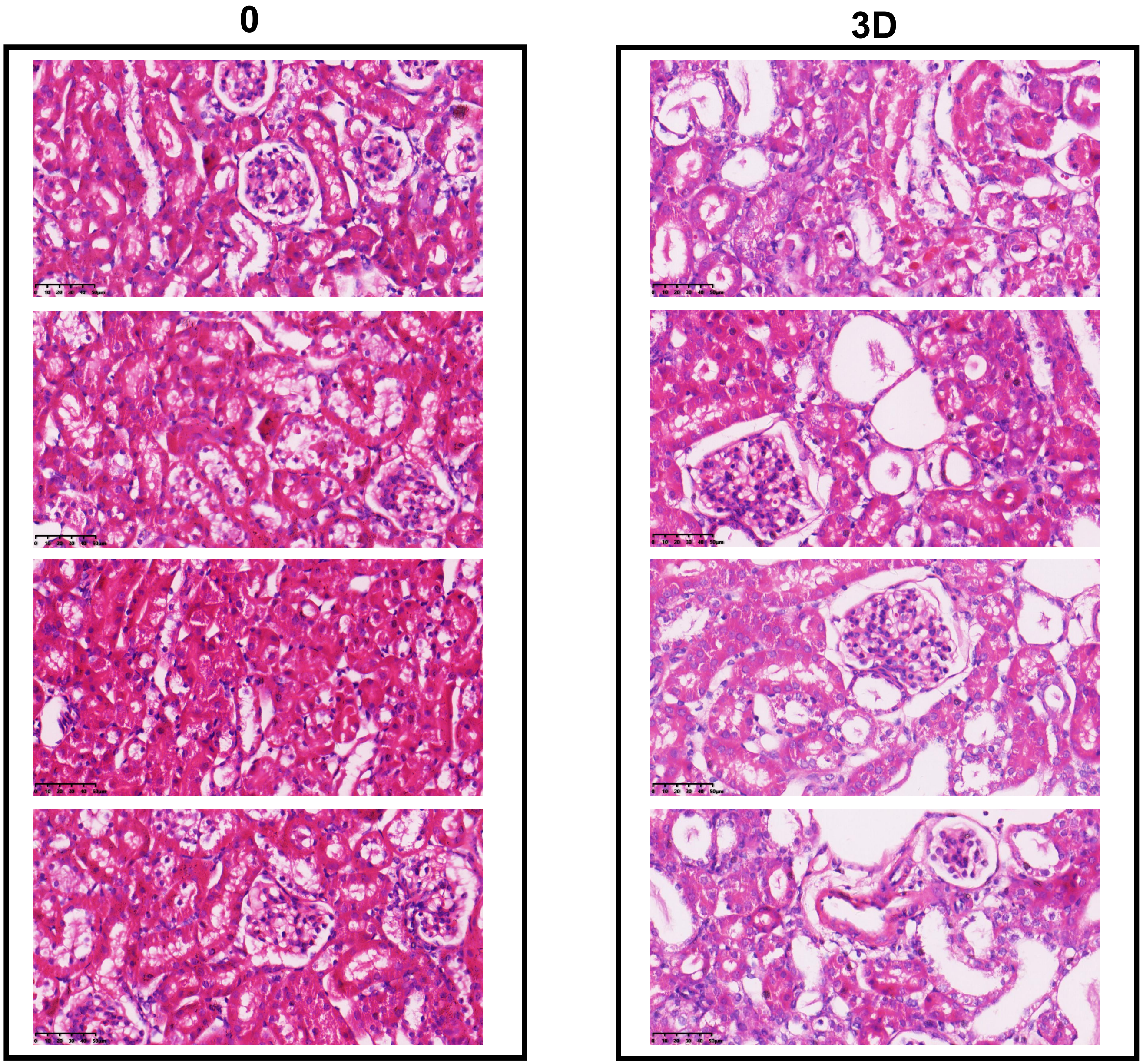

Figure4

D HE UUO

7D 14D

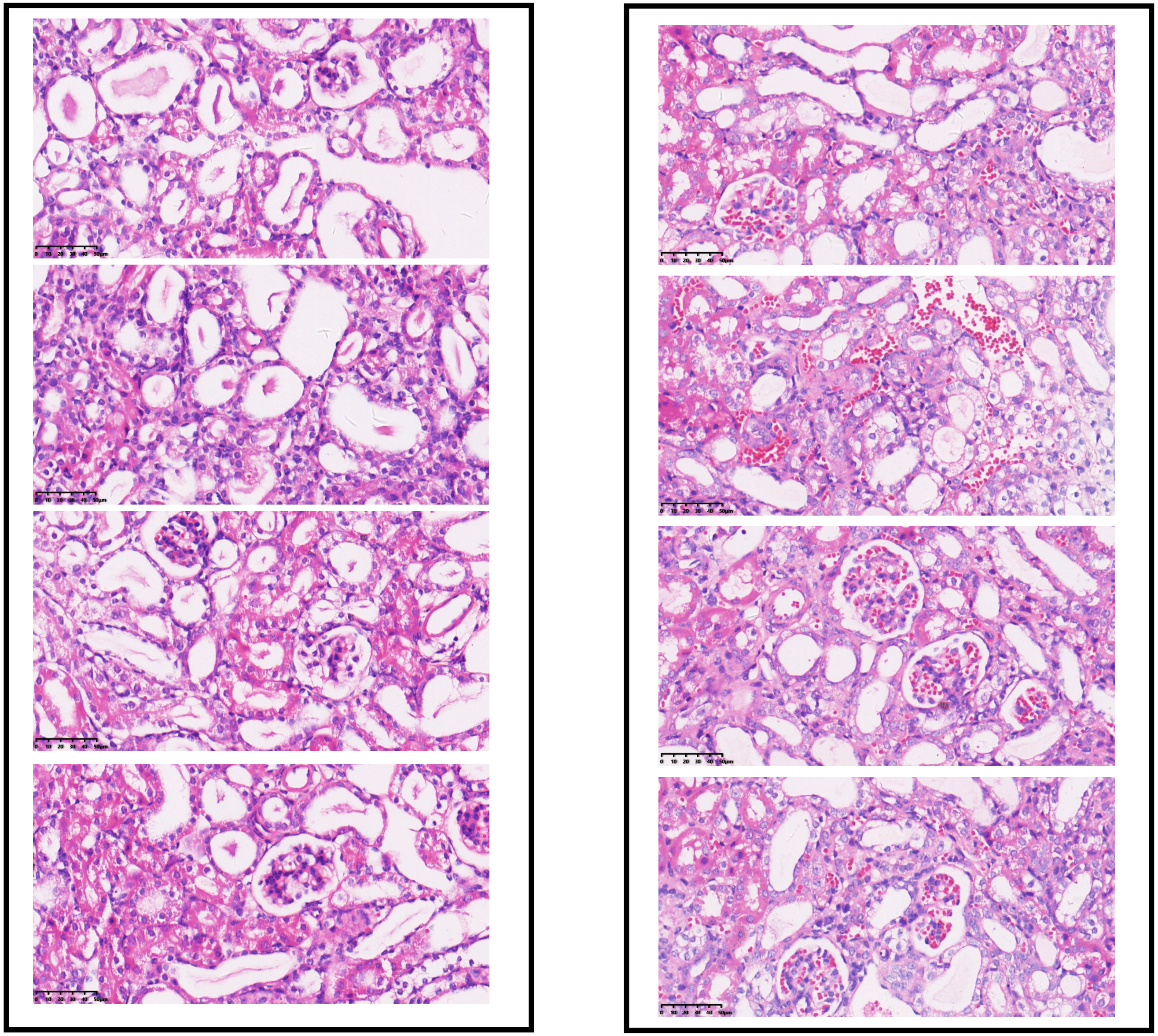

Figure4

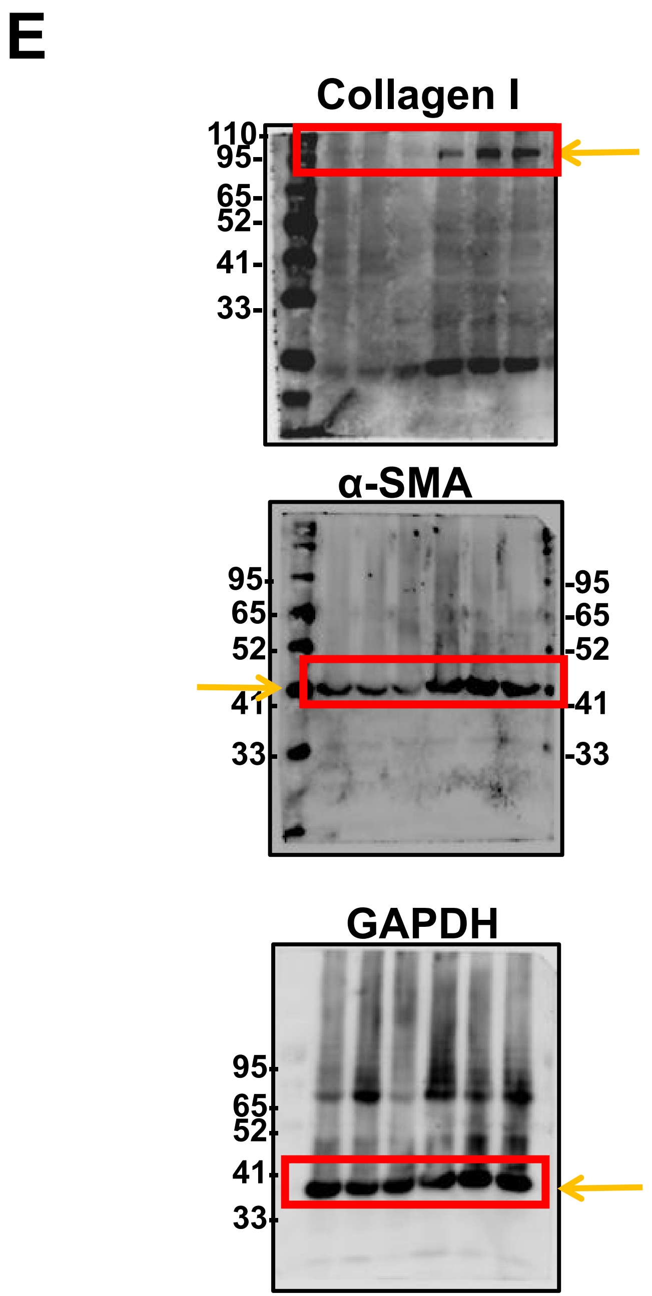
E
Collagen I
110
95
65
52
41
33
α-SMA
95
65
52
41
33
GAPDH
95
65
52
41
33

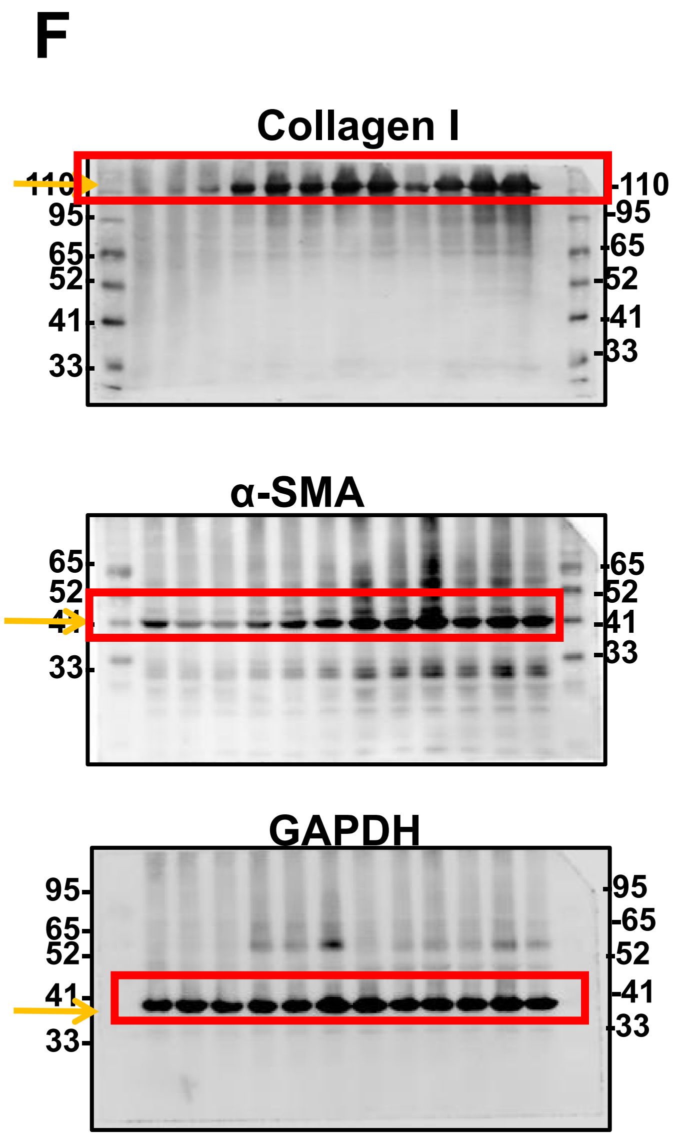
F
Collagen I
95
65
52
41
33
110
α-SMA
65
52
33
41
GAPDH
95
65
52
41
33

Figure4

G 5/6 Nephrectomy IHC

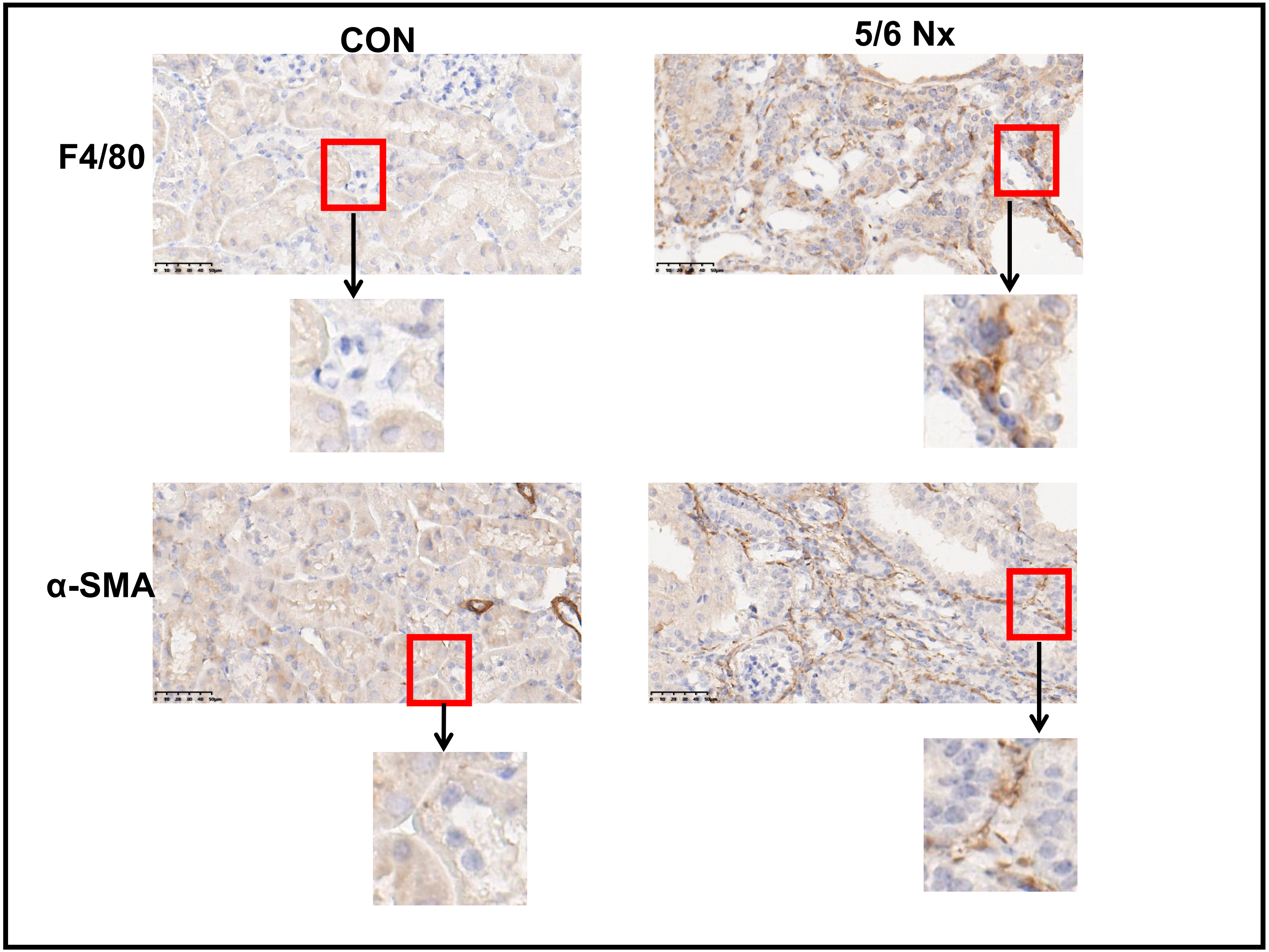

Figure4

G 5/6 Nephrectomy IHC

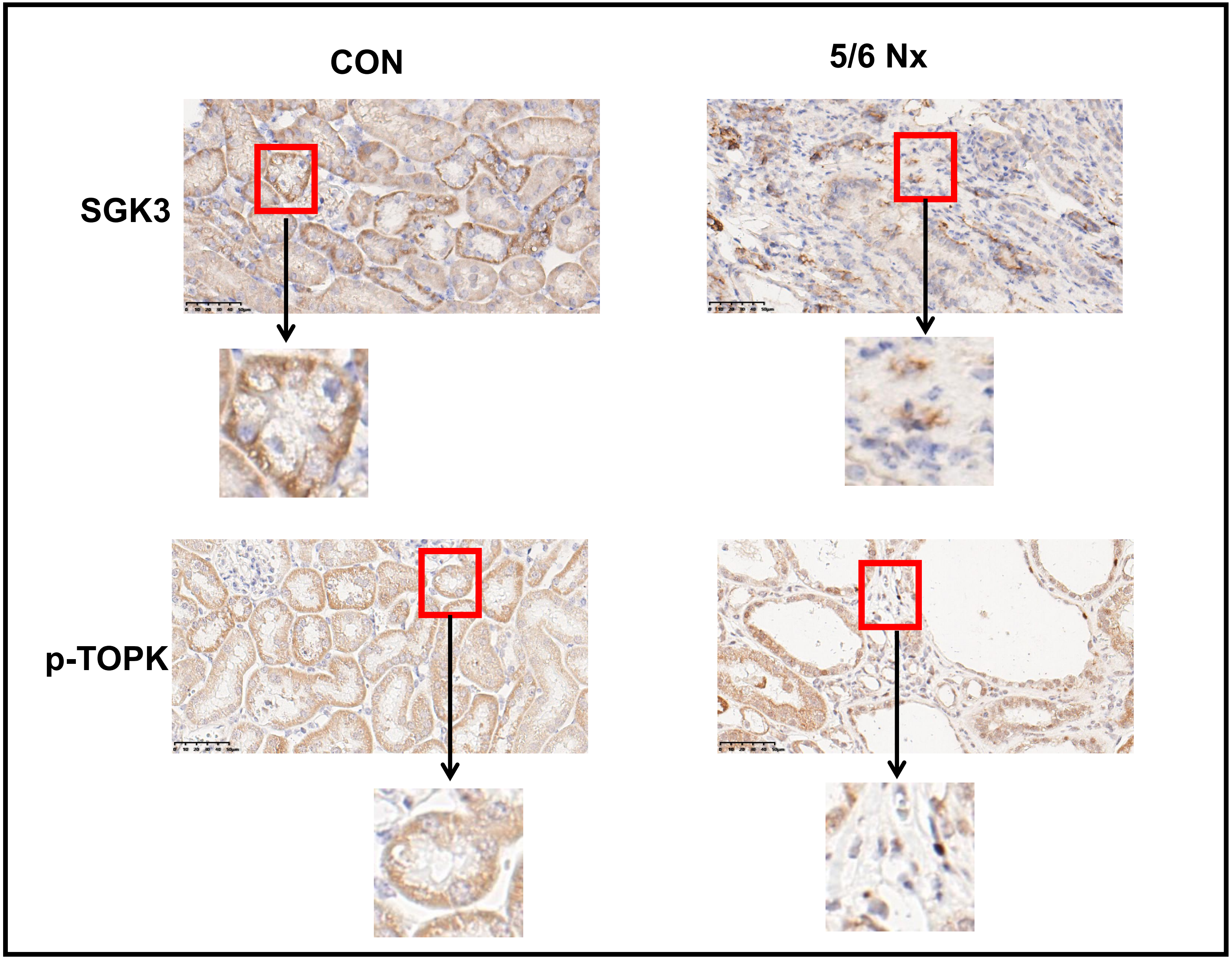

Figure4

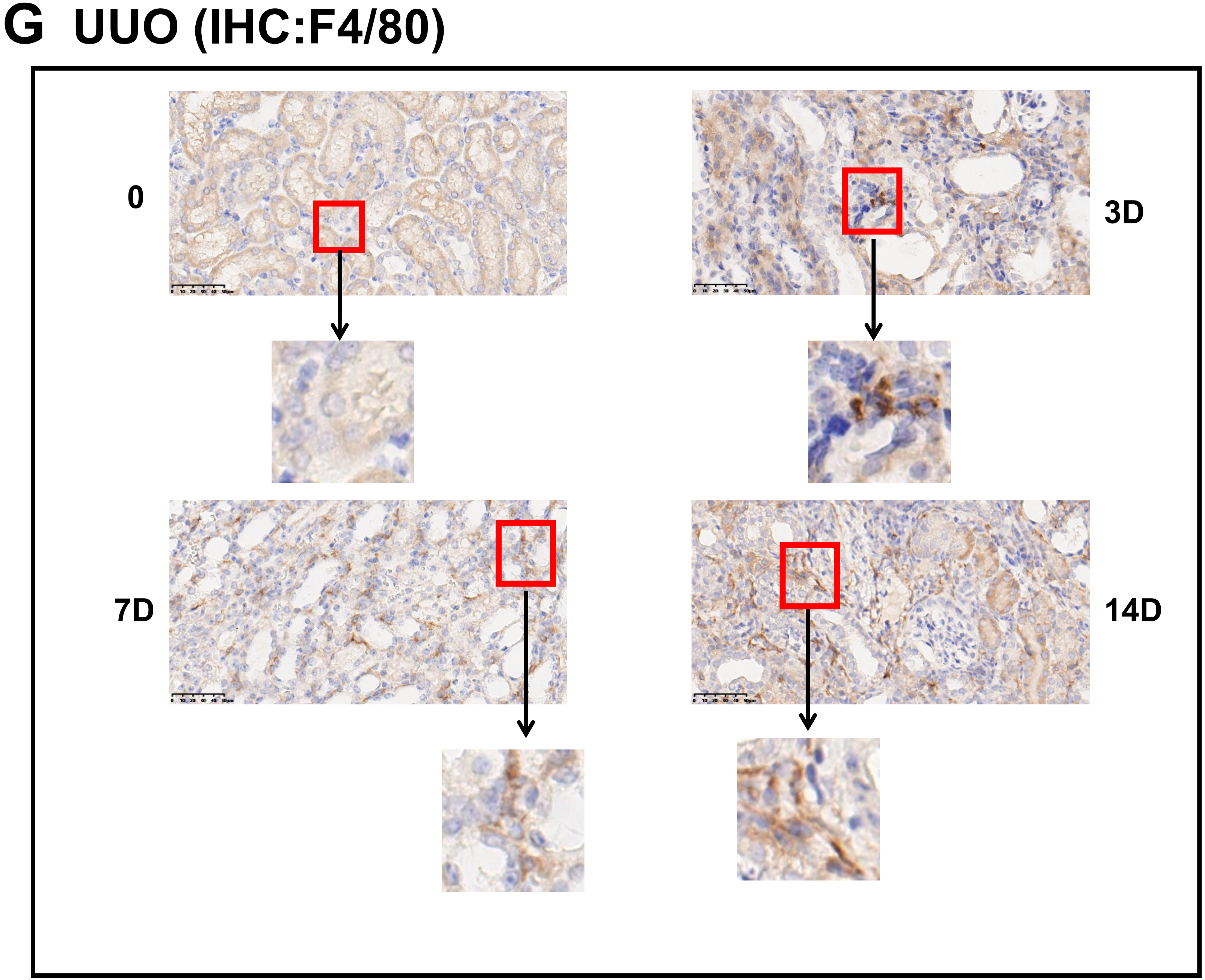

Figure4

G UUO (IHC:α-SMA)

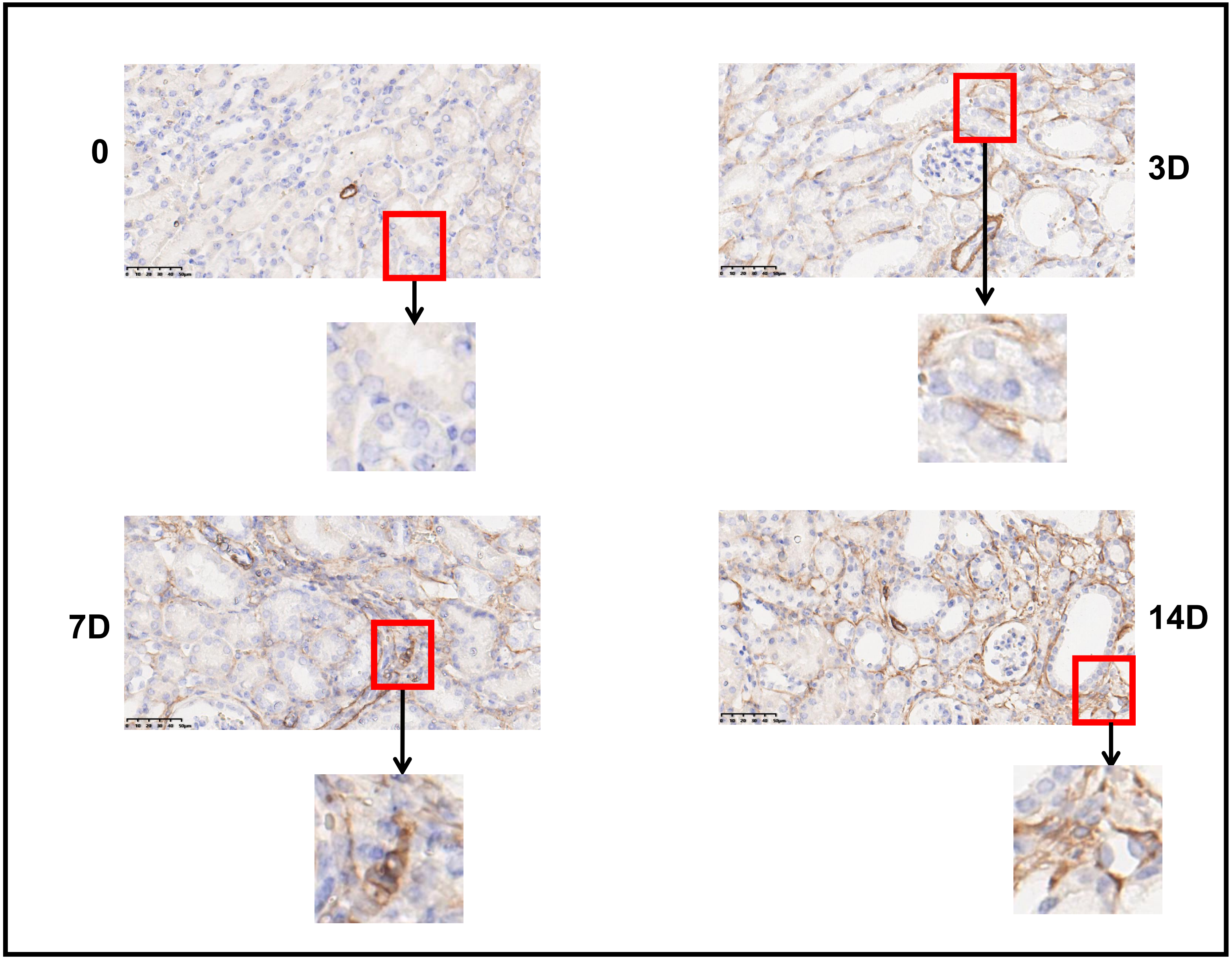

Figure4

G UUO (IHC:SGK3)

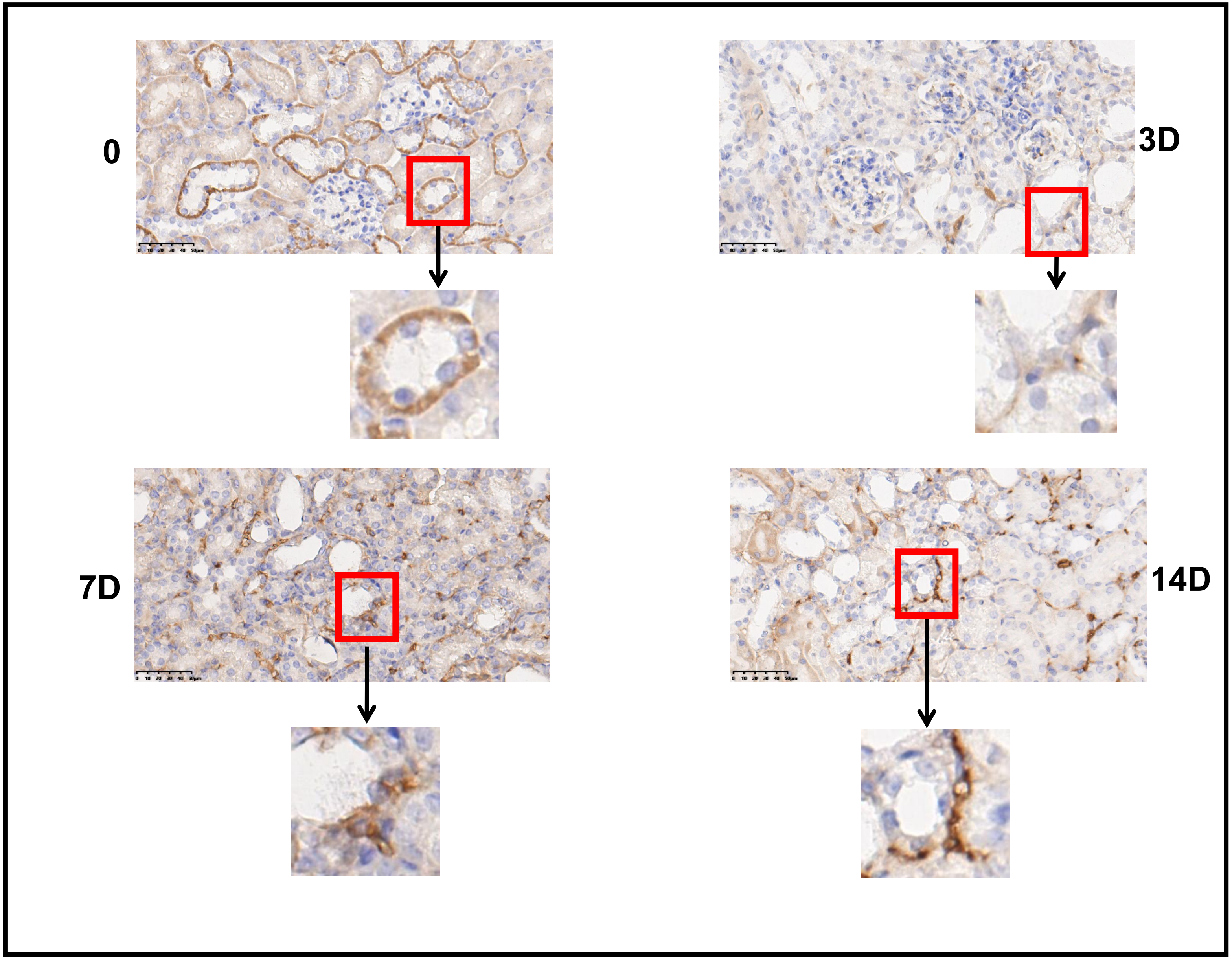

Figure4

G UUO (IHC:p-TOPK)

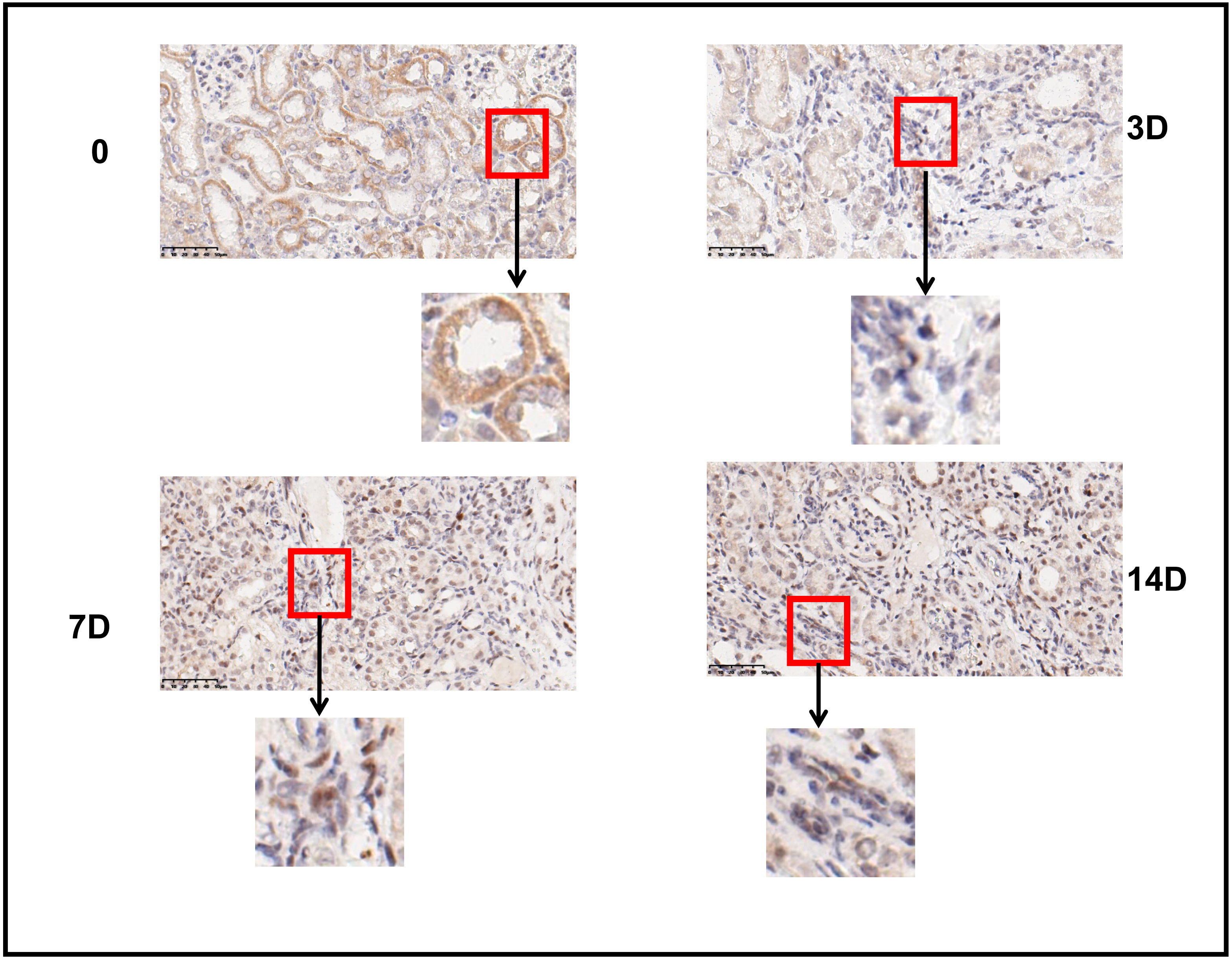

Figure5

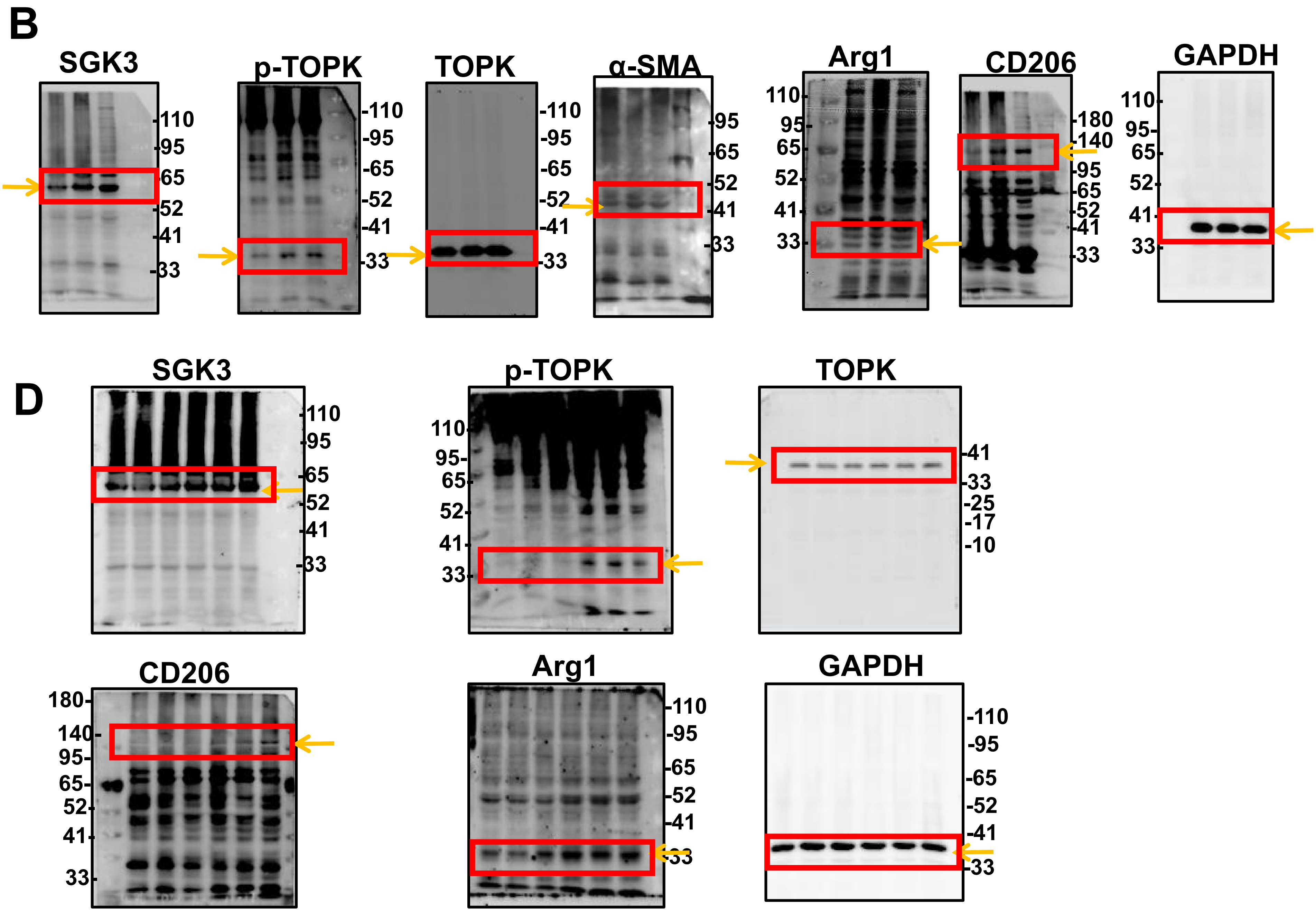
B
SGK3
p-TOPK
TOPK
α-SMA
Arg1
CD206
GAPDH
110
95
65
52
41
33
180
140
D
SGK3
p-TOPK
TOPK
CD206
Arg1
GAPDH
25
17
10

Figure5

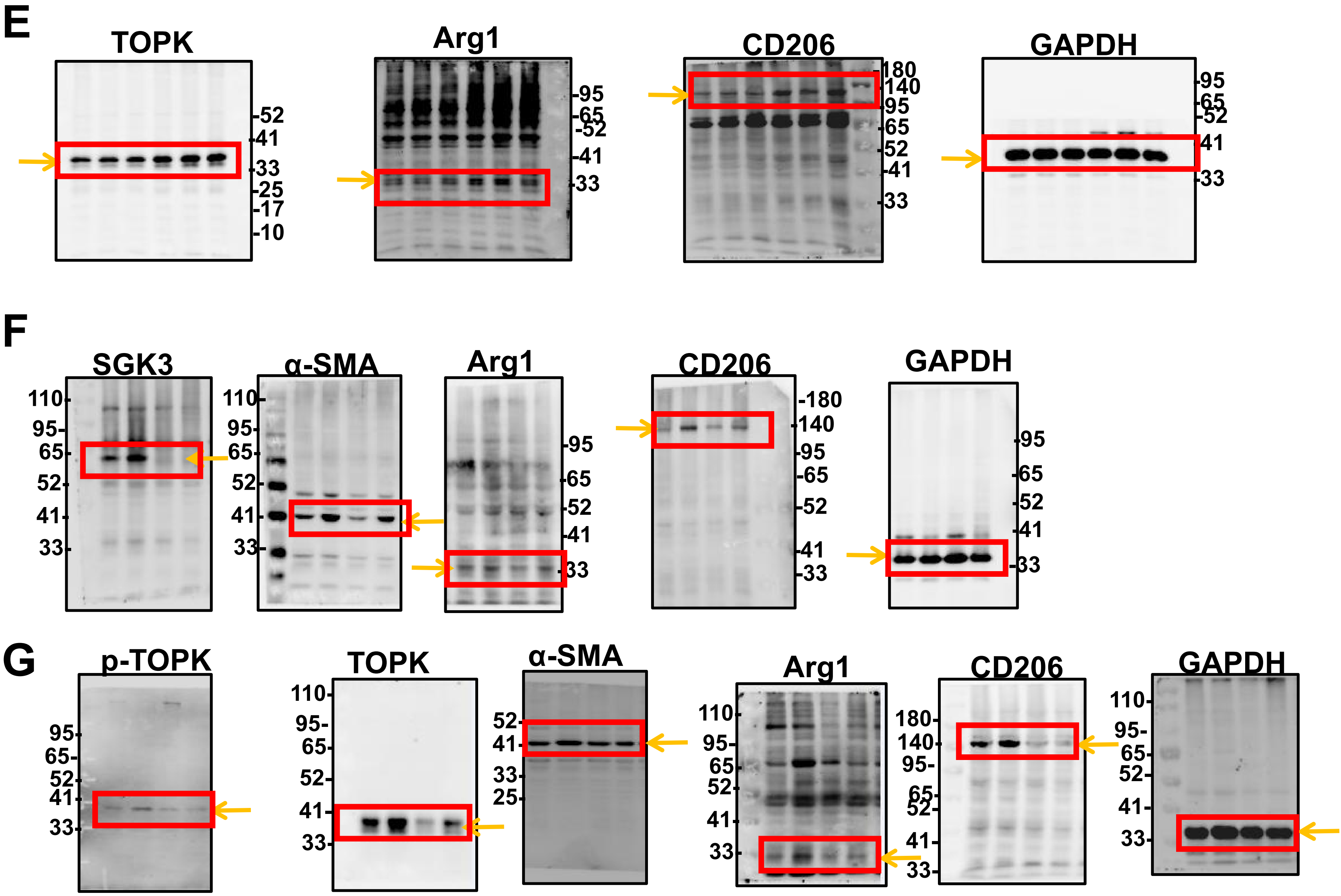
E
TOPK
-52
-41
-33
-25
-17
-10
Arg1
-95
-65
-52
-41
-33
CD206
180
140
95
65
52
41
33
GAPDH
95
65
52
41
33
F
SGK3
110
95
65
52
41
33
α-SMA
110
95
65
52
41
33
Arg1
95
65
52
41
33
CD206
-180
140
95
65
52
41
33
GAPDH
95
65
52
41
33
G
p-TOPK
95
65
52
41
33
TOPK
110
95
65
52
41
33
α-SMA
52
41
33
25
Arg1
110
95
65
52
41
33
CD206
180
140
95
65
52
41
33
GAPDH
110
95
65
52
41
33

Figure5

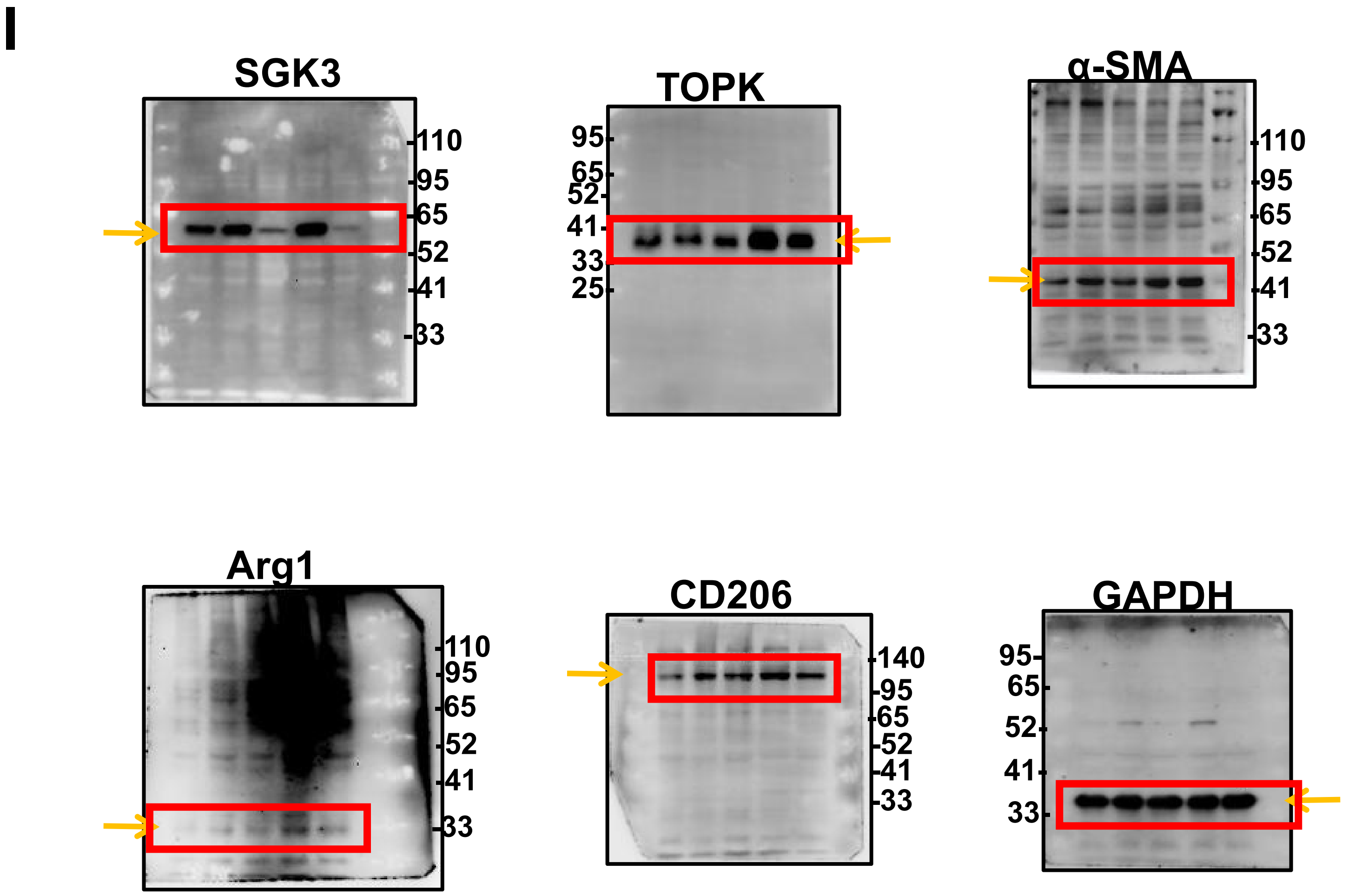
I
SGK3
110
95
65
52
41
33
TOPK
95
65
52
41
33
25
α-SMA
110
95
65
52
41
33
Arg1
110
95
65
52
41
33
CD206
140
95
65
52
41
33
GAPDH
95
65
52
41
33

Figure6

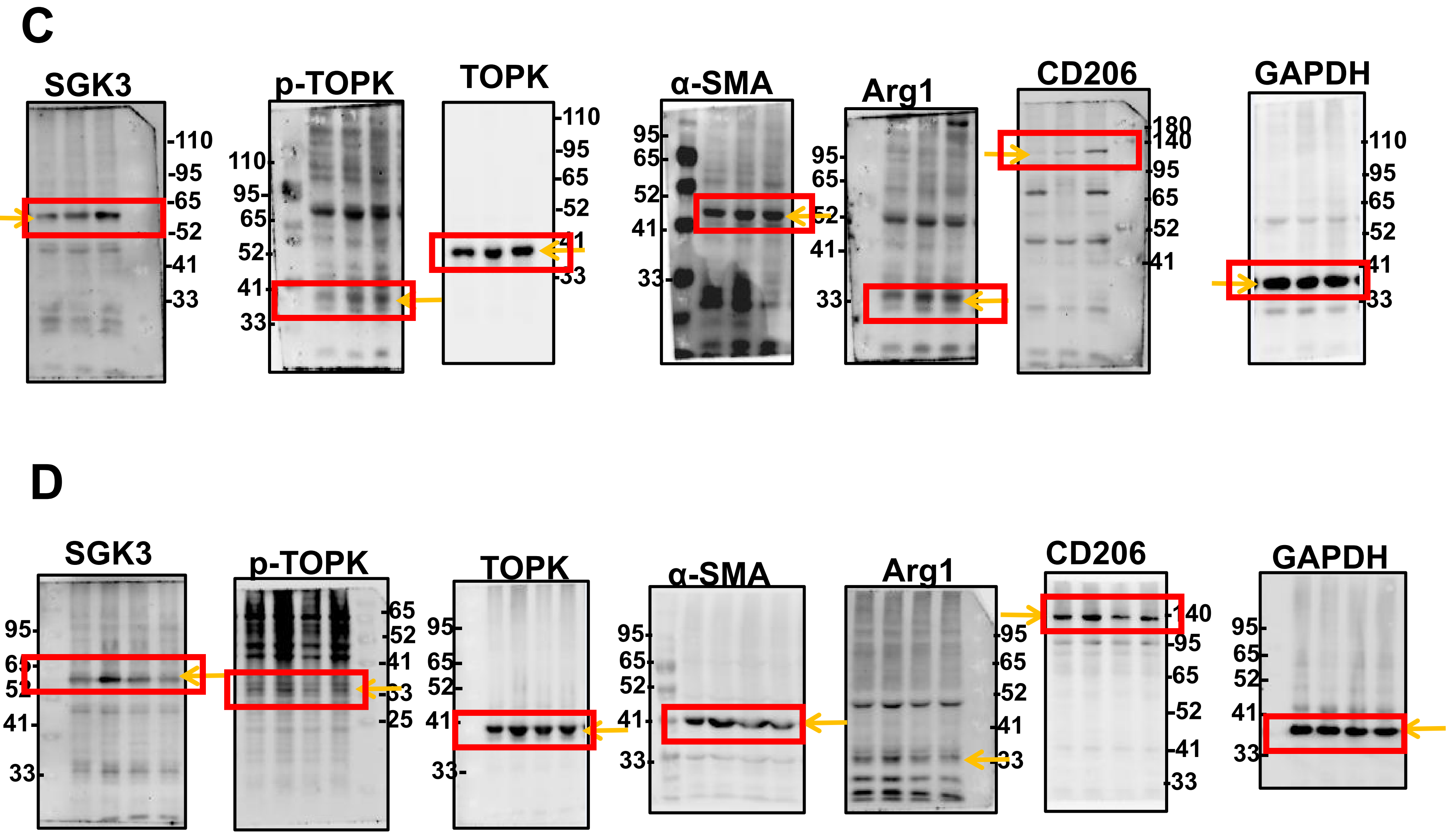
C
SGK3
p-TOPK
TOPK
α-SMA
Arg1
CD206
GAPDH
D
SGK3
p-TOPK
TOPK
α-SMA
Arg1
CD206
GAPDH

Figure6

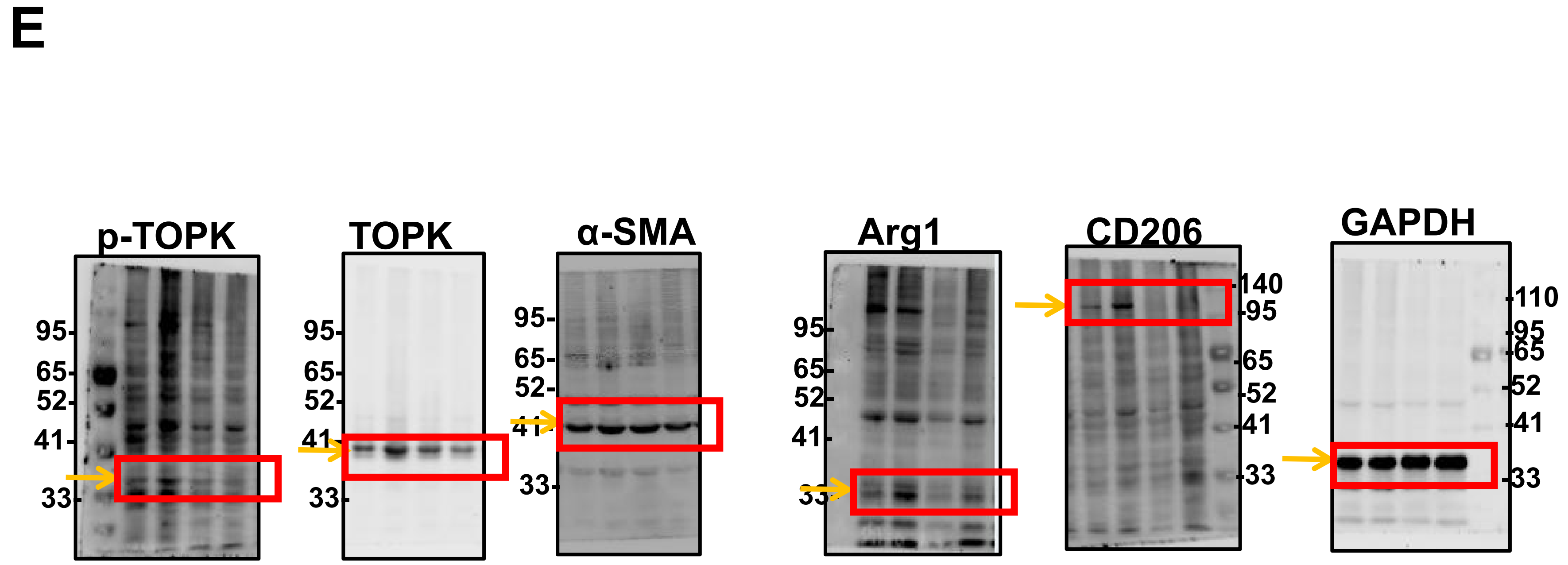

Figure7

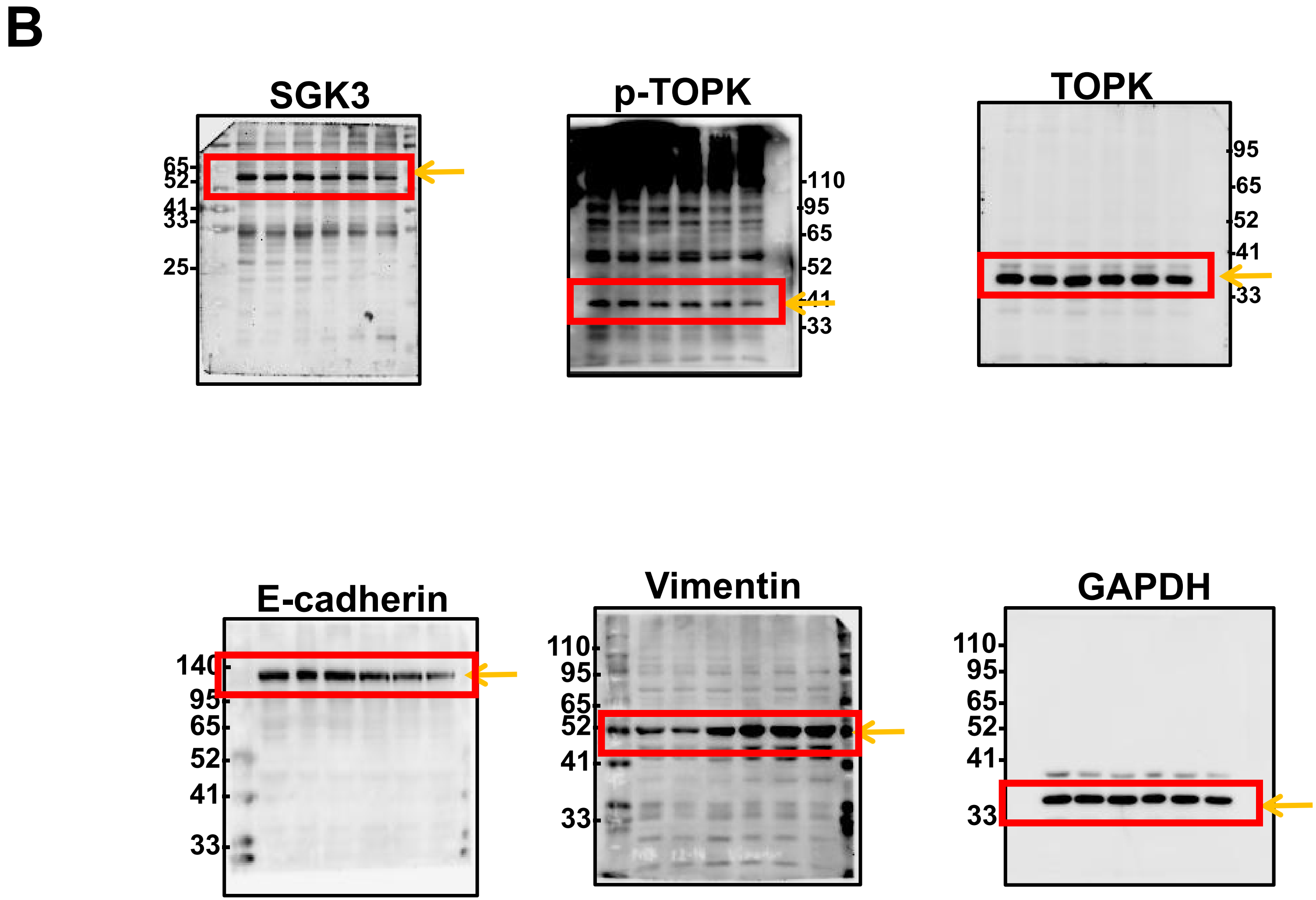
B
SGK3
65
52
41
33
25
p-TOPK
110
95
65
52
41
33
TOPK
95
65
52
41
33
E-cadherin
140
95
65
52
41
33
Vimentin
110
95
65
52
41
33
GAPDH
110
95
65
52
41
33

Figure7

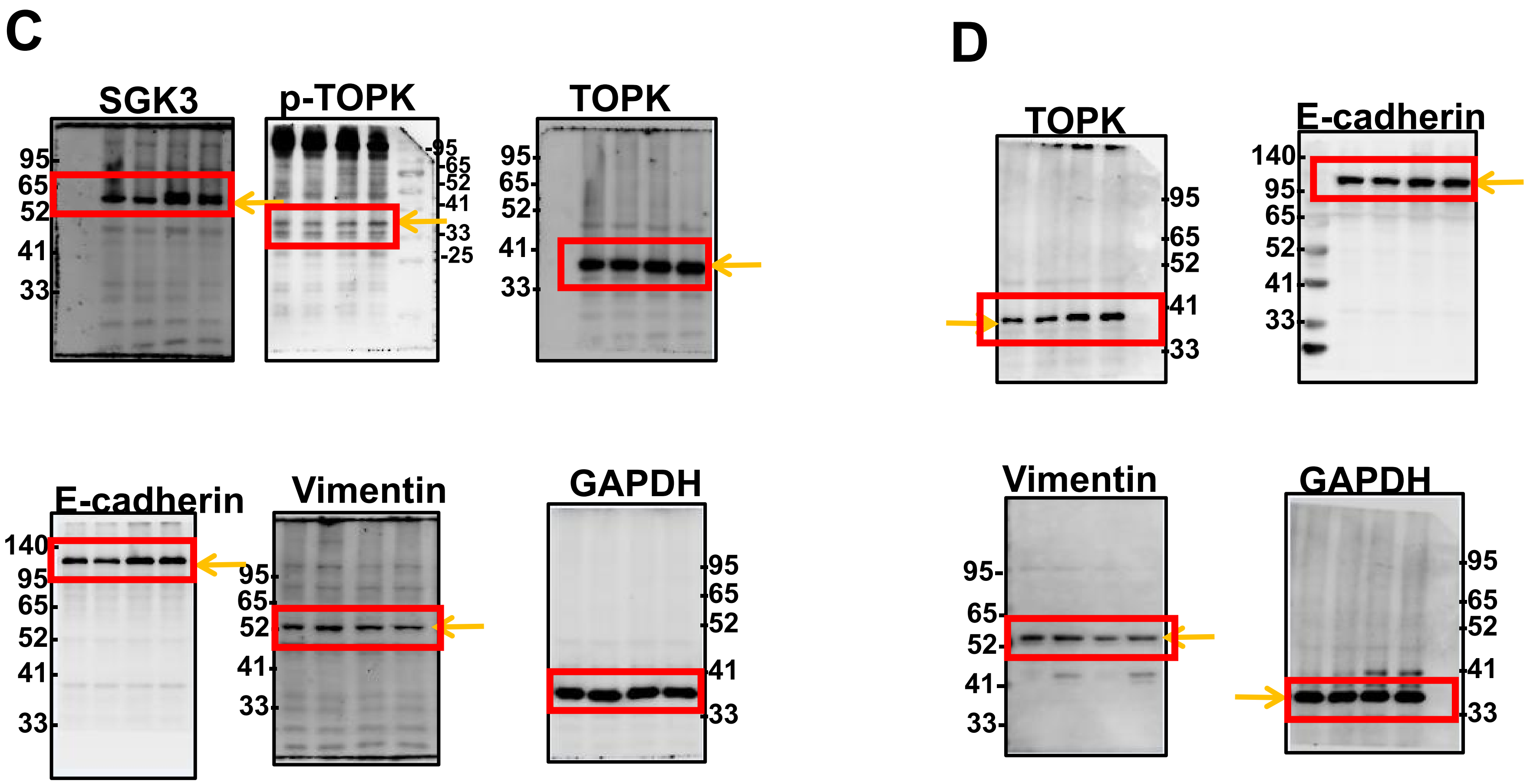
C
SGK3
p-TOPK
TOPK
95
65
52
41
33
25
E-cadherin
Vimentin
GAPDH
140
D
TOPK
E-cadherin
Vimentin
GAPDH
